# Novel Diazocrowns with Pyrrole Residue as Lead(II)Colorimetric Probes

**DOI:** 10.3390/ma14237239

**Published:** 2021-11-26

**Authors:** Błażej Galiński, Elżbieta Luboch, Jarosław Chojnacki, Ewa Wagner-Wysiecka

**Affiliations:** 1 Department of Chemistry and Technology of Functional Materials, Faculty of Chemistry, Gdańsk University of Technology, Narutowicza Street 11/12, 80-233 Gdańsk, Poland; blazej.galinski@pg.edu.pl (B.G.); elzluboc@pg.edu.pl (E.L.); 2 Department of Inorganic Chemistry, Faculty of Chemistry, Gdańsk University of Technology, Narutowicza Street 11/12, 80-233 Gdańsk, Poland; jaroslaw.chojnacki@pg.edu.pl

**Keywords:** azomacrocycle, pyrrole, synthesis, ion recognition, lead(II) complexation, chromoionophore

## Abstract

Novel 18- and 23-membered diazomacrocycles were obtained with satisfactory yields by diazocoupling of aromatic diamines with pyrrole in reactions carried under high dilution conditions. X-ray structure of macrocycle bearing five carbon atoms linkage was determined and described. Compounds were characterized as chromogenic heavy metal ions receptors. Selective color and spectral response for lead(II) was found in acetonitrile and its mixture with water. Complexation properties of newly obtained macrocycles with a hydrocarbon chain were compared with the properties of their oligoether analogs. The influence of the introduction of hydrocarbon residue as a part of macrocycle on the lead(II) binding was discussed. Selective and sensitive colorimetric probe for lead(II) in aqueous acetonitrile with detection limit 56.1 μg/L was proposed.

## 1. Introduction

Heavy metals—like lead(II)—are highly toxic to humans and bioaccumulate in aquatic systems having harmful effects on the environment [1,2,3]. Lead poisoning (saturnism) has been present throughout the history of mankind. Lead was one of the first metals widely used (e.g., fishing nets, domestic utensils, etc.) due to its ease of extraction and its ductility. Exposure to lead is often claimed to contribute to the fall of the Roman Empire. Mental disorders called “painter’s colic” or “painter’s madness” displayed by some of the great masters, including Michelangelo and Caravaggio, are also attributed to lead poisoning. The possibility of lead poisoning is also mentioned in the case of famous composers Beethoven and Händel [4,5]. Lead exposure causes dysfunction of blood and nervous systems. Lead is also easily precipitating in the brain, kidneys and reproductive system. Poisoning with lead can also cause anemia and brain damage and finally death [6,7,8]. Nowadays, lead poisoning is rarely seen in developed countries, but it still represents a major environmental problem in certain areas, also in tap water in communities with older service lines and older household plumbing containing lead. The regulatory guidelines’ value given for drinking water by the WHO is 0.01 mg/L [9].

As the most common methods of determination of lead(II) at trace levels, atomic absorption spectrometry (AAS) or inductively coupled plasma optical emission spectrometry (ICP-OES) can be mentioned. These and other analytical methods [10,11,12,13] enable accurate and precise detection and determination of lead(II) in various types of samples at different concentration levels, but there is still essential need for competitive, easy to use, low cost, fast and reliable methods which can be applied also in on site tests. These requirements are, in many cases, met by optical sensors [14,15,16] in which a selective ion receptor is incorporated in the sensing layer or acts as selective complexing reagent in a solution.

Lead(II) is a borderline acid in HSAB theory, forming complexes in various coordination modes with many ligands [17], also mixed with N,O macrocyclic ones [18]. Spectrophotometric and naked-eye detection and determination of lead(II) are often based on selective complex formation between a metal ion and a chromogenic sensing molecule with defined donor atom number and arrangement. For example, acyclic azo compound 1,5-dimethyl-2-phenyl-4-((2,3,4-trihydroxyphenyl)diazenyl)-1H-pyrazol-3(2H)-one was proposed for the spectrophotometric determination of lead(II) by a surfactant-sensitized method based on the ternary complexes formation with a detection limit of 0.3 μg/mL (1.45 × 10^−6^ M) [19]. Another example was described by Wang and Chen [20]. Azo dye was found to form a magenta colored complex with lead(II) in acetonitrile-water (1:1) mixture of stability constant value log*K* 4.66. Azoderivative was used in test strips for determination of lead(II) in untreated wastewaters and freshwaters at ppm level. In this respect, macrocyclic azo compounds, discriminating ions according to their size, serve as a group of molecules of interesting sensing properties and applications [21,22]. Due to presence of an azo moiety, macrocycle-metal cation interaction can be followed by the change of absorption spectra in the visible range of electromagnetic radiation [23,24,25,26,27,28]. Until now, a series of chromogenic crown ethers with two azo groups and phenol [23], pyrrole [24,25,26,27] or imidazole [28] residue as a part of macrocycle were synthesized and studied in our group as metal cation complexation agents in solution and as ionophores in membrane ion selective electrodes. Diazocrowns with pyrrole residue, bearing three nitrogen donor atoms (one of each azo group and nitrogen of pyrrole moiety), were described for the first time in 2003 [24]. Their functionalized derivatives were found to have high lead(II) affinity in solution and in membrane systems [24,25,26,27].

A property of these compounds, i.e., showing an easy to measure color change upon lead(II) complexation, prompted us to synthesize new macrocyclic derivatives of expected affinity towards heavy metal cations to obtain selective and sensitive probes for lead(II) detection and determination. Macrocyclic receptors with mixed N,O donor atoms bridged with oligoether or hydrocarbon linkage were investigated also to find out more about the possible nature of lead(II) binding by this class of compounds.

## 2. Materials and Methods

### 2.1. General

All chemicals of the highest available purity were purchased from commercial sources and used without further purification. TLC aluminum sheets covered with silica gel 60 F254 and glass plates 60 RP-18 F_254_ were purchased from Merck (Germany). For column chromatography, silica gel 60 (0.063–0.200 mm) from Merck (Germany) was used. ^1^H and ^13^C spectra were recorded on a Varian INOVA 500 spectrometer (Palo Alto, CA, USA) at 500 MHz and 125 MHz, respectively. Chemical shifts are reported as δ (ppm) values in relation to TMS. EILR and EIHR mass spectra of crowns were recorded on an AutoSpec Premier (Waters) instrument (Milford, MA, USA). For registration of ESI-LR spectra of lead(II) complexes and MS/MS experiments, API 3000 (Applied Biosystems, Warrington, Cheshire, UK) (equipped with ESI ion source), a triple-quadrupole mass spectrometer was used. FTIR spectra (film) were taken on a Nicolet iS10 apparatus (Thermo Fisher Scientific, Waltham, MA, USA). For UV–VIS measurements, an UNICAM UV 300 series apparatus (Spectronic Unicam, Leeds, UK) was used. Spectroscopic measurements were carried out in 1 cm quartz cuvettes in acetonitrile (LiChrosolv®, Merck, Germany). For recovery studies, Standard Reference Solution of lead(II) 1000 ppm (Merck, Germany) was used. Lead(II) concentration for comparative studies was determined using the ICP-OES method with iCAP 7400 Analyzer (Thermo Fisher Scientific, Waltham, MA, USA).

Glass microfiber filter (Whatman GF/C) (Schleicher & Schuell, Dassel, Germany) was used for test strips preparation. For optodes preparation PVC (Fluka, Switzerland), potassium tetrakis(4-chlorophenyl)borate (≥98%, Fluka, Switzerland), 2-nitrophenyl octyl ether (NPOE) (≥99%, Sigma-Aldrich, Switzerland), THF (≥99.5%, Sigma-Aldrich, St. Louis MO, USA) were used.

### 2.2. Synthesis of Crowns ***3*** and ***4***

#### 2.2.1. 1,5-bis(2-nitrophenoxy)pentane (**7**) and 1,10-bis(2-nitrophenoxy)decane (**8**)

Compounds **7** and **8** were prepared analogously as described in the literature [29,30]. A mixture of 2-nitrophenol (2.24 g, 16 mmol), 1,5-dibromopentane (2.07 g, 9 mmol) or 1,10-dibromodecane (2.70 g, 9 mmol) and anhydrous potassium carbonate (2.22 g, 16 mmol) in dry dimethylformamide (6 mL) were stirred and heated at 140 °C for 2 h. The mixture was diluted with cooled water (120 mL) to precipitate crude dinitro derivatives 7 or 8. Pure compounds **7** and **8** were obtained after crystallization from propan-2-ol (20 mL). **7**: yield 2.23 g (80%), light beige solid, mp 82–83 °C (lit. mp 83 °C) [30], **8**: yield 2.78 g (83%), light beige solid, mp 75–77 °C. (lit. mp 76 °C) [30].

#### 2.2.2. 1,5-bis(2-aminophenoxy)pentane (**5**) and 1,10-bis(2-aminophenoxy)decane (**6**)

Compounds **5** and **6** were obtained using protocols described in the literature [24,25,26,27]. The reaction mixture containing compound **7** (2.80 g, 8 mmol) or **8** (3.33 g, 8 mmol) and propan-2-ol (50 mL) together with a Pd/C catalyst was magnetically stirred and heated in an oil bath at 58 °C. Aqueous hydrazine solution (80%) was added to the reaction mixture in 4 portions (0.5 mL each). Five hours after the last portion of hydrazine was added, the solution was filtered off to separate the catalyst. The solvent was evaporated under the reduced pressure. Amines were crystallized from propan-2-ol. **5**: yield 2.00 g (87%), white flakes, mp 60–61 °C (lit. mp 61 °C) [30]. **6**: yield 2.70 g (95%), white flakes, mp 66–67 °C. (lit. mp 67 °C) [30].

Compounds **1** and **2** were synthesized analogously to previous protocol [24], and the identity of material was confirmed by comparison of TLC and spectral properties with original samples of crowns deposited in our lab.

#### 2.2.3. Preparation of New Diazocrown **3** and **4**

The synthesis of diazocrown **3** and **4** was based on a high dilution approach [24,25,26,27]. Three solutions were prepared:

- A: Diaminopodand **5** or **6** (1 mmol) and concentrated hydrochloric acid (0.5 mL) in water (20 mL) (DMF or THF in needed amount can be added to increase solubility of amines);
- B: Sodium nitrite (2 mmol) in water (30 mL);
- C: Pyrrole (1 mmol, 0.07 mL) and sodium hydroxide (0.20 g, 5 mmol) in water (30 mL).

All solutions were cooled in an ice bath to 0–5 °C. Solution B was added portionwise to solution A to obtain the bisdiazonium salt. The combined solutions A-B were left for 30 min in an ice bath. After this time, solutions A-B and C were added dropwise to deionized water (300 mL) at pH ~10 (NaOH), within 30 min, ensuring intensive stirring of the reaction system. The pH was controlled during the addition of solutions to the reaction container. After 2 h, the ice bath was removed, and the mixture was left for 24 h at room temperature. The precipitate was filtered off under reduced pressure. Products were isolated by column chromatography using initially dichloromethane and finally dichloromethane:acetone (10:1, *v/v*) as eluent. Compound **3** crystallizes from petroleum ether, **4** crystallizes from n-hexane.

Compound **3**: Yield 0.092 g (25%), red solid. mp 254–257 °C (with decomposition). TLC: Rf = 0.91 (chloroform), 0.47 (dichloromethane:n-hexane, 100:1). ^1^H NMR (d-chloroform, 500 MHz), δ (ppm): 1.96–1.99 (4H, m), 2.09–2.14 (2H, m), 4.24 (4H, t, J = 5.5 Hz), 7.01 (2H, t, J = 7.5 Hz), 7.07 (2H, d, J = 8.8 Hz), 7.07 (2H, s), 7.40 (2H, dt, J1 = 8 Hz, J2 = 1.5 Hz), 7.79 (2H, dd, J1 = 8.5 Hz, J2 = 1.5 Hz), 10.17 (1H, s, NH). ^13^C NMR (d6-DMSO, 125 MHz) δ (ppm): 23.9, 30.8, 40.4, 40.6, 40.8, 68.5, 114.8, 115.4, 116.5, 121.1, 133.8, 141.2, 146.7, 157.3. FTIR (crystalline film, cm^−1^): 3450, 2940, 2871, 1585, 1488, 1456, 1371, 1275, 1232, 1150, 756. UV–VIS (acetonitrile): λ_max_ = 508 nm, ε_max_ = 2.22 × 10^4^. HRMS [EI]: found 375.1702 calculated for: C_21_H_21_N_5_O_2_ 375.1695.

Compound **4**: Yield 0.131 g (30%), red solid, mp 223–225 °C (with decomposition). TLC: Rf = 0.74 (chloroform), 0.51 (dichloromethane:n-hexane, 100:1). ^1^H NMR (d6-DMSO, 500 MHz) δ (ppm): 1.27–1.42 (12H, m), 1.76 (4H, t, J = 6.6 Hz), 4.17 (4H, t, J = 6.1 Hz), 7.05 (2H, t, J = 7.6 Hz), 7.18 (2H, s), 7.24 (2H, d, J = 8.2 Hz), 7.41–7.44 (4H, m), 11.40 (1H, s, NH). ^13^C NMR (d6-DMSO, 125 MHz) δ (ppm): 25.3, 28.0, 28.4, 28.8, 40.2, 69.5, 115.1, 116.7, 117.9, 121.2, 132.4, 143.8, 147.2, 155.6. IR (film, cm^−1^): 3418, 2920, 2851, 1588, 1489, 1455, 1389, 1277, 1241, 1158, 749. UV–VIS (acetonitrile): λ_max_ = 495 nm, ε_max_ = 2.15 × 10^4^. HRMS [EI]: 445.2481 calculated for: C_26_H_31_N_5_O_2_ 445.2478.

NMR and mass spectra of **3** and **4** are shown in Supplementary Materials (Figures S1 and S2).

#### 2.2.4. Preparation of Solid Complexes of Crowns **1**–**4** with Lead(II) Perchlorate

Samples for MS and FTIR analyses were prepared by dissolving of the crowns and lead(II) perchlorates in a molar ratio 3:2 in acetonitrile and left for solvent evaporation. ^1^H NMR and MS spectra are included in Supplementary Materials (Figures S1, S2 and S11–S14).

### 2.3. Lipophilicity Determination

The lipophilicity values (log P_TLC_) of chromoionophores **1**–**4** were estimated by TLC method [31,32] using reverse phase chromatography (RP18) with a mixture of methanol: water (9:1, *v/v*) as the mobile phase. As standards bis(1-butylpentyl)adipate (BBPA) (≥98.0%, Sigma Aldrich, Switzerland), 2-nitrophenyl octyl ether (NPOE) (≥99.0%, Sigma Aldrich, Switzerland), bis(2-ethylhexyl)sebacate (DOS) (≥97.0%, Sigma Aldrich, Germany), bis(2-ethylhexyl)phthalate (DOP) (≥97.0%, Fluka, Switzerland) and di-n-butylphthalate (DBP) (≥97.0%, Fluka, Switzerland) were used.

### 2.4. X-ray Structure Determination

Diffraction intensity data for **3** were collected on an IPDS 2T dual beam diffractometer (STOE & Cie GmbH, Darmstadt, Germany) at 120.0(2) K with MoKα radiation of a microfocus X-ray source (GeniX 3D Mo High Flux, Xenocs, Sassenage, France, 50 kV, 1.0 mA, and λ = 0.71069 Å). Investigated crystals were thermostated under a nitrogen stream at 120 K using the CryoStream-800 device (Oxford CryoSystem, Oxford, UK) during the entire experiment. Data collection and data reduction were controlled by using the X-Area 1.75 program (STOE, 2015). Due to low absorption coefficient no absorption correction was performed. The structure was solved using intrinsic phasing implemented in SHELXT and refined anisotropically using the program packages Olex2 [33] and SHELX-2015 [34]. Positions of the C–H hydrogen atoms were calculated geometrically taking into account isotropic temperature factors. All H-atoms were refined as riding on their parent atoms with the usual restraints, including N-H atom (with AFIX 43) which does not take part in hydrogen bonding. Structure of **3** was refined with no special treatment. Crystallographic data reported in this paper have been deposited with the Cambridge Crystallographic Data Centre as supplementary publication No. CCDC 2081633 (Appendix A). The data can be obtained free of charge from The Cambridge Crystallographic Data Centre via www.ccdc.cam.ac.uk/structures (accessed on 12 October 2021). Details are included in Supplementary Materials including Figures S15 and S16 and Table S2).

### 2.5. Cation Binding Studies

Cation binding for crowns **1**–**4** was studied by UV–VIS titration in acetonitrile. The stock solutions of crowns (~10^−4^ M) and metal perchlorates (~10^−2^ M) were prepared by weighting the respective quantities of them and dissolving in acetonitrile in volumetric flasks. The cation binding constant values (log*K*) were calculated with the use of OPIUM [35] program on the basis of titration experiment data. The influence of interfering ions on spectrophotometric response towards lead(II) was expressed as the absolute value of relative response *RR%* = |[(*A* − *A*_0_) / *A*_0_]| × 100%, where *A*_0_ stands for absorbance of solution of crowns **1**–**4** in the presence of lead(II) perchlorate (equimolar to crown amount) and *A* absorbance value measures just after addition of interfering metal perchlorate in 10-fold excess to lead(II) perchlorate.

Limits of detection (LOD) for lead(II) were calculated using equation: LOD = 3σ/k, where σ is the standard deviation of the blank and k is the slope of the linear function A = f([Pb(II)]).

### 2.6. Preparation of Sensing Layers

#### 2.6.1. Test Strips

Solutions of newly obtained crowns **3** (2.9 × 10^−4^ M) and **4** (3.1 × 10^−4^ M) in acetonitrile were poured into a chromatographic chamber, into which a strip of glass microfiber filter was placed, similar to the procedure used with the TLC plates. After 5 min, strips were taken out, and solvent was evaporated in a stream of hot air.

#### 2.6.2. Optodes

The membrane cocktail was prepared by weighing out potassium tetrakis(4-chlorophenyl)borate (0.5 mg, 0.3% *w*/*w*), crown ether **3** or **4** (1.0 mg, 0.6% *w*/*w*), polyvinyl chloride (50 mg, 32.2% *w*/*w*) and NPOE (104 mg, 66.9% *w*/*w*). The components were dissolved in 1 mL of THF, and aliquots (90 μL) were deposited onto glass plates (9 × 45 mm) which were washed before use with nitric acid (10^−1^ M), acetone and propan-2-ol. After solvent evaporation (24 h), the resulting sensing membranes were used for naked-eye detection of lead(II).

## 3. Results and Discussion

### 3.1. Synthesis

**Scheme 1:**
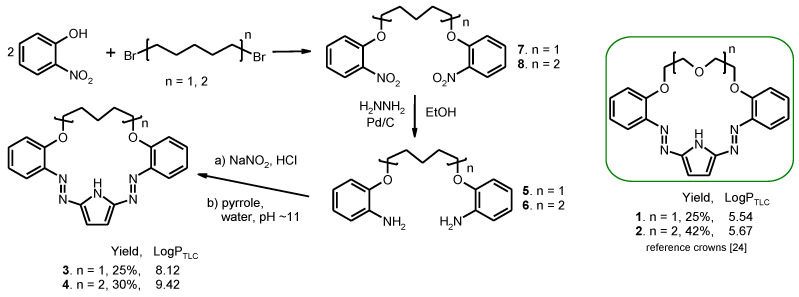
Synthetic route of new macrocycles **3** and **4** and formulas of reference crowns **1** and **2** (right) [24]. Yields of macrocyclization and determined values of logP_TLC_ are given.

New 18- and 23-membered macrocycles (compounds **3** and **4**) were synthesized by azocoupling of bisdiazonium salts with pyrrole analogously to previously described protocols (Scheme 1) [24,25,26,27]. Reaction carried out under high dilution conditions gave desired compounds with macrocyclization yield 25 and 30% for **3** and **4**, respectively, which seems to be within typical yields (19–42%) for this group of compounds [24,25,26,27].

For the first time for this class of compounds, lipophilicity values (log P_TLC_) are reported here. The values of log P_TLC_ obtained by the RP-TLC method [30,31] (Scheme 1, Figure S10) as could be expected are higher for hydrocarbon chain bearing macrocycles **3** and **4** than for their oligoether analogs **1** and **2**.

Described earlier [24,25,26,27] pyrrole derivatives bearing oligoether chain as a part of macrocycle were found to form complexes with metal cations in acetonitrile and acetonitrile-water mixtures with preferential lead(II) cations affinity among all investigated metal cations (alkali, alkaline earth and heavy metal ions). Compounds **1** and **2** in acetonitrile (Scheme 1), treated here as reference compounds, form with lead(II) perchlorate double-sandwich type complexes of 3:2 stoichiometry (crown:Pb) of relatively high stability constants values (log K) 18.10 ± 0.01 and 21.10 ± 0.09 [25], respectively. The previous studies [24,25,26,27] showed that lead(II) complex formation, including spectral and color changes, is dependent among others on the macrocycle size and the type of substituents both in benzene rings and the modification of the oligoether moiety. Here we tested other than the previously described modification of the skeleton of the pyrrole bearing chromogenic crown ethers, namely, introducing a hydrocarbon chain instead of oligoether linkage.

### 3.2. X-ray Structure of ***3***

Suitable for X-ray analysis, crystals of macrocycle **3** were obtained by crystallization from petroleum ether solution and studied by single crystal X-ray diffraction (for details see Supplementary Materials).

**Figure 1:**
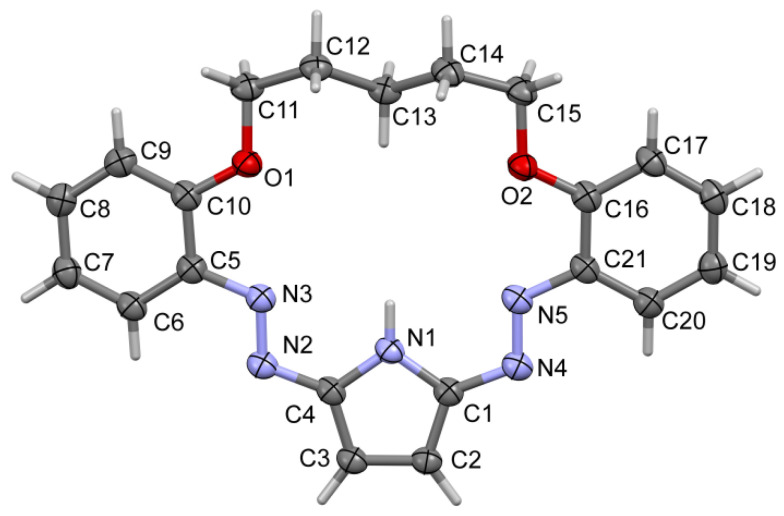
Molecular view of **3** showing atom labelling scheme.

Compound **3** forms thin plate, red crystals, with symmetry of the orthorhombic system, the space group *Pbca* (no. 61). The asymmetric unit contains one molecule, and the whole unit cell is built from eight molecules, Z = 8. Most of the bond lengths and angles are in the expected ranges (Figure 1, details in Supplementary Materials), including the hydrocarbon chain C11–C15.

**Figure 2:**
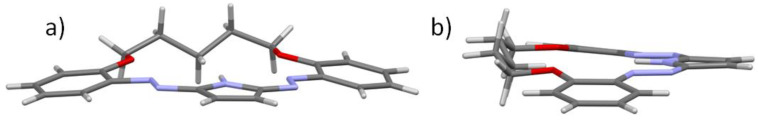
Projections of compound **3**, showing kinked conformation of the molecule: (**a**) front view, (**b**) side view. Subsequent torsions in the linker chain are (ap, sc, -ap, ap, -sc, -ap). More precisely: C10-O1-C11-C12 170.67 (15), O1-C11-C12-C13 57.5 (2), C11-C12-C13-C14 -174.57 (16), C12-C13-C14-C15 179.94 (16), C13-C14-C15-O2 -57.9 (2), C14-C15-O2-C16 -170.87 (16).

Based on short interatomic distances, one may assume the mostly double character of N2-N3, N4-N5 bonds and the aromatic character of the pyrrolic ring. The molecule is essentially flat with an expected folding of the -C_5_H_10_- linker (ap, sc, -ap, ap, -sc, -ap), (Figure 2), (ap—antiperiplanar, sc—synclinal). The dihedral angle between the phenyl rings C5-C10 and C16-C21 is equal to 17.56 (9)°, which shows some deviation from planarity. The internal cavity offers Lewis base donor electron pairs on O1, O2, N3 and N5 atoms (hydrogen bond acceptors) as well as N-H group which is a potential hydrogen bonding donor. The size of the crown interior may be estimated by diagonal O…N atom distances which are equal to O1…N5 5.720 Å and O2…N3 5.602 Å. It is noteworthy that the bond C13-H13A is directed to the center of the hole, similarly to the N-H bond.

Crystal packing is dependent mainly on weak non-covalent interactions since no hydrogen bonding is available for the molecules. Shortest inter-ring (i.e., ring centroids) distances as short as 3.3603(8) are reported by PLATON, but this is observed for centers of 18- or 22-membered macrocyclic rings and should be disregarded. Nevertheless, some true stackings of pyrrole N1-C1-C4 and benzene C16-C21 rings are also found, (Centroid distance 3.7913(11) Å, dihedral angle between the planes α = 7.92(10)°, slippage 2.093 Å). Other interactions are mainly of the van der Waals type. Crystal packing in 3 is shown in Figure S16. For details see Supplementary Data.

### 3.3. Heavy Metal Cation Complexation Studies

**Figure 3:**
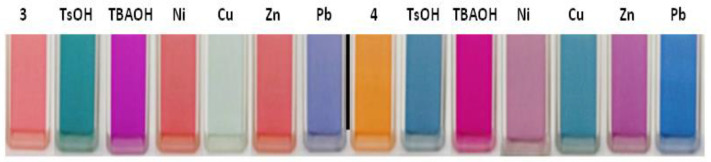
Qualitative probes showing color change of crowns **3** (2.01 × 10^−5^ M) and **4** (3.75 × 10^−5^ M) solutions (acetonitrile) in the presence of excess (added as solid substances, saturated solution) of *p*-toluenesulfonic acid (TsOH), tetra-n-butylammonium hydroxide (TBAOH) and nickel(II), copper(II), zinc(II) and lead(II) perchlorates in acetonitrile (photo was taken just after addition of the excess of acid, base and perchlorates).

On the basis of qualitative tests carried out in acetonitrile, it was found that compounds **3** and **4** show a color change in the presence of: *p*-toluenesulfonic acid (TsOH), tetra-n-butylammonium hydroxide (TBAOH), nickel(II), copper(II), zinc(II) and lead(II) perchlorates (Figure 3). No changes were observed in the presence of alkali and alkaline earth metal perchlorates.

**Figure 4:**
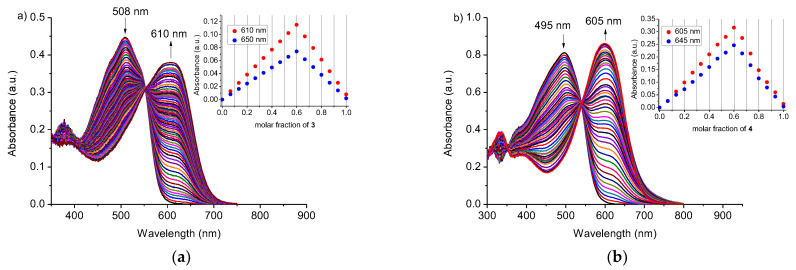
Changes in absorption spectra of crowns **3** and **4** upon titration with lead(II) perchlorate: (**a**) **3** (c_3_ = 2.01 × 10^−5^ M; c_Pb_ = 0–2.61 × 10^−5^ M); (**b**) **4** (c_4_ = 3.75 × 10^−5^ M; c_Pb_ = 0–5.75 × 10^−5^ M) in acetonitrile. Insets: Job’s plots for lead(II) perchlorate and crowns **3** and **4** in acetonitrile.

As it is shown in Figure 3, the color of solutions of macrocycles **3** and **4** changes in the presence of heavy metal perchlorates. For both compounds, the presence of zinc(II) and nickel(II) perchlorates causes comparable color changes, just after their addition, to red for **3** and to purple for **4**. The presence of copper(II) turns the color of solutions to blue. Besides copper(II), significant color change is observed in the presence of lead(II) perchlorate: from red to deep blue for **3** and from orange to blue for **4**. Color changes are the result of the change of UV–VIS spectra, namely, formation of absorption band (Figure 4) at 610 and 605 nm, (spectral shift 102 and 110 nm), for **3** and **4,** respectively. Clear isosbestic points can indicate one complex under equilibrium.

Job’s plots for lead(II) systems with **3** and **4** (Figure 4, insets) show apparent x_max_ value at 0.6 corresponding to the formation of 3:2 (crown:Pb) complex in acetonitrile. Analysis of UV–VIS titration data with OPIUM [35] software also gives the best fit for 3:2 (crown:Pb) system with stability constant (log*K*) values 19.22 ± 0.05 and 18.37 ± 0.01 for **3** and **4,** respectively. The same model of lead(II) binding was proposed for previously studied macrocycles **1** and **2** [25] (Table S1). When comparing log*K* values (Table S1) for 18-membered crowns **1** and **3,** it is worth noting that lead(II) is bound stronger by macrocycle **3** (log*K* 19.22 ± 0.05) with hydrocarbon chain than by oligoether bearing derivative **1** (log*K* 18.10 ± 0.01). Contrary to this, 21-membered crown **2** binds lead(II) stronger (log*K* 21.0 ± 0.09) than compound **4** of 23-membered ring (log*K* 18.37 ± 0.01).

Thus, it can be assumed that incorporation of a long, flexible hydrocarbon chain can affect the size of the macrocycle cavity of **4** and makes it more comparable to cavity of 18-membered crowns **1** and **3**.

**Figure 5:**
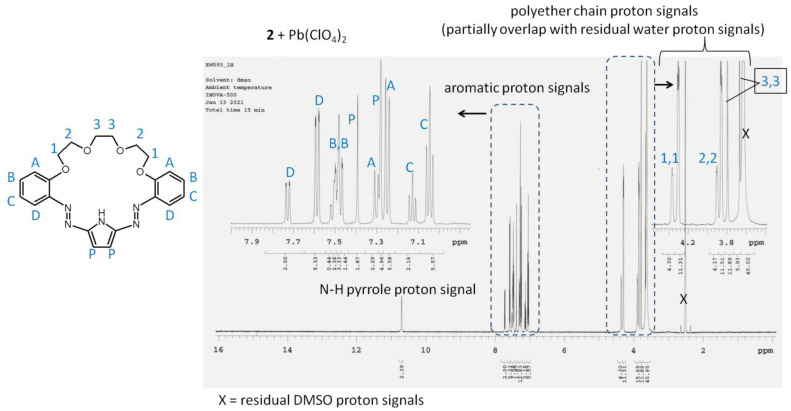
^1^H NMR spectrum of **2** (1.1 × 10^−2^ M) in the presence of lead(II) perchlorate, molar ratio of components—crown:lead(II) perchlorate 3:2 (DMSO-d_6_).

Complexes of 3:2 stoichiometry—triple-decker sandwich type—were reported for a significant number of systems including various types of ligands and metal centers. This type of complexes are common in transition metal chemistry [36] and lanthanides [37]. Moreover, for barium, such sandwich complex was reported [38]. An analogous model of lead(II) binding can be assumed for investigated here diazocrowns bearing pyrrole moiety. It can be confirmed by X-ray measurements, but unfortunately so far, suitable monocrystals of lead(II) complexes have not been obtained yet. ^1^H NMR spectra of crown **2** and **4** in the presence of lead(II) perchlorate were registered in DMSO-d_6_ due to low solubility of crowns in acetonitrile in required for experiment concentrations (Figure 5, Figures S3a,b and S4a,b). As solvent type can strongly influence the complexation equilibrium, spectra registered in DMSO do not bring the clear evidence for 3:2 complex formation in DMSO. However, two sets of signals in ^1^H NMR spectra of compounds **2** and **4** registered in the presence of lead(II) perchlorate are observed. It means that in solution, ligands are not equivalent, at least on the NMR timescale. The resonances of “free” crowns **2** and **4** and their lead(II) complexes are shown in spectra of expanded range in Figures S5, S3b and S4b. The downfield shift of the signal of proton (marked as D in Figures S3b and S4b) of aromatic ring in *ortho* position to azo group can be a result of the engagement of one nitrogen of azo group in complex formation. Oxygen atoms of oligoether residue or linking hydrocarbon moiety seem also to participate in lead(II) binding as signals of these protons being also shifted and not equivalent. Interestingly, in the spectrum registered for compound **2,** significant change for signal of pyrrole CH protons (Figure 5) is observed. In the presence of lead(II) perchlorate, two signals are observed. One of them is slightly (0.05 ppm) upfielded—which could point to deprotonation of the pyrrole moiety upon lead(II) complexation. The second signal of pyrrole residue, observed at 7.36 ppm, is shifted towards the higher ppm values of 0.14 comparing the signal in spectrum of the “free” crown. This, coming along with deshielded N-H proton signal (+1.48 ppm), can point to the formation of a stronger hydrogen bonding system in complex than that in “free” crown. The shift of signal of N-H proton in hydrocarbon linked derivative **4** upon lead(II) complexation is lower (+0.26 ppm).

The investigation of nature of metal complexes and organometallic compounds with MS spectrometry is often challenging [39]. MS spectra of complexes of crowns **1**–**4** with lead(II) were registered using ESI ionization method in positive ions mode (acetonitrile as a solvent). Observed under measurement conditions peaks of m/z 1636 for compound **3** (Figure S1e) and m/z 1847 for compound **4** (Figure S2e) can correspond to complexes of 3:2 stoichiometry of composition corresponding to two ionized crowns, one non-ionized macrocycle, two lead(II) cations and one perchlorate anion (Table 1). Analogous peaks were observed in spectra of complexes of crown **1** (m/z 1644) and crown **2** (m/z 1776). Peak m/z 1948 in spectrum of **4** corresponds to three macrocyles with one being ionized, two lead cations and two perchlorate anions. Analogous peaks were also detected in ESI spectra of crowns **1** and **2**: m/z 1745 and 1876, respectively. This confirms the possibility of formation of complexes of proposed stoichiometry in acetonitrile solution, which was proposed on the basis of Job’s plots obtained from UV–VIS measurements. However, besides mentioned above ions, under mass spectra measurement conditions, a number of other ionic species were detected (Table 1) that may be the result yet another ionization of complexes under MS measurements (e.g., peaks corresponding to 3:3 stoichiometry) and the fragmentation of the above. Theoretically calculated and experimental isotope patterns of assigned peaks are shown in Supplementary Data (Figure S11–S14).

In the FTIR spectrum registered for lead(II) complex of **4** (not shown here), the band corresponding to perchlorate ion is localized at 1104 cm^−1^ suggesting that counter ion is not engaged in complex formation.

**Figure 6:**
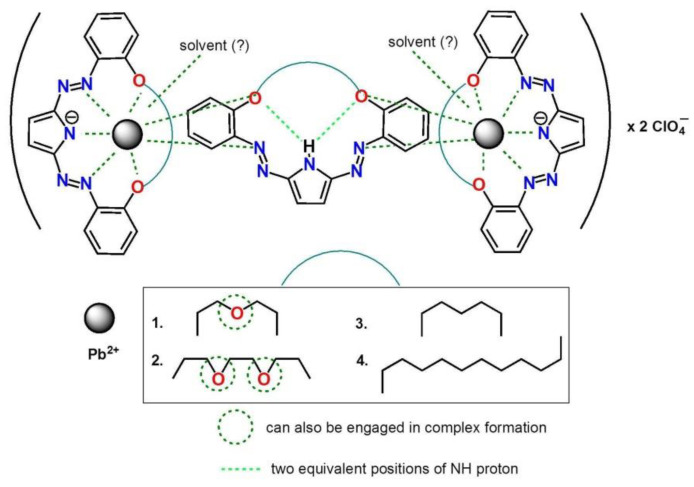
Proposed, simplified model (as triple-decker complex) of lead(II) binding by diazocrowns **1**–**4** bearing pyrrole moiety.

On the basis of the above, we propose as probable, but still assumed, a binding model of lead(II) as triple-decker complex for oligoether bearing crowns and for hydrocarbon linked macrocycles (Figure 6).

**Figure 7:**
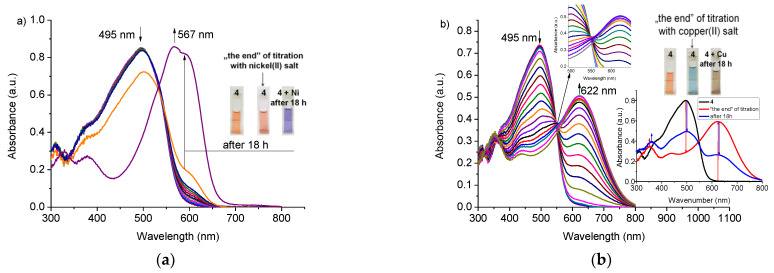
Changes in UV–VIS spectra of **4** upon titration with: (**a**) nickel(II) (c_4_ = 3.75 × 10^−5^ M, c_Ni_ = 0–1.35 × 10^−3^ M)—band of the highest absorbance at 567 nm corresponds to absorption spectrum of titrated solution registered after 18 h; (**b**) copper(II) (c_4_ = 3.75 × 10^−5^ M, c_Cu_ = 0–4.74 × 10^−5^ M) perchlorates in acetonitrile. Insets: Color change of solutions and change of UV–VIS spectrum for **4**—Cu system after 18 h.

For crown **4,** the presence of nickel(II) and zinc(II) perchlorates in acetonitrile results in the creation of absorption bands of complexes at 567 and 565 nm, respectively. However due to the spectral changes within time (exemplified with titration trace for **4** shown in Figure 7a for nickel(II) and in Figure S5 for zinc (II)), the determination of reliable value of stability constant values of complexes of **4** with zinc(II) and nickel(II) perchlorates was not possible under measurements conditions. For **3** nickel(II) and zinc(II) perchlorates presence causes negligible changes in UV–VIS absorption spectra under titration measurements conditions.

In case of titration with copper(II), perchlorate changes within the time of experiment were observed for **3** and **4**, however, different than for nickel(II) and zinc(II) perchlorates (exemplified with titration trace for **4** in Figure 7b). The clear isosbestic point (550 nm) loses its sharpness (Figure 7b, inset) within the progress of the titration experiment. It can point to the change of complexation equilibrium or the occurrence of other process in the presence of copper(II) perchlorate in acetonitrile. After 18 h, the solution turns to brown, which might be an effect of the changes in the chromophore system resulting from the redox process. The nature, including identification of products of interactions pyrrole bearing diazocrowns with copper(II) in acetonitrile, is under elaboration by our group.

**Figure 8:**
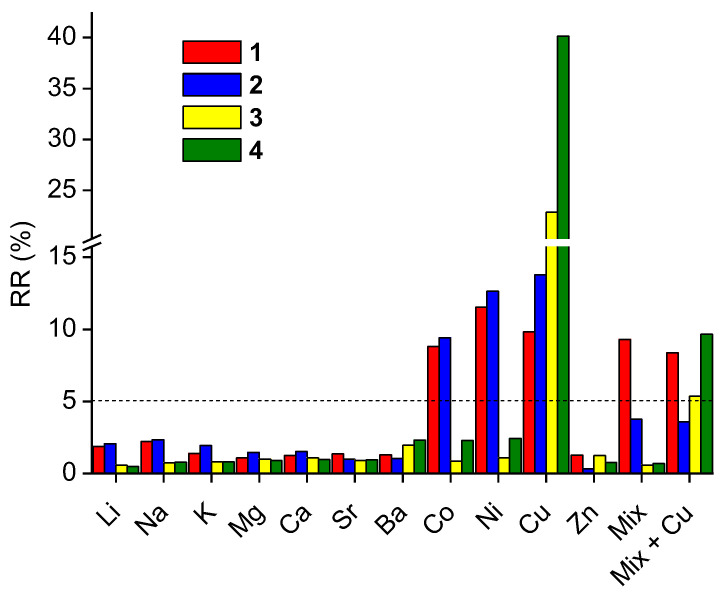
Interferences from several metal perchlorates, used in 10-fold excess, on the spectrophotometric response towards lead(II) for **1** (2.54 × 10^−5^ M) at 605 nm, **2** (2.73 × 10^−5^ M) at 610 nm, **3** (2.08 × 10^−5^ M) at 610 nm and **4** (2.63 × 10^−5^ M) at 605 nm in acetonitrile (Mix—mixture of metal perchlorates without copper).

The effect of interfering metal perchlorates on the spectral response of **3** and **4** towards lead(II) in comparison with their oligoether analogs **1** and **2** was investigated. In competing studies, the absorbance of solutions of crowns **1**–**4** in the presence of lead(II) perchlorate (equimolar amount) was measured before (A_0_) and just after (A) addition of interfering metal perchlorate in 10-fold excess to lead(II) perchlorate. The influence of tested interfering metal perchlorates on spectrophotometric response towards lead(II), as the relative response RR%, is presented in Figure 8. For most investigated metal cations, the effect of their presence can be treated as negligible, as the RR% value is below 5%.

The strongest effect on the absorbance of solution crowns **1**–**4** has copper(II) perchlorate and thus soft copper(II) must be considered as the main interfering metal perchlorate in acetonitrile and also as an oxidant. Alkali and alkaline earth metal cations stronger influence the optical response of oligoether bearing crowns **1** and **2**, crowns **3** and **4** bearing hydrocarbon linkage. This can be connected with the hard nature of these cations stronger interaction with the hard oxygen donor atom of the oligoether chain [26]. Moreover, heavy metal cations—cobalt(II) and nickel(II)—affect the spectrophotometric response of crowns **1** and **2** more that in it is observed for macrocycles **3** and **4**. The individual spectral response given as a change of absorbance of each crown in the presence of the equimolar amount of lead(II) perchlorate and the influence of the presence of 10-fold molar excess of interfering metal perchlorates is shown in Figure S6a–d.

The limits of detection (LOD) for lead(II) in acetonitrile determined from UV–VIS measurements were found to be 5.84 × 10^−6^ M (n = 10) and 6.22 × 10^−6^ M (n = 20) with linear response range 6.23 × 10^−6^–2.26 × 10^−5^ M for **3** (Figure S7). For 23-membered crown, **4** LODs were determined as 7.63 × 10^−7^ M (n = 10) and 9.17 × 10^−7^ M (n = 20) with a linear response range 1.05 × 10^−6^–2.22 × 10^−5^ M (Figure S8).

### 3.4. Possible Applications

**Figure 9:**
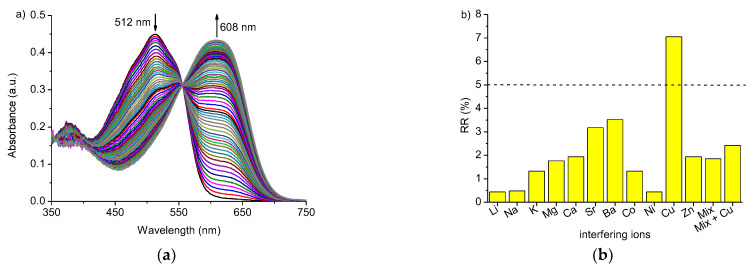
(**a**) Changes in the absorption spectrum of crown **3** (c_3_ = 2.13 × 10^−5^ M) upon titration with lead(II) perchlorate (c_Pb_ = 0“4.94 × 10^−4^ M); (**b**) interferences from several metal perchlorates at 10-fold molar excess on spectrophotometric response of 3 (c_3_ = 2.13 × 10^−5^ M) at 608 nm at equimolar (to crown) amount of lead(II) perchlorate in acetonitrile:water (9:1, *v/v*) solution at pH 5.

Promising results obtained in the acetonitrile solution prompted us to check the possibilities of using compound **3** as colorimetric lead(II) sensor in the presence of added water. Spectral changes upon titration of **3** with lead(II) perchlorate in acetonitrile:water solution (9:1, *v/v*) at pH 5 (HCl) are shown in Figure 9a. Under such measurement conditions, the spectral changes are significant (bathochromic shift of 96 nm) with a clear isosbestic point suggesting two species under equilibrium. In the mixed solvent system, at pH 5, the effect of an interfering metal cation, including heavy metal cations, is minor than in the acetonitrile solution (Figure 9b). For the most interfering cation in acetonitrile—copper(II)—the RR% value is below 8%. Interferences from several metal perchlorates, used in 10-fold excess, on the spectrophotometric response (change of absorbance) towards systems containing an equimolar amount of lead(II) perchlorate for **3** (2.13 × 10^−5^ M) at 608 nm at equimolar (to crown) amount of lead(II) perchlorate in acetonitrile:water (9:1, *v/v*) solution at pH 5 are shown in Figure S6e.

The linear response range A = f([Pb(II)]) was determined within 5.00 × 10^−^^7^–1.20 × 10^−^^5^ M (R^2^ = 0.9950) with LOD 2.71 × 10^−^^7^ M (Figure S8a). the linear response range can be widened to 5.00 × 10^−^^6^–1.15 × 10^−^^4^ M (R^2^ = 0.9995) using semi the logarithmic scale A = f(log[Pb(II)]), however, with an increase of the limit of detection to 2.97 × 10^−^^6^ M (Figure S9). It is worth noting that LODs values are lower in the water containing system than in pure acetonitrile.

For testing the possibility of using **3** as lead(II) sensor, a certain amount of Standard Reference Solution of lead(II) (1000 ppm) was diluted with water and added to acetonitrile solution of crown **3** to maintain 9:1 (*v/v*) solvent mixture at pH 5 (NaOH). The concentration of lead(II) was determined, using calibration curve A = f([Pb(II)]), as 5.06 × 10^−7^ M showing recovery 102.73% (concentration determined with ICP-OES: 4.93 × 10^−7^ M). Semilog calibration curve in this case did not allow the determination of lead(II) concentration as it is below LOD. The influence of the sample matrix of drinking water was checked using **3** for lead(II) determination in spiked tap water samples. Recoveries (%), collected in Table 2, are within 97.67–102.42 depending on the type of calibration curve.

**Figure 10:**
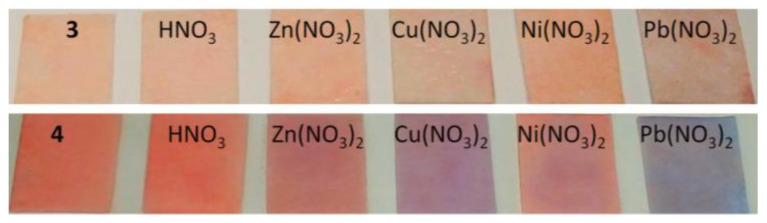
Changes of the color of test strips (glass filter) with adsorbed crowns **3** (2.9 × 10^−4^ M) and **4** (3.1 × 10^−4^ M) after immersing them for 15 s in solutions of (from left): deionized water, nitric acid (10^−1^ M) and metal nitrates (10^−2^ M) at pH 5.

Newly obtained macrocycles **3** and **4** were also preliminarily tested as components of ion-sensitive optical layers. Test strips bearing crown **4** show color changes in the presence of heavy metal cations nitrates (10^−2^ M) opposite to material obtained with the use of crown **3** (Figure 10).

**Figure 11:**
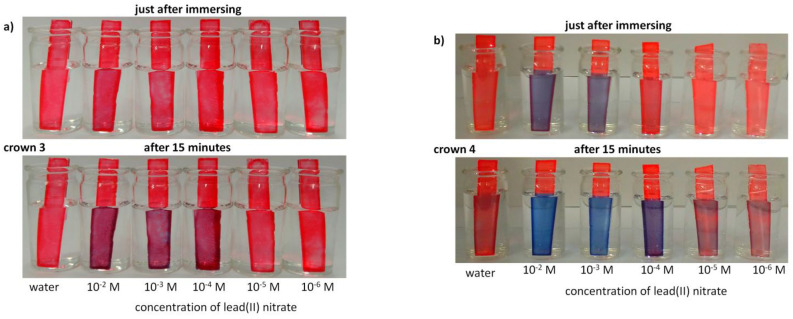
Color change of PVC based optodes bearing crown (**a**) **3** and (**b**) **4** upon immersion in solutions (pH 5) of lead(II) nitrate of different concentrations and, for comparison, in deionized water.

Crowns **3** and **4** were also incorporated into PVC-based optodes to test them as potential ionophores for lead(II) detection in water. Results are shown in Figure 11. Sensing material with crown **4** as chromoionophore shows a more distinct color change from red to blue both just after immersing in lead(II) containing solution and after some time.

The preliminary results presented above show that novel lipophilic macrocycles **3** and **4** can be potentially considered as lead(II) receptors in a mixed water containing solvent system and in aqueous solutions. Currently, work in our group is focused on more detailed studies (polymer matrix type, plasticizer, lipophilic salt, and amount of chromoionophore) on the possibilities of immobilization of macrocycles **1**–**4** to obtain lead(II) sensitive optical layers. Results will be published in a specialized analytical chemistry journal.

## 4. Conclusions

Novel 18- and 23-membered diazocrowns with hydrocarbon chain in macrocycle—compounds **3** (C5) and **4** (C10)—were obtained with satisfactory macrocyclization yields in reactions carried out under high dilution technique. For the first time for this class of macrocycles, lipophilicity values were given—important parameter characterizing compounds as candidates for new sensing materials (e.g., optodes). Macrocycles interact with metal cations in acetonitrile showing, analogously to their oligoether analogs, lead(II) selectivity. The introduction of hydrocarbon linkage in compounds **3** and **4** does not affect very strongly the mode and the strength of lead(II) binding comparing oligoether analogs **1** and **2** but makes them more selective in an acetonitrile and mixed acetonitrile-water system. The long, flexible ten carbon atoms linkage probably affects the size of the macrocylic cavity, and thus, lead(II) affinity for 23-membered crown is similar to 18-memered analog **1**. Crown **3** was successfully used as selective and sensitive colorimetric probe for lead(II) determination in organic solvent with the addition of water with limit of detection 2.71 × 10^−7^ M (56.1 μg/L). The proposed system can be regarded as competing with relatively expensive atomic spectroscopy methods. The obtained results have led the current work in our group to be focused on using crowns **1**–**4** as potential chromoionophores for lead(II) selective optodes, which will be published elsewhere.

## Figures and Tables

**Table 1:** Ionic species in ESI MS (positive mode) spectra of complexes of crowns **1**–**4** with lead(II).

Crown	Model	m/z *	Figure
1	[3(C_20_H_18_N_5_O_3_)^−^][3Pb^2+^][2ClO_4_^−^]	1950	S11a
	[2C_20_H_19_N_5_O_3_][(C_20_H_18_N_5_O_3_)^−^][2Pb^2+^][2ClO_4_^−^]	1745	S11b
	[2(C_20_H_18_N_5_O_3_)^−^][C_20_H_19_N_5_O_3_][2Pb^2+^][ClO_4_^−^]	1644	S11c
	[2(C_20_H_18_N_5_O_3_)^−^][2Pb^2+^][ClO_4_^−^]	1267	S11d
	[(C_20_H_18_N_5_O_3_)^−^][Pb^2+^]	584	S11e
2	[3(C_22_H_22_N_5_O_4_)^−^][3Pb^2+^][2ClO_4_^−^]	2082	S12a
	[2C_22_H_23_N_5_O_4_][(C_22_H_22_N_5_O_4_)^−^][2Pb^2+^][2ClO_4_^−^]	1876	S12b
	[2(C_22_H_22_N_5_O_4_)^−^][C_22_H_23_N_5_O_4_][2Pb^2+^][ClO_4_^−^]	1776	S12c
	[2(C_22_H_22_N_5_O_4_)^−^][2Pb^2+^][ClO_4_^−^]	1355	S12d
	[(C_22_H_22_N_5_O_4_)^-^][Pb^2+^]	628	S12e
3	[3(C_21_H_20_N_5_O_2_)^−^][3Pb^2+^][2ClO_4_^−^]	1944	S13a
	[2(C_21_H_20_N_5_O_2_)^−^][C_21_H_21_N_5_O_2_][2Pb^2+^][ClO_4_^−^]	1638	S13b
	[2(C_21_H_20_N_5_O_2_)^−^][2Pb^2+^][ClO_4_^−^]	1263	S13c
	[(C_21_H_20_N_5_O_2_)^−^][C_21_H_21_N_5_O_2_][Pb^2+^]	957	S13d
	[(C_21_H_20_N_5_O_2_)^−^][Pb^2+^]	582	S13e
4	[3(C_26_H_30_N_5_O_2_)^−^][3Pb^2+^][2ClO_4_^−^]	2154	S14a
	[2C_26_H_31_N_5_O_2_][(C_26_H_30_N_5_O_2_)^−^][2Pb^2+^][2ClO_4_^−^]^-^	1948	S14b
	[2(C_26_H_30_N_5_O_2_)^−^][C_26_H_31_N_5_O_2_][2Pb^2+^][ClO_4_^−^]	1847	S14c
	[2(C_26_H_30_N_5_O_2_)^−^][2Pb^2+^][ClO_4_^−^]	1403	S14d
	[(C_26_H_30_N_5_O_2_)^−^][Pb^2+^]	652	S14e

* Value corresponding to the peak of the highest intensity in model isotope pattern.

**Table 2:** Determination of lead(II) in spiked tap water samples using crown **3** as colorimetric probe.

Water Sample	Added Pb(II) (mol/dm^3^)	Found Pb(II) (mol/dm^3^)		Recovery (%)	
		A = f([Pb(II)])	A = f(log [Pb(II)])	A = f([Pb(II)])	A = f(log [Pb(II)])
Tap water 1	no addition	<LOD		-	
	2.00 × 10^−6^	1.95 × 10^−6^	-	97.68	-
	4.00 × 10^−6^	3.94 × 10^−6^	-	98.58	-
	8.00 × 10^−6^	8.19 × 10^−6^	7.81 × 10^−6^	102.42	97.67
	2.00 × 10^−5^	-	1.97 × 10^−5^	-	98.54
	4.00 × 10^−5^	-	4.05 × 10^−5^	-	101.18
	8.00 × 10^−5^	-	8.14 × 10^−5^	-	101.77
Tap water 2	no addition	<LOD		-	
	2.00 × 10^−6^	2.04 × 10^−6^	-	102.19	-
	4.00 × 10^−6^	4.03 × 10^−6^	-	100.84	-
	8.00 × 10^−6^	8.10 × 10^−6^	7.89 × 10^−6^	101.29	98.68
	2.00 × 10^−5^	-	2.01 × 10^−5^	-	100.59
	4.00 × 10^−5^	-	3.96 × 10^−5^	-	99.12
	8.00 × 10^−5^	-	8.06 × 10^−5^	-	100.73

- out of linear response range.

## Data Availability

Data sharing is not applicable.

## Supplementary Materials

The following are available online at https://www.mdpi.com/article/10.3390/ma14237239/s1. 1. Spectra of compounds **3** and **4** and lead(II) complexes: Figure S1a. ^1^H NMR of **3** (d-chloroform); Figure S1b. ^13^C NMR of **3** (DMSO-d6); Figure S1c. LRMS (EI) of **3**; Figure S1d. HRMS (EI) of **3**; Figure S1e. Fragmentation pattern for lead(II) complex (ESI—positive ions mode) of **3** and part of mass spectrum presenting isotopic lead(II) peaks with peak of the highest intensity m/z 1636; Figure S2a. ^1^H NMR of **4** (DMSO-d6); Figure S2b. ^13^C NMR of **4** (DMSO-d_6_); Figure S2c. LRMS (EI) of **4**; Figure S2d. HRMS (EI) of **4**; Figure S2e. Part of ESI-LR (positive ions mode) spectrum of lead(II)complex of **4** comparison of model of isotopic peaks for peak of m/z 1848. 2. Complexation studies—spectroscopic methods: Table S1. Stability constant (log*K*) values of diazocrowns lead(II) complexes **1**–**4** in acetonitrile; Figure S3a. 1H NMR spectrum of **2** (1.6 **×** 10^−2^ M) (DMSO-d_6_); Figure S3b. Top: ^1^H NMR spectrum of **2** (1.6 × 10^−2^M) in the presence of 10-fold excess of lead(II) perchlorate; bottom: comparison of the spectral pattern of proton signals in free crown **2** and its spectrum in the presence of 10-fold excess lead(II) perchlorate (DMSO-d_6_); Figure S3d ^1^H NMR spectrum of **2** (1.1 × 10^−2^M) in the presence of of lead(II) perchlorate, molar ratio of components—crown: lead(II) perchlorate 3:2 (DMSO-d_6_); Figure S4a. ^1^H NMR spectrum of **4** (1.6 × 10^−2^ M) (DMSO-d_6_); Figure S4b. Top: ^1^H NMR spectrum of **4** (1.6 × 10^−2^M) in the presence of 10-fold excess of lead(II) perchlorate; bottom comparison of the spectral pattern of proton signals in free crown 4 and its spectrum in the presence of 10-fold excess lead(II) perchlorate (DMSO-d_6_); Figure S5. UV-Vis titration trace and color change for **4** (c_4_ = 3.14 × 10^−5^ M) and zinc(II) perchlorate (c_Zn_ = 0–1.76 × 10^−3^ M) in acetonitrile. Bottom: color changes of the solutions; Figure S6. Interferences from several metal perchlorates, used in 10-fold excess, on spectrophotometric response towards systems containing equimolar amount of lead(II) perchlorate for (a) **1** (2.54 × 10^−5^ M) at 605 nm, (b) **2** (2.73 × 10^−5^ M) at 610 nm, (c) **3** (2.08 × 10^−5^ M) at 610 nm and (d) **4** (2.63 × 10^−5^ M) at 605 nm in acetonitrile (Mix—mixture of metal perchlorates without copper). (e) interferences from several metal perchlorates, used in 10-fold excess, on spectrophotometric response towards systems containing equimolar amount of lead(II) perchlorate for **3** (2.13 × 10^−5^ M) at 608 nm at equimolar (to crown) amount of lead(II) perchlorate in acetonitrile:water (9:1, *v/v*) solution at pH 5; Figure S7. (a) The relationship A = f([Pb(II)]) at 610 nm and (b) linear range of response towards lead(II) perchlorate for **3** in acetonitrile; Figure S8. (a) The relationship A = f([Pb(II)]) at 605 nm and (b) linear range of response towards lead(II) perchlorate for **4** in acetonitrile; FigureS9. The relationship (a) A = f ([Pb(II)]) with linear range of response 5.00 × 10^−7^–1.20 × 10^−5^ M (b) A = f (log([Pb(II)]) with linear range of response 5.00 × 10^−6^–1.15 × 10^−4^ M for 3 (c_3_ = 2.13 × 10^−5^ M) in acetonitrile:water (9:1, *v/v*) solution at pH 5 at 608 nm; Figure S10. RP18-TLC chromatograms of crowns **1**–**4** and standard substances a–e; **4**. Ionic species detected in ESI mass spectra of lead(II) complex of crown **1**: Figure S11a. Theoretically calculated and experimental isotope pattern of peak m/z 1950 in lead(II) complex of **1**; Figure S11b. Theoretically calculated and experimental isotope pattern of peak m/z 1745 in lead(II) complex of **1**; Figure S11c. Theoretically calculated and experimental isotope pattern of peak m/z 1644 in lead(II) complex of 1; Figure S11d. Theoretically calculated and experimental isotope pattern of peak m/z 1267 in lead(II) complex of 1; Figure S11e. Theoretically calculated and experimental isotope pattern of peak m/z 584 in lead(II) complex of **1**; **4**. Ionic species detected in ESI mass spectra of lead(II) complex of crown **2**: Figure S12a. Theoretically calculated and experimental isotope pattern of peak m/z 2082 in lead(II) complex of 2; Figure S12b. Theoretically calculated and experimental isotope pattern of peak m/z 1876 in lead(II) complex of **2**; Figure S12c. Theoretically calculated and experimental isotope pattern of peak m/z 1776 in lead(II) complex of 2; Figure S12d. Theoretically calculated and experimental isotope pattern of peak m/z 1335 in lead(II) complex of **2**; Figure S12e. Theoretically calculated and experimental isotope pattern of peak m/z 628 in lead(II) complex of **2**; **5**. Ionic species detected in ESI mass spectra of lead (II) complex of crown **3**: Figure S13a. Theoretically calculated and experimental isotope pattern of peak m/z 1944 in lead(II) complex of **3**; Figure S13b. Theoretically calculated and experimental isotope pattern of peak m/z 1636 in lead(II) complex of **3**; Figure S13c. Theoretically calculated and experimental isotope pattern of peak m/z 1263 in lead(II) complex of **3**; Figure S13d. Theoretically calculated and experimental isotope pattern of peak m/z 957 in lead(II) complex of **3**; Figure S13e. Theoretically calculated and experimental isotope pattern of peak m/z 582 in lead(II) complex of **3**; **6**. Ionic species detected in ESI mass spectra of lead (II) complex of crown **4**: Figure S14a. Theoretically calculated and experimental isotope pattern of peak m/z 2154 in lead(II) complex of **4**; Figure S14b. Theoretically calculated and experimental isotope pattern of peak m/z 1948 in lead(II) complex of **4**; Figure S14c. Theoretically calculated and experimental isotope pattern of peak m/z 1848 in lead(II) complex of **4**; Figure S14d. Theoretically calculated and experimental isotope pattern of peak m/z 1403 in lead(II) complex of **4**; Figure S14e. Theoretically calculated and experimental isotope pattern of peak m/z 652 in lead(II) complex of **4**; **7**. X-ray structure of **3**: Crystallographic details; Figure S15. Molecular view of **3** showing atom labeling scheme; Table S2. Crystal data and structure refinement details for **3**; Figure S16. Crystal packing in **3**. Some molecules form stacking layers in parallel, but some pack almost perpendicular to them making complex space-filling pattern. Molecules are coloured by symmetry operation type, hydrogen atoms omitted; Experimental details; Computing details.

## Appendix A

CCDC 2081633 contains the supplementary crystallographic data for 3. These data can be obtained free of charge via http://www.ccdc.cam.ac.uk/conts/retrieving.html (accessed on 12 October 2021), or from the Cambridge Crystallographic Data Centre, 12 Union Road, Cambridge CB2 1EZ, UK; fax: (+44) 1223-336-033; or e-mail: deposit@ccdc.cam.ac.uk.
