# Supplementary material for: Novel Diazocrowns with Pyrrole Residue as Lead(II)Colorimetric Probes
Source: Materials (Basel). 2021 Nov 26;14(23):7239. doi: 10.3390/ma14237239 (PMC8658487; doi:10.3390/ma14237239)
Supplement: Supplementary file 1 [file materials-14-07239-s001.zip › materials-1448153-SM.pdf]

materials

Supplementary Materials

Novel Diazocrowns with Pyrrole Residue as Lead(II) Colorimetric Probes

Błażej Galiński [1], Elżbieta Luboch [1], Jarosław Chojnacki [2] and Ewa Wagner-Wysiecka [1,*]

[1] Department of Chemistry and Technology of Functional Materials, Faculty of Chemistry, Gdańsk University of Technology, Narutowicza Street 11/12, 80-233 Gdańsk, Poland; blazej.galinski@pg.edu.pl (B.G.); elzlub-oc@pg.edu.pl (E.L.)
[2] Department of Inorganic Chemistry, Faculty of Chemistry, Gdańsk University of Technology, Narutowicza Street 11/12, 80-233 Gdańsk, Poland; jaroslaw.chojnacki@pg.edu.pl
* Correspondence: ewa.wagner-wysiecka@pg.edu.pl

Citation: Galiński, B.; Luboch, E.; Chojnacki, J.; Wagner-Wysiecka, E. Novel Diazocrowns with Pyrrole Residue as Lead(II) Colori-metric Probes. *Materials* 2021, 14, 7239. https://doi.org/10.3390/ma14237239

Academic Editor: Anastasios J. Tasiopoulos

Received: 19 October 2021
Accepted: 22 November 2021
Published: date

Publisher's Note: MDPI stays neutral with regard to jurisdictional claims in published maps and institutional affiliations.

Copyright: © 2021 by the authors. Licensee MDPI, Basel, Switzerland. This article is an open access article distributed under the terms and conditions of the Creative Commons Attribution (CC BY) license (http://creativecommons.org/licenses/by/4.0/).

1. Spectra of Compounds 3 and 4 and Lead(II) Complexes

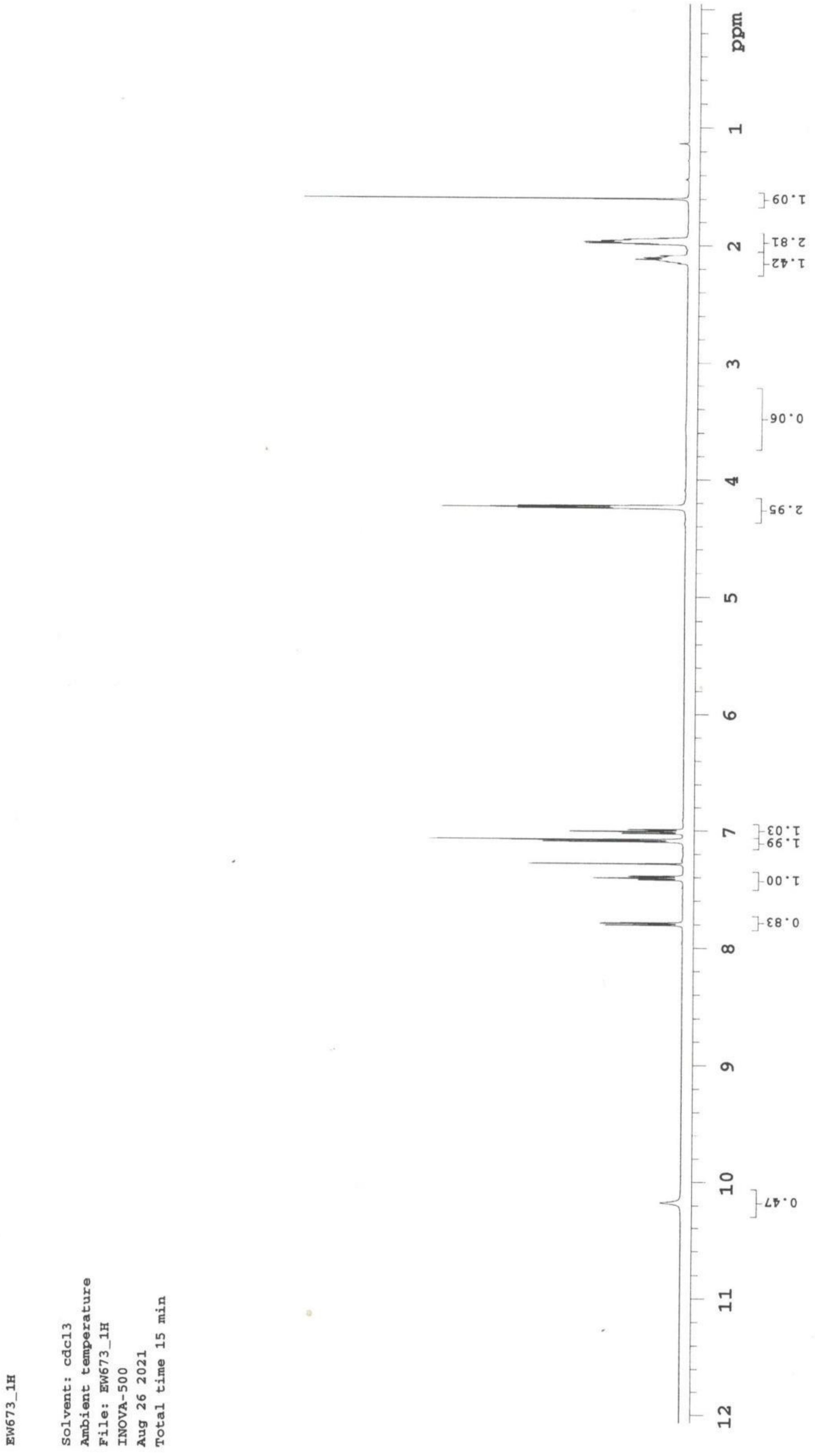

Figure S1a. ^{1}H NMR of **3** (d-chloroform).

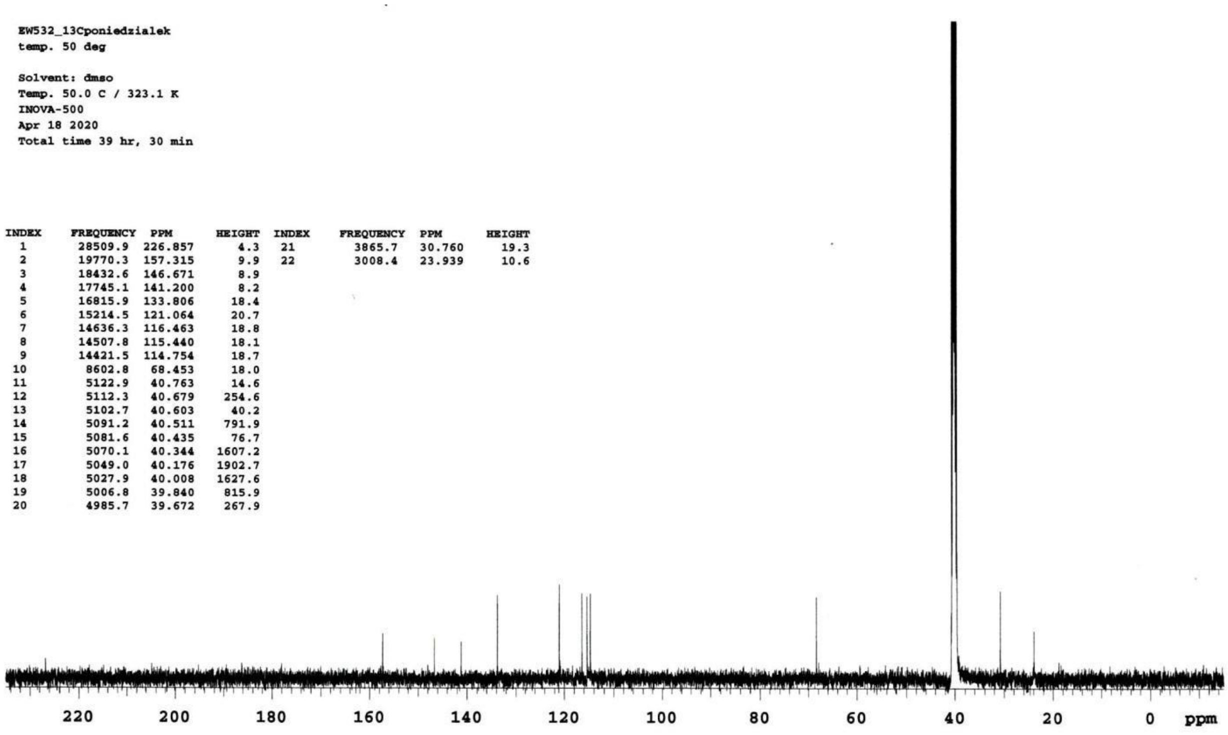

Figure S1b. ^{13}C NMR of 3 (DMSO-d6).

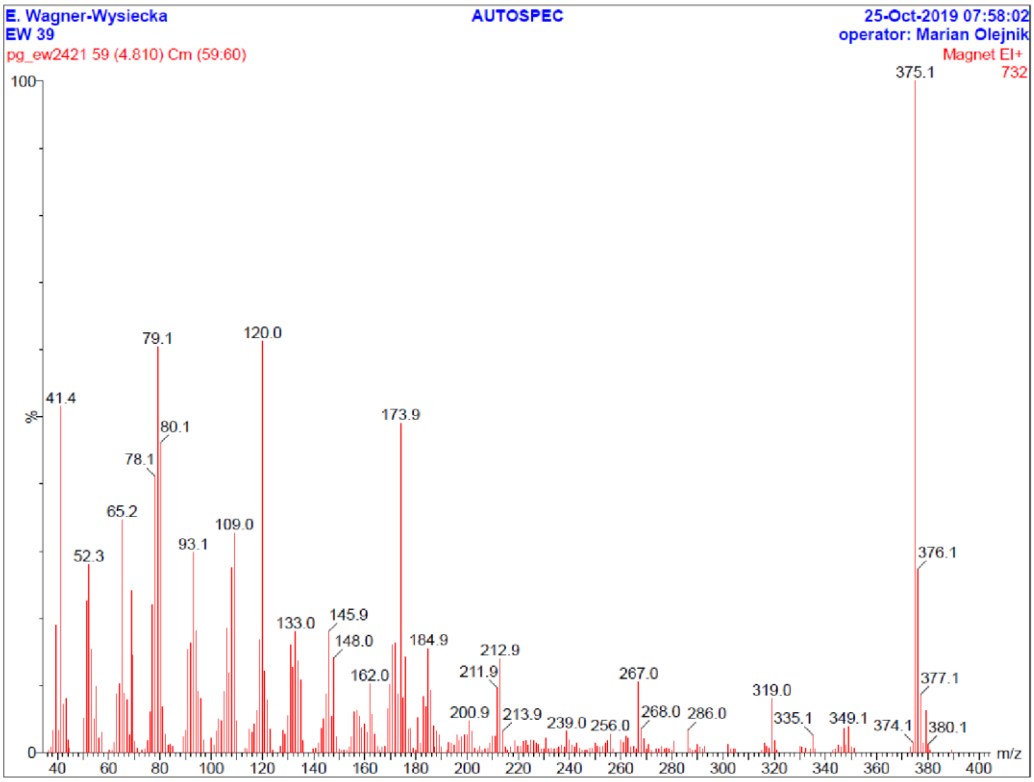

E. Wagner-Wysiecka AUTOSPEC 25-Oct-2019 07:58:02
EW 39 operator: Marian Olejnik
pg_ew2421 59 (4.810) Cm (59:60) Magnet EI+

No	Mass	Inten	%BPI	%TIC
1:	37.4	6.00e0	0.82	0.05
2:	38.4	2.40e1	3.28	0.21
3:	39.4	1.39e2	18.99	1.21
4:	40.4	2.40e1	3.28	0.21
5:	41.4	3.77e2	51.50	3.28
6:	42.4	5.30e1	7.24	0.46
7:	43.4	5.90e1	8.06	0.51
8:	44.3	1.40e1	1.91	0.12
9:	50.3	3.70e1	5.05	0.32
10:	51.3	1.65e2	22.54	1.43
11:	52.3	2.05e2	28.01	1.78
12:	53.3	1.12e2	15.30	0.97
13:	54.3	3.60e1	4.92	0.31
14:	55.3	7.20e1	9.84	0.63
15:	56.3	1.60e1	2.19	0.14
16:	57.3	2.20e1	3.01	0.19
17:	62.2	1.10e1	1.50	0.10
18:	63.2	6.40e1	8.74	0.56
19:	64.2	7.50e1	10.25	0.65
20:	65.2	2.53e2	34.56	2.20
21:	66.2	6.50e1	8.88	0.56
22:	67.2	5.80e1	7.92	0.50
23:	68.2	1.90e1	2.60	0.17
24:	69.1	1.76e2	24.04	1.53
25:	69.2	1.06e2	14.48	0.92
26:	70.2	1.20e1	1.64	0.10
27:	75.1	1.30e1	1.78	0.11
28:	76.1	4.40e1	6.01	0.38
29:	77.1	1.61e2	21.99	1.40
30:	78.1	3.01e2	41.12	2.62
31:	79.1	4.42e2	60.38	3.84
32:	80.1	3.37e2	46.04	2.93
33:	81.1	4.90e1	6.69	0.43
34:	82.1	2.00e1	2.73	0.17
35:	83.1	8.00e0	1.09	0.07
36:	84.1	9.00e0	1.23	0.08
37:	85.1	7.00e0	0.96	0.06
38:	89.1	1.70e1	2.32	0.15
39:	90.1	2.40e1	3.28	0.21
40:	91.1	1.12e2	15.30	0.97
41:	92.1	1.19e2	16.26	1.03
42:	93.1	2.18e2	29.78	1.89
43:	94.1	1.33e2	18.17	1.16
44:	95.1	6.70e1	9.15	0.58
45:	96.1	5.90e1	8.06	0.51
46:	97.1	1.40e1	1.91	0.12
47:	100.0	1.60e1	2.19	0.14
48:	101.0	8.00e0	1.09	0.07
49:	102.0	2.30e1	3.14	0.20
50:	103.0	3.60e1	4.92	0.31
51:	104.0	3.40e1	4.64	0.30
52:	105.0	6.70e1	9.15	0.58
53:	106.0	1.36e2	18.58	1.18
54:	107.0	8.70e1	11.89	0.76
55:	108.0	2.02e2	27.60	1.76
56:	109.0	2.39e2	32.65	2.08
57:	110.0	3.40e1	4.64	0.30
58:	115.0	2.50e1	3.42	0.22
59:	116.0	2.20e1	3.01	0.19
60:	117.0	3.10e1	4.23	0.27
61:	118.0	4.50e1	6.15	0.39
62:	119.0	1.23e2	16.80	1.07
63:	120.0	4.48e2	61.20	3.89
64:	121.0	8.90e1	12.16	0.77
65:	122.0	5.80e1	7.92	0.50
66:	123.0	2.50e1	3.42	0.22
67:	127.0	7.00e0	0.96	0.06
68:	128.0	2.40e1	3.28	0.21
69:	129.0	2.00e1	2.73	0.17
70:	130.0	4.00e1	5.46	0.35
71:	130.9	1.17e2	15.98	1.02
72:	132.0	9.30e1	12.70	0.81
73:	133.0	1.32e2	18.03	1.15
74:	134.0	1.00e2	13.66	0.87
75:	135.0	7.90e1	10.79	0.69
76:	136.0	1.30e1	1.78	0.11
77:	141.9	1.00e1	1.37	0.09
78:	142.9	2.60e1	3.55	0.23
79:	143.9	3.60e1	4.92	0.31
80:	144.9	6.40e1	8.74	0.56
81:	145.9	1.32e2	18.03	1.15
82:	147.0	3.90e1	5.33	0.34
83:	148.0	1.03e2	14.07	0.89
84:	149.0	1.70e1	2.32	0.15
85:	153.9	6.00e0	0.82	0.05
86:	154.9	1.70e1	2.32	0.15
87:	155.9	4.40e1	6.01	0.38
88:	156.9	4.50e1	6.15	0.39
89:	157.9	3.90e1	5.33	0.34
90:	158.9	2.70e1	3.69	0.23
91:	159.9	3.10e1	4.23	0.27
92:	161.0	2.20e1	3.01	0.19
93:	162.0	7.50e1	10.25	0.65
94:	163.0	4.10e1	5.60	0.36
95:	164.0	2.00e1	2.73	0.17
96:	164.9	7.00e0	0.96	0.06
97:	166.9	6.00e0	0.82	0.05
98:	167.9	7.00e0	0.96	0.06
99:	168.9	4.70e1	6.42	0.41
100:	169.9	7.40e1	10.11	0.64
101:	170.9	1.17e2	15.98	1.02
102:	171.9	1.20e2	16.39	1.04
103:	172.9	6.40e1	8.74	0.56
104:	173.9	3.59e2	49.04	3.12
105:	174.9	6.00e1	8.20	0.52
106:	176.0	1.04e2	14.21	0.90
107:	177.0	2.50e1	3.42	0.22
108:	178.0	2.60e1	3.55	0.23
109:	180.8	3.80e1	5.19	0.33
110:	181.9	1.00e1	1.37	0.09
111:	182.9	6.10e1	8.33	0.53
112:	183.9	4.90e1	6.69	0.43
113:	184.9	1.13e2	15.44	0.98
114:	185.9	6.80e1	9.29	0.59
115:	186.9	2.90e1	3.96	0.25
116:	187.4	6.00e0	0.82	0.05
117:	187.9	2.30e1	3.14	0.20
118:	188.9	1.90e1	2.60	0.17
119:	189.9	6.00e0	0.82	0.05
120:	192.9	1.10e1	1.50	0.10
121:	193.9	1.00e1	1.37	0.09
122:	194.9	8.00e0	1.09	0.07
123:	195.9	1.90e1	2.60	0.17
124:	196.9	1.30e1	1.78	0.11
125:	197.9	1.70e1	2.32	0.15
126:	198.9	1.90e1	2.60	0.17
127:	199.9	1.90e1	2.60	0.17
128:	200.9	3.40e1	4.64	0.30
129:	201.9	2.30e1	3.14	0.20
130:	204.9	7.00e0	0.96	0.06
131:	208.9	1.00e1	1.37	0.09
132:	209.9	1.80e1	2.46	0.16
133:	210.9	1.80e1	2.46	0.16
134:	211.9	7.00e1	9.56	0.61
135:	212.9	1.02e2	13.93	0.89
136:	213.9	2.30e1	3.14	0.20
137:	214.9	6.00e0	0.82	0.05
138:	216.9	6.00e0	0.82	0.05
139:	218.8	1.30e1	1.78	0.11
140:	219.9	7.00e0	0.96	0.06
141:	220.9	7.00e0	0.96	0.06
142:	221.9	1.20e1	1.64	0.10
143:	222.9	1.30e1	1.78	0.11
144:	223.9	8.00e0	1.09	0.07
145:	224.9	1.40e1	1.91	0.12
146:	225.9	1.30e1	1.78	0.11
147:	226.9	1.00e1	1.37	0.09
148:	228.0	9.00e0	1.23	0.08
149:	230.8	1.60e1	2.19	0.14
150:	235.9	6.00e0	0.82	0.05
151:	236.9	8.00e0	1.09	0.07
152:	237.9	6.00e0	0.82	0.05
153:	239.0	2.30e1	3.14	0.20
154:	240.0	1.40e1	1.91	0.12
155:	241.0	8.00e0	1.09	0.07
156:	242.0	6.00e0	0.82	0.05
157:	242.9	1.00e1	1.37	0.09
158:	250.0	1.00e1	1.37	0.09
159:	251.0	7.00e0	0.96	0.06
160:	252.0	6.00e0	0.82	0.05
161:	253.0	7.00e0	0.96	0.06
162:	254.0	1.00e1	1.37	0.09
163:	255.0	1.30e1	1.78	0.11
164:	256.0	2.00e1	2.73	0.17
165:	259.9	1.40e1	1.91	0.12
166:	260.9	1.10e1	1.50	0.10
167:	262.0	1.80e1	2.46	0.16
168:	263.0	1.60e1	2.19	0.14
169:	264.0	6.00e0	0.82	0.05
170:	265.0	7.00e0	0.96	0.06
171:	267.0	7.70e1	10.52	0.67
172:	268.0	2.60e1	3.55	0.23
173:	269.0	1.50e1	2.05	0.13
174:	271.0	9.00e0	1.23	0.08
175:	276.0	7.00e0	0.96	0.06
176:	280.9	1.20e1	1.64	0.10
177:	286.0	2.40e1	3.28	0.21
178:	290.0	9.00e0	1.23	0.08
179:	291.0	6.00e0	0.82	0.05
180:	292.9	7.00e0	0.96	0.06
181:	302.0	9.00e0	1.23	0.08
182:	316.0	1.00e1	1.37	0.09
183:	317.0	7.00e0	0.96	0.06
184:	319.0	5.90e1	8.06	0.51
185:	320.0	1.30e1	1.78	0.11
186:	330.0	7.00e0	0.96	0.06
187:	331.0	6.00e0	0.82	0.05
188:	335.1	1.90e1	2.60	0.17
189:	345.1	8.00e0	1.09	0.07
190:	346.1	6.00e0	0.82	0.05
191:	347.1	2.60e1	3.55	0.23
192:	348.1	8.00e0	1.09	0.07
193:	349.1	2.80e1	3.83	0.24
194:	350.1	6.00e0	0.82	0.05
195:	373.1	6.00e0	0.82	0.05
196:	374.1	1.10e1	1.50	0.10
197:	375.1	7.32e2	100.00	6.36
198:	376.1	2.00e2	27.32	1.74
199:	377.1	6.30e1	8.61	0.55
200:	378.1	1.00e1	1.37	0.09
201:	379.1	4.50e1	6.15	0.39
202:	380.1	9.00e0	1.23	0.08

Figure S1c. LRMS (EI) of **3**.

Elemental Composition Report Page 1

Single Mass Analysis

Tolerance = 15.0 PPM / DBE: min = -1.5, max = 50.0
Selected filters: None

Monoisotopic Mass, Odd and Even Electron Ions
23 formula(e) evaluated with 1 results within limits (up to 50 best isotopic matches for each mass)
Elements Used:
C: 0-60 H: 0-100 N: 5-5 O: 0-4

E. Wagner-Wysiecka AUTOSPEC 28-Oct-2019 12:59:50
EW 39 Operator: Marian Olejnik
pg_ew2421h 264 (10.068) Cm (260:282) Voltage EI+
1.54e3

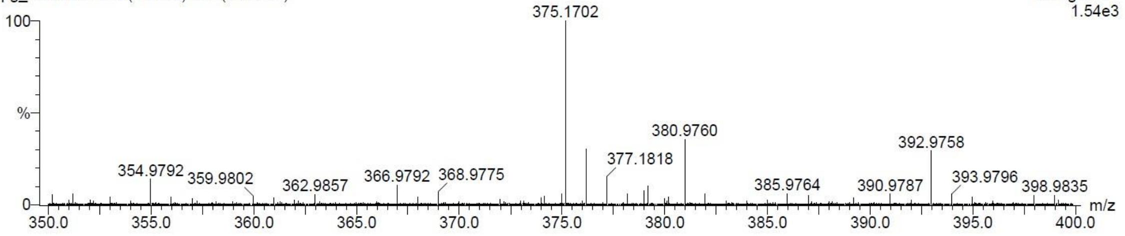

Minimum:				-1.5		
Maximum:		5.0	15.0	50.0		
Mass	Calc. Mass	mDa	PPM	DBE	i-FIT	Formula
375.1702	375.1695	0.7	1.9	14.0	72.2	C21 H21 N5 O2

Figure S1d. HRMS (EI) of **3**.

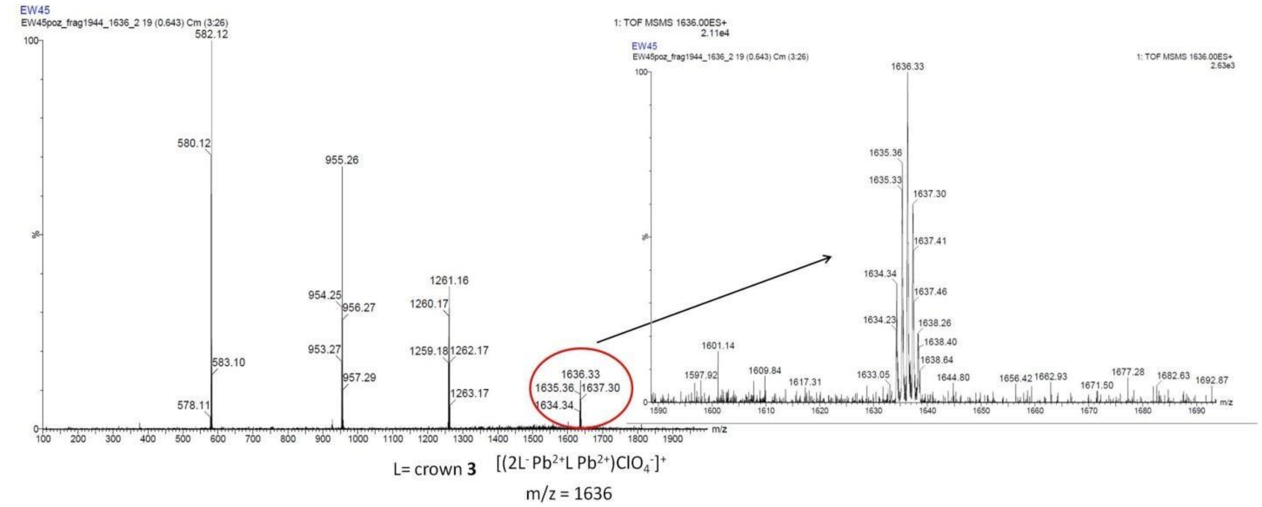

Figure S1e. Fragmentation pattern for lead(II) complex (ESI—positive ions mode) of **3** and part of mass spectrum presenting isotopic lead(II) peaks with peak of the highest intensity m/z 1636.

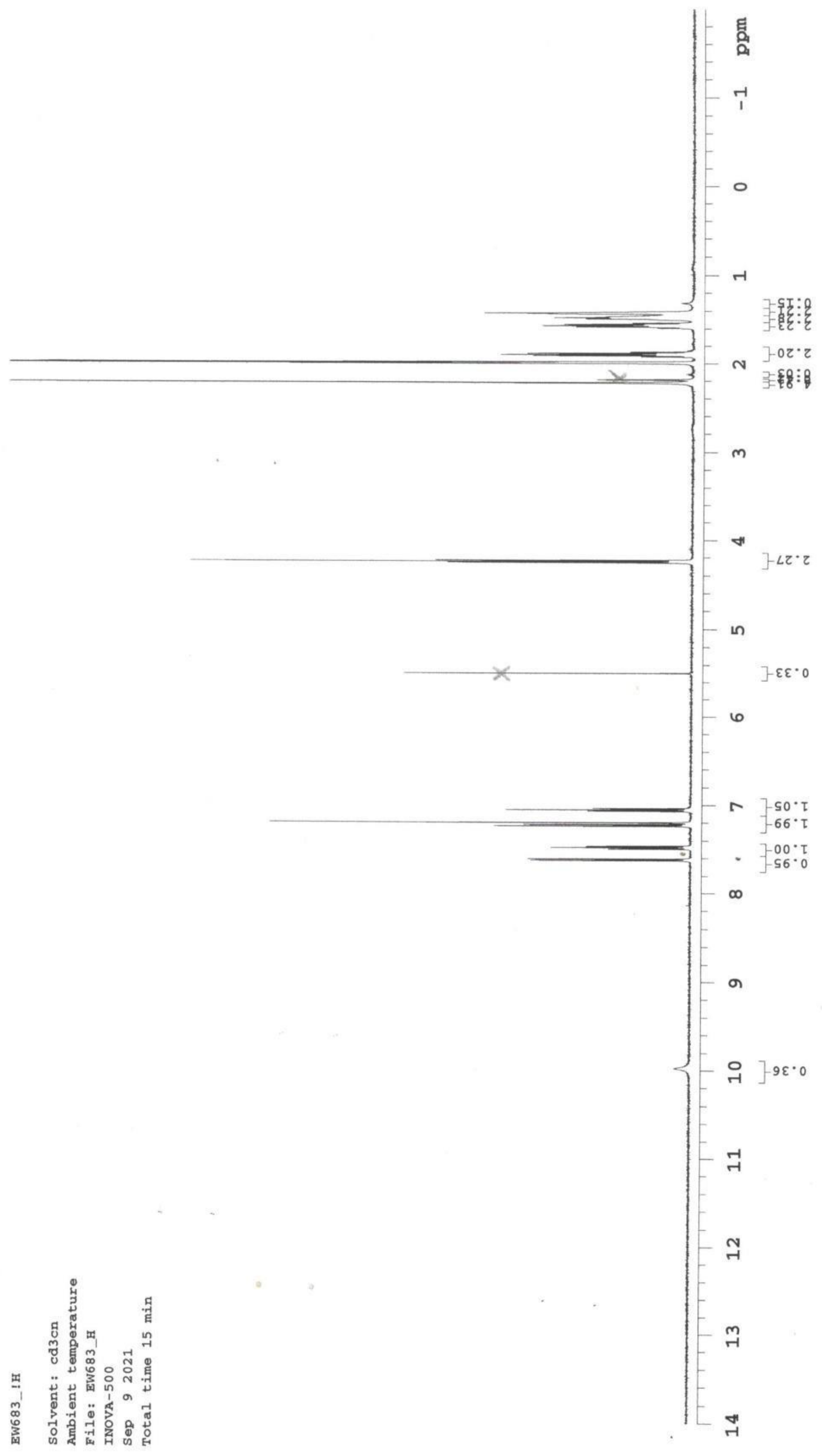

x—solvent residual impurities: 5.45 ppm—dichloromethane, 2.04—acetone

Figure S2a. ^{1}H NMR of **4** (acetonitrile-d3).

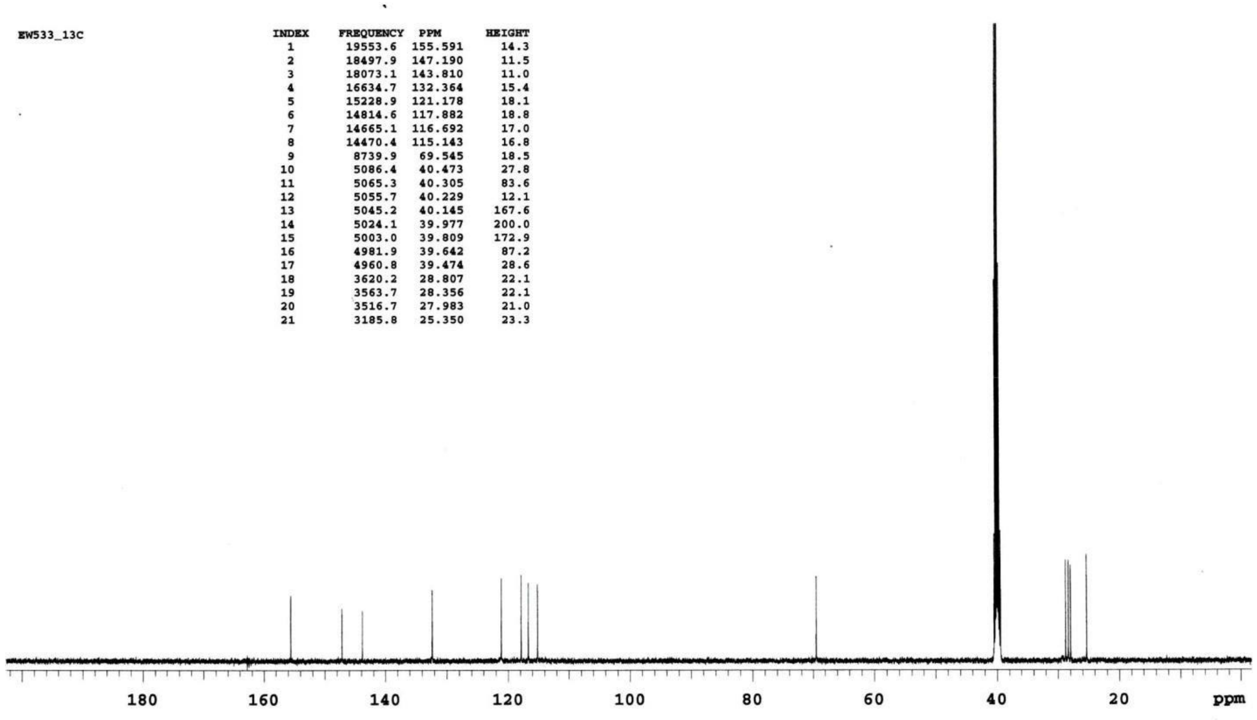

Figure S2b. ^{13}C NMR of **4** (DMSO-d_6).

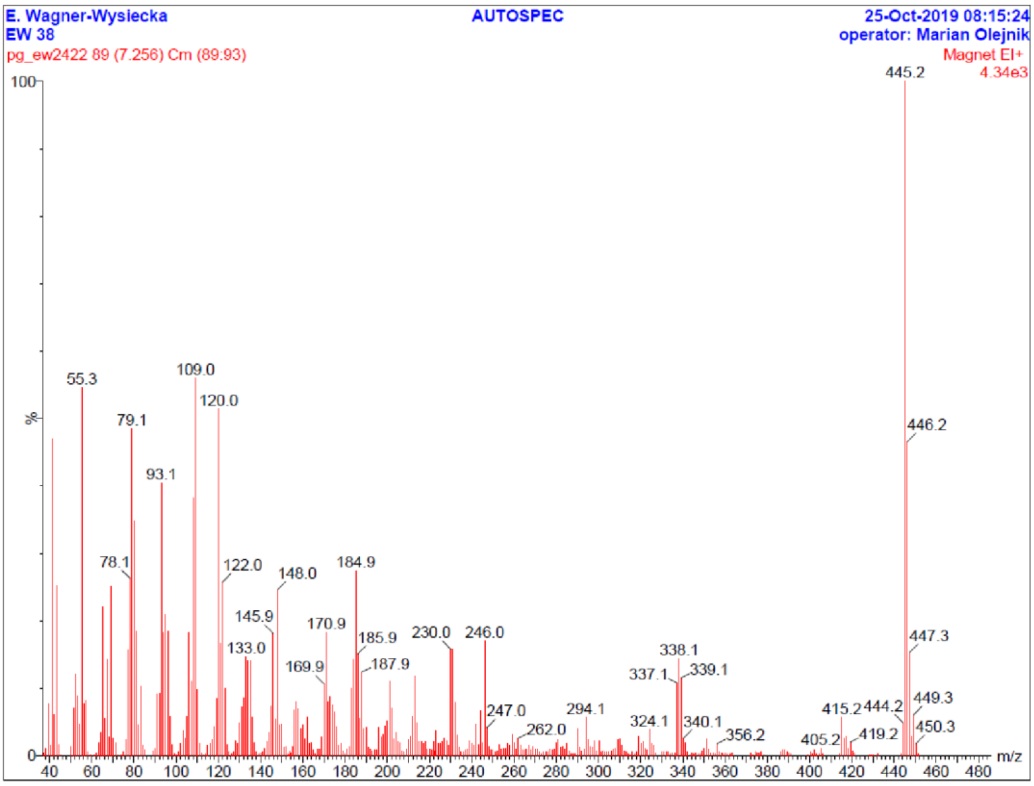

E. Wagner-Wysiecka AUTOSPEC 25-Oct-2019 08:15:24
EW 38 operator: Marian Olejnik
pg_ew2422 89 (7.256) Cm (89:93) Magnet EI+

No	Mass	Inten	%BPI	%TIC	No	Mass	Inten	%BPI	%TIC	No	Mass	Inten	%BPI	%TIC	No	Mass	Inten	%BPI	%TIC
1:	39.4	3.35e2	7.71	0.44	52:	115.0	6.70e1	1.54	0.09	103:	183.9	6.18e2	14.22	0.80	154:	253.0	6.90e1	1.59	0.09
2:	40.4	6.30e1	1.45	0.08	53:	116.0	7.40e1	1.70	0.10	104:	184.9	1.19e3	27.41	1.55	155:	257.0	7.40e1	1.70	0.10
3:	41.4	2.04e3	46.90	2.65	54:	117.0	1.01e2	2.32	0.13	105:	185.9	6.53e2	15.03	0.85	156:	258.0	6.40e1	1.47	0.08
4:	42.4	2.61e2	6.01	0.34	55:	118.0	1.84e2	4.23	0.24	106:	186.9	2.43e2	5.59	0.32	157:	259.0	1.39e2	3.20	0.18
5:	43.4	1.10e3	25.22	1.42	56:	119.0	3.67e2	8.45	0.48	107:	187.9	5.37e2	12.36	0.70	158:	260.0	8.30e1	1.91	0.11
6:	44.4	7.20e1	1.66	0.09	57:	120.0	2.23e3	51.39	2.90	108:	188.9	1.74e2	4.00	0.23	159:	262.0	1.12e2	2.58	0.15
7:	50.3	5.80e1	1.33	0.08	58:	121.0	7.21e2	16.59	0.94	109:	190.0	1.79e2	4.12	0.23	160:	263.0	6.50e1	1.50	0.08
8:	51.3	3.05e2	7.02	0.40	59:	122.0	1.12e3	25.75	1.45	110:	191.0	5.60e1	1.29	0.07	161:	267.0	6.40e1	1.47	0.08
9:	52.3	5.24e2	12.06	0.68	60:	123.0	4.32e2	9.94	0.56	111:	195.9	1.78e2	4.10	0.23	162:	269.0	5.80e1	1.33	0.08
10:	53.3	3.86e2	8.88	0.50	61:	124.0	7.00e1	1.61	0.09	112:	196.9	1.19e2	2.74	0.15	163:	280.0	8.60e1	1.98	0.11
11:	54.3	2.02e2	4.65	0.26	62:	128.0	9.40e1	2.16	0.12	113:	197.9	1.38e2	3.18	0.18	164:	281.0	1.04e2	2.39	0.14
12:	55.3	2.37e3	54.61	3.08	63:	129.0	7.50e1	1.73	0.10	114:	198.9	1.94e2	4.46	0.25	165:	282.0	5.40e1	1.24	0.07
13:	56.3	3.36e2	7.73	0.44	64:	130.0	2.13e2	4.90	0.28	115:	199.9	2.22e2	5.11	0.29	166:	283.0	5.80e1	1.33	0.08
14:	57.3	3.56e2	8.19	0.46	65:	131.0	3.13e2	7.20	0.41	116:	200.9	4.81e2	11.07	0.63	167:	285.1	7.50e1	1.73	0.10
15:	63.2	8.50e1	1.96	0.11	66:	132.0	3.73e2	8.58	0.48	117:	201.9	3.04e2	7.00	0.40	168:	290.0	1.75e2	4.03	0.23
16:	64.2	1.51e2	3.48	0.20	67:	133.0	6.37e2	14.66	0.83	118:	203.0	1.06e2	2.44	0.14	169:	291.0	5.40e1	1.24	0.07
17:	65.2	9.54e2	21.96	1.24	68:	134.0	6.13e2	14.11	0.80	119:	204.0	1.50e2	3.45	0.19	170:	294.1	2.47e2	5.68	0.32
18:	66.2	2.42e2	5.57	0.31	69:	135.0	6.15e2	14.15	0.80	120:	205.0	8.80e1	2.03	0.11	171:	295.1	1.04e2	2.39	0.14
19:	67.2	6.18e2	14.22	0.80	70:	136.0	2.44e2	5.62	0.32	121:	206.0	8.10e1	1.86	0.11	172:	296.1	6.20e1	1.43	0.08
20:	68.2	1.22e2	2.81	0.16	71:	137.0	8.20e1	1.89	0.11	122:	209.9	1.04e2	2.39	0.14	173:	297.1	5.60e1	1.29	0.07
21:	69.1	1.22e2	2.81	0.16	72:	142.9	8.90e1	2.05	0.12	123:	210.9	1.23e2	2.83	0.16	174:	298.1	9.70e1	2.23	0.13
22:	69.2	1.09e3	25.02	1.41	73:	143.9	1.51e2	3.48	0.20	124:	211.9	2.50e2	5.75	0.32	175:	300.1	9.80e1	2.26	0.13
23:	70.2	1.16e2	2.67	0.15	74:	145.0	3.16e2	7.27	0.41	125:	212.9	5.13e2	11.81	0.67	176:	309.1	1.00e2	2.30	0.13
24:	71.2	8.60e1	1.98	0.11	75:	145.9	7.87e2	18.11	1.02	126:	213.9	2.11e2	4.86	0.27	177:	310.1	1.07e2	2.46	0.14
25:	76.1	1.04e2	2.39	0.14	76:	147.0	2.35e2	5.41	0.31	127:	215.0	8.90e1	2.05	0.12	178:	311.1	6.50e1	1.50	0.08
26:	77.1	6.78e2	15.60	0.88	77:	148.0	1.07e3	24.56	1.39	128:	216.0	8.90e1	2.05	0.12	179:	319.0	1.28e2	2.95	0.17
27:	78.1	1.14e3	26.26	1.48	78:	149.0	2.01e2	4.63	0.26	129:	217.0	8.00e1	1.84	0.10	180:	320.0	7.80e1	1.80	0.10
28:	79.1	2.11e3	48.45	2.74	79:	150.0	2.05e2	4.72	0.27	130:	218.0	9.00e1	2.07	0.12	181:	321.1	9.20e1	2.12	0.12
29:	80.1	1.51e3	34.71	1.96	80:	152.0	6.20e1	1.43	0.08	131:	221.9	9.10e1	2.09	0.12	182:	324.1	1.70e2	3.91	0.22
30:	81.1	7.98e2	18.37	1.04	81:	154.9	6.20e1	1.43	0.08	132:	222.9	1.61e2	3.71	0.21	183:	325.1	7.90e1	1.82	0.10
31:	82.1	1.95e2	4.49	0.25	82:	155.9	2.91e2	6.70	0.38	133:	224.0	7.70e1	1.77	0.10	184:	326.1	6.50e1	1.50	0.08
32:	83.1	4.47e2	10.29	0.58	83:	156.9	3.46e2	7.96	0.45	134:	224.9	7.60e1	1.75	0.10	185:	337.1	4.68e2	10.77	0.61
33:	84.1	6.40e1	1.47	0.08	84:	157.9	3.01e2	6.93	0.39	135:	225.9	9.10e1	2.09	0.12	186:	338.1	6.28e2	14.45	0.82
34:	91.1	3.99e2	9.18	0.52	85:	158.9	1.73e2	3.98	0.22	136:	226.9	1.11e2	2.55	0.14	187:	339.1	5.00e2	11.51	0.65
35:	92.1	4.04e2	9.30	0.53	86:	159.9	2.01e2	4.63	0.26	137:	228.0	9.80e1	2.26	0.13	188:	340.1	1.11e2	2.55	0.14
36:	93.1	1.76e3	40.48	2.29	87:	161.0	1.05e2	2.42	0.14	138:	229.0	6.70e1	1.54	0.09	189:	341.2	8.20e1	1.89	0.11
37:	94.1	7.94e2	18.27	1.03	88:	162.0	2.46e2	5.66	0.32	139:	230.0	6.84e2	15.74	0.89	190:	351.1	1.09e2	2.51	0.14
38:	95.1	9.09e2	20.92	1.18	89:	163.0	7.60e1	1.75	0.10	140:	231.0	6.83e2	15.72	0.89	191:	356.2	7.70e1	1.77	0.10
39:	96.1	8.03e2	18.48	1.04	90:	164.0	8.60e1	1.98	0.11	141:	232.0	3.41e2	7.85	0.44	192:	415.2	2.47e2	5.68	0.32
40:	97.1	2.54e2	5.85	0.33	91:	168.9	1.21e2	2.78	0.16	142:	233.0	1.34e2	3.08	0.17	193:	416.2	1.14e2	2.62	0.15
41:	98.1	7.10e1	1.63	0.09	92:	169.9	4.62e2	10.63	0.60	143:	234.0	5.50e1	1.27	0.07	194:	417.2	1.25e2	2.88	0.16
42:	102.0	7.60e1	1.75	0.10	93:	170.9	7.93e2	18.25	1.03	144:	239.0	9.60e1	2.21	0.12	195:	419.2	8.60e1	1.98	0.11
43:	103.0	1.61e2	3.71	0.21	94:	171.9	3.50e2	8.06	0.45	145:	240.0	7.90e1	1.82	0.10	196:	444.2	2.11e2	4.86	0.27
44:	104.0	1.16e2	2.67	0.15	95:	172.9	3.76e2	8.65	0.49	146:	241.0	6.60e1	1.52	0.09	197:	445.2	4.34e3	100.00	5.65
45:	105.0	2.53e2	5.82	0.33	96:	173.9	3.26e2	7.50	0.42	147:	242.0	2.05e2	4.72	0.27	198:	446.2	2.02e3	46.54	2.63
46:	106.0	7.95e2	18.30	1.03	97:	174.9	2.79e2	6.42	0.36	148:	243.0	7.10e1	1.63	0.09	199:	447.3	6.62e2	15.24	0.86
47:	107.0	4.82e2	11.09	0.63	98:	176.0	1.86e2	4.28	0.24	149:	244.0	2.90e2	6.67	0.38	200:	448.3	1.26e2	2.90	0.16
48:	108.0	1.66e3	38.30	2.16	99:	177.0	6.60e1	1.52	0.09	150:	245.0	8.50e1	1.96	0.11	201:	449.3	2.63e2	6.05	0.34
49:	109.0	2.43e3	55.93	3.16	100:	178.0	7.50e1	1.73	0.10	151:	246.0	7.40e2	17.03	0.96	202:	450.3	7.70e1	1.77	0.10
50:	110.0	4.28e2	9.85	0.56	101:	181.9	6.00e1	1.38	0.08	152:	247.0	1.78e2	4.10	0.23					
51:	111.0	7.60e1	1.75	0.10	102:	182.9	4.31e2	9.92	0.56	153:	248.0	6.10e1	1.40	0.08					

Figure S2c. LRMS (EI) of **4**.

Elemental Composition Report

Page 1

Single Mass Analysis

Tolerance = 15.0 PPM / DBE: min = -1.5, max = 50.0
Selected filters: None

Monoisotopic Mass, Odd and Even Electron Ions
28 formula(e) evaluated with 1 results within limits (up to 50 best isotopic matches for each mass)
Elements Used:
C: 0-60 H: 0-100 N: 5-5 O: 0-4

E. Wagner-Wysiecka AUTOSPEC 28-Oct-2019 13:16:34
EW 38 Operator: Marian Olejnik
pg_ew2422h 131 (4.997) Cm (131:146) Voltage EI+
3.60e3

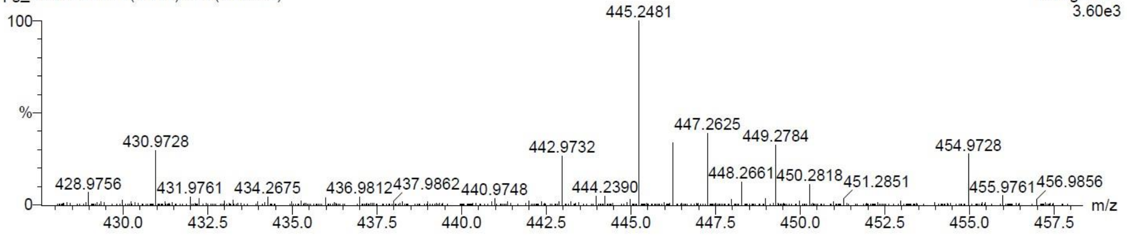


```
Minimum:                                      -1.5
Maximum:                   5.0        15.0    50.0

Mass       Calc. Mass     mDa        PPM     DBE       i-FIT      Formula

445.2481   445.2478       0.3        0.7     14.0      523.4      C26  H31  N5  O2
```

Figure S2d. HRMS (EI) of **4**.

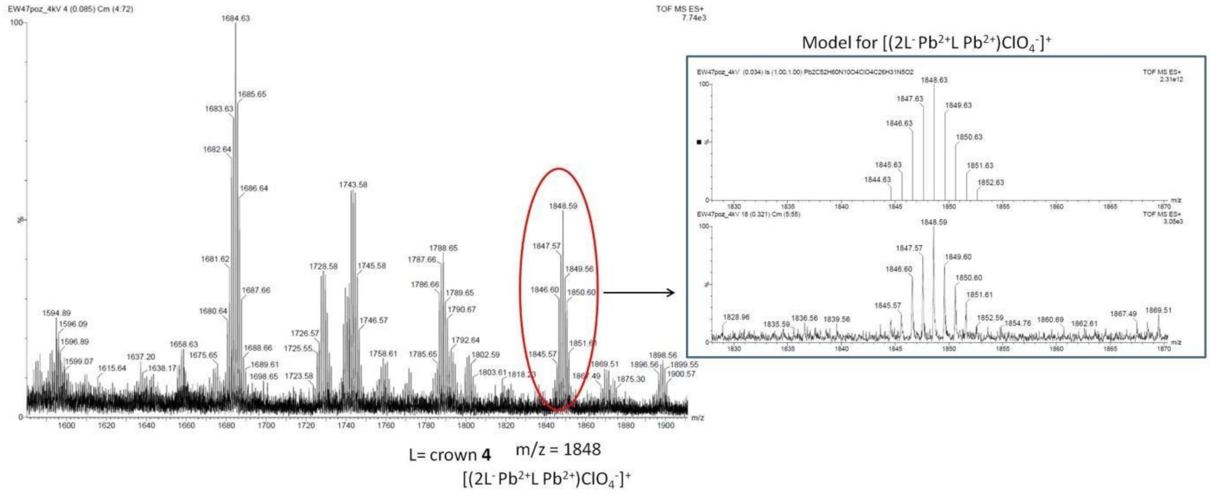

Figure S2e. Part of ESI-LR (positive ions mode) spectrum of lead(II)complex of **4** comparison of model of isotopic peaks for peak of m/z 1848.

2. Complexation Studies - Spectroscopic Methods

Table S1. Stability constant (logK) values of diazocrowns lead(II) complexes **1-4** in acetonitrile.

	**1**[1]	**2**	**3**	**4**
$logK_{L3Pb2}$	18.10 ± 0.01	21.10 ± 0.09	19.22 ± 0.05	18.37 ± 0.01

[1] E. Luboch, E. Wagner-Wysiecka, M. Fainerman-Melnikova, L.F. Lindoy, J.F. Biernat, Pyrrole Azocrown Ethers. Synthesis, Complexation, Selective Lead Transport and Ion-Selective Membrane Electrode Studies, *Supramol. Chem.*, 2006, **18**, 593-601.

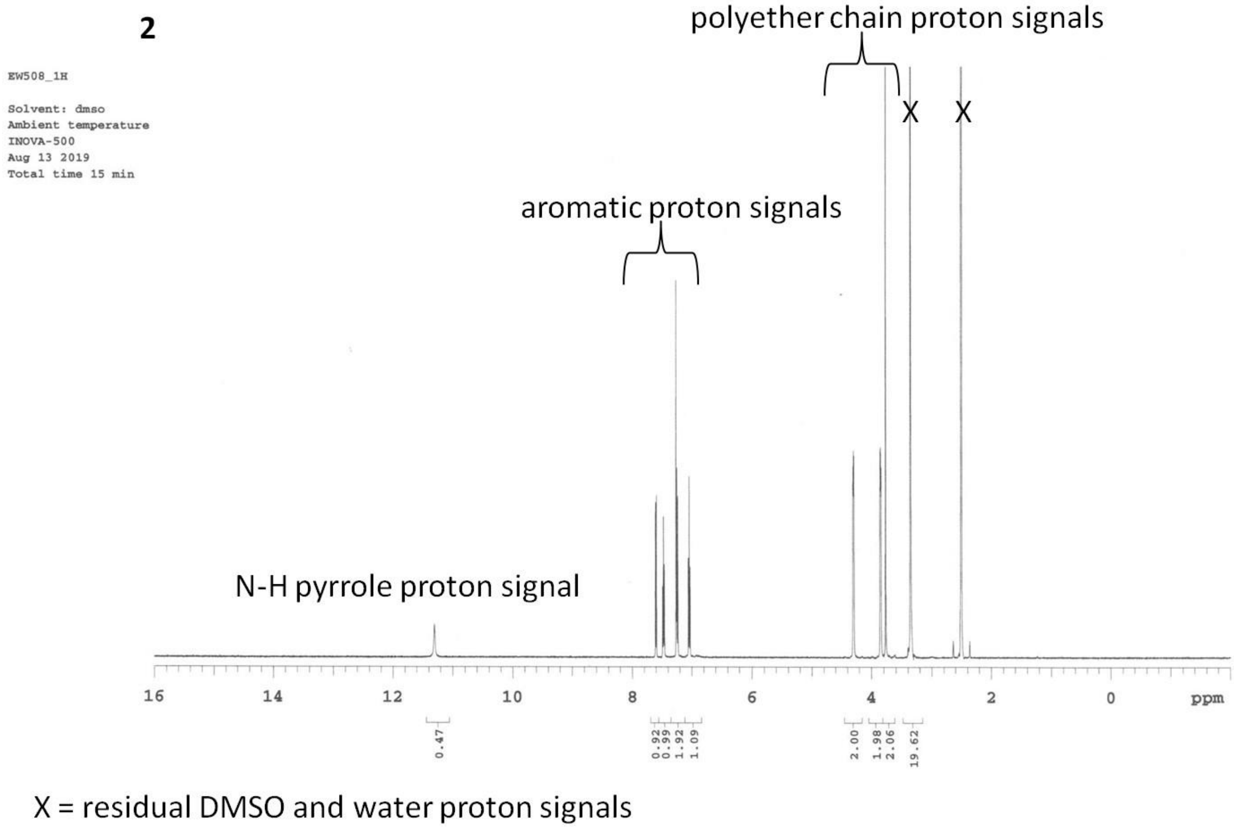

Figure S3a. ^1H NMR spectrum of **2** (1.6×10^{-2}M) (DMSO-d_6).

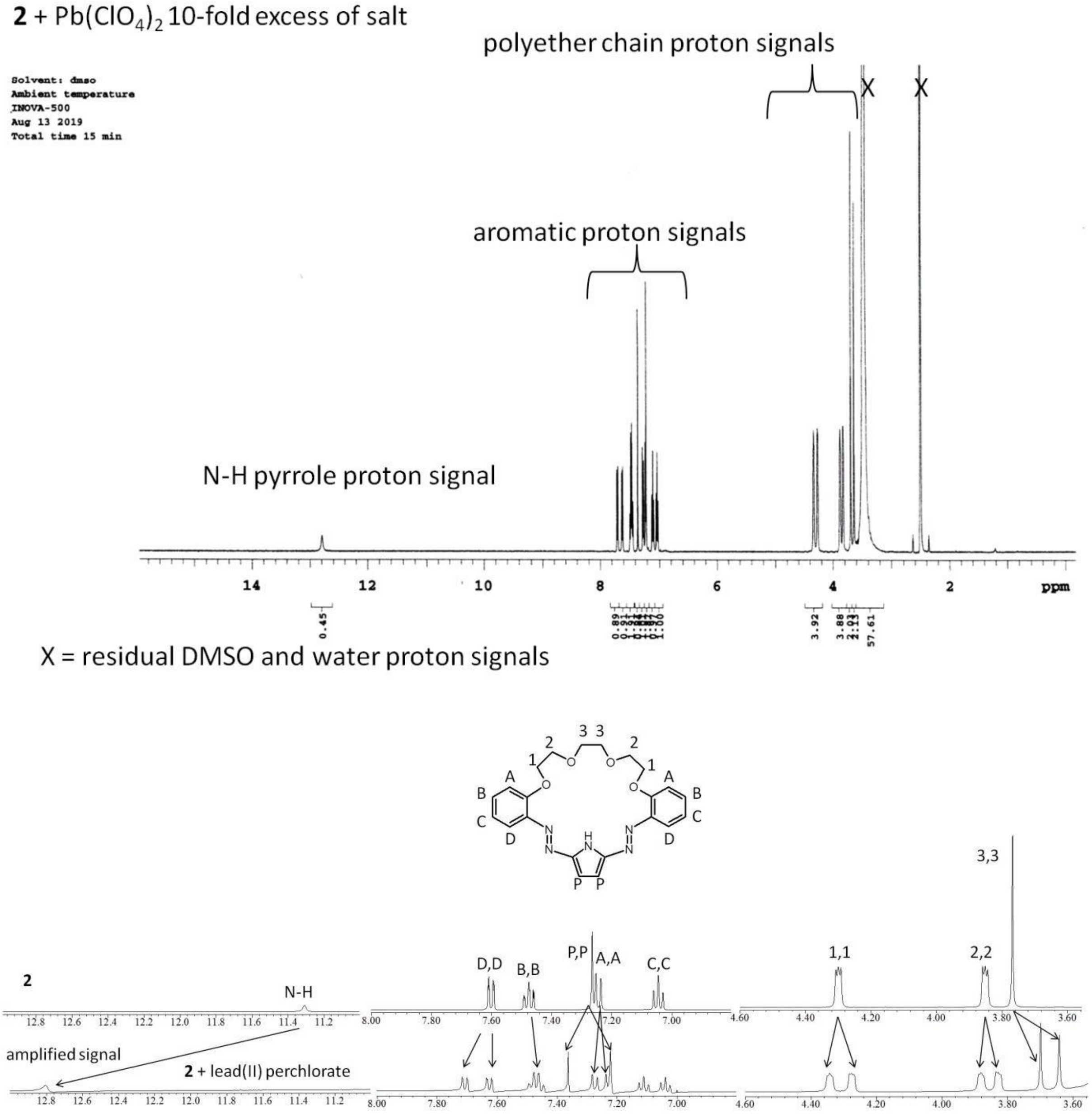

Figure S3b. Top: ^{1}H NMR spectrum of **2** (1.6×10^{-2} M) in the presence of 10-fold excess of lead(II) perchlorate; bottom: comparison of the spectral pattern of proton signals in free crown **2** and its spectrum in the presence of 10-fold excess lead(II) perchlorate (DMSO-d_6).

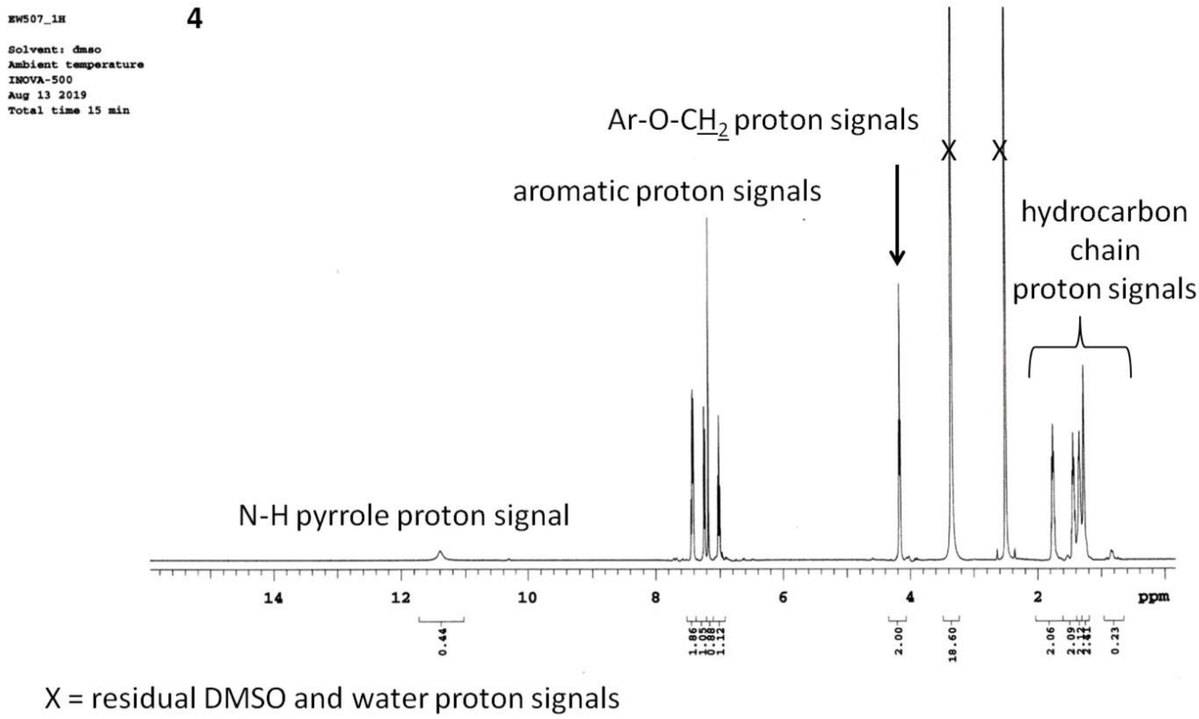

Figure S4a. ^{1}H NMR spectrum of **4** (1.6×10^{-2}M) (DMSO-d_6).

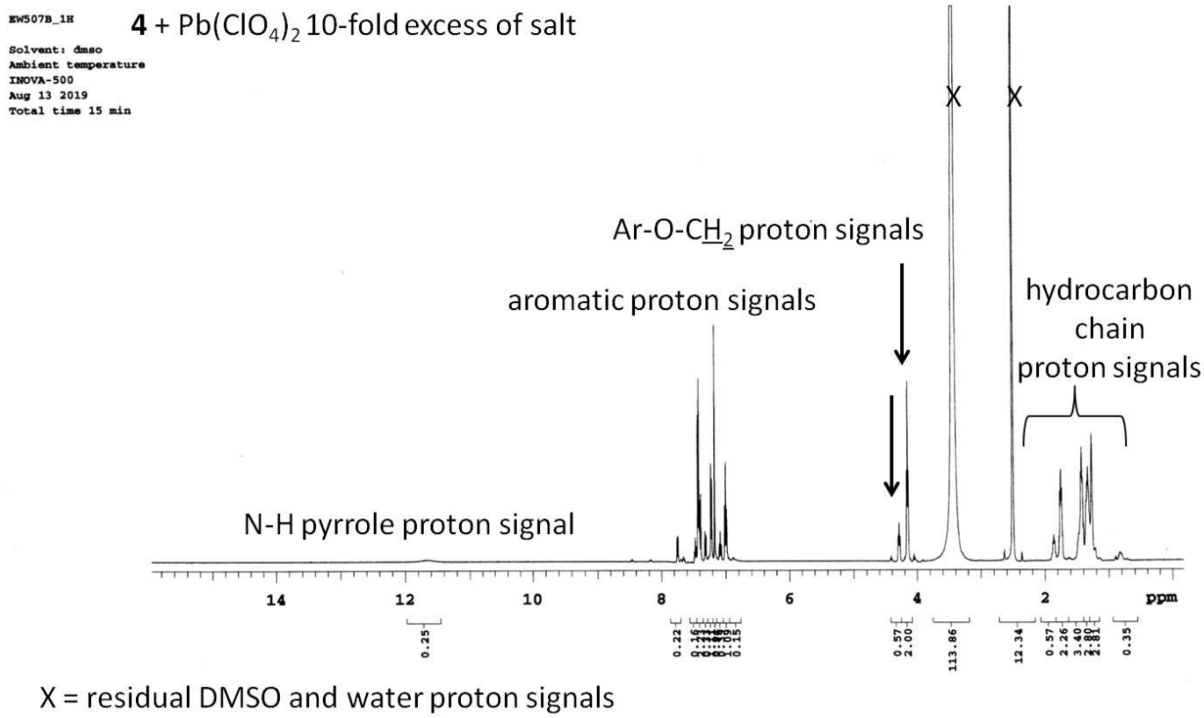

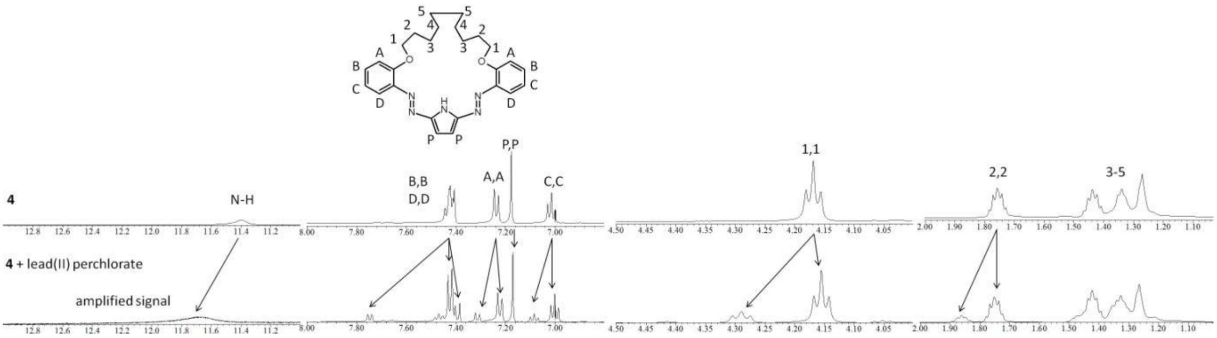

Figure S4b. Top: ^{1}H NMR spectrum of **4** (1.6×10^{-2} M) in the presence of 10-fold excess of lead(II) perchlorate; bottom comparison of the spectral pattern of proton signals in free crown **4** and its spectrum in the presence of 10-fold excess lead(II) perchlorate (DMSO-d_6).

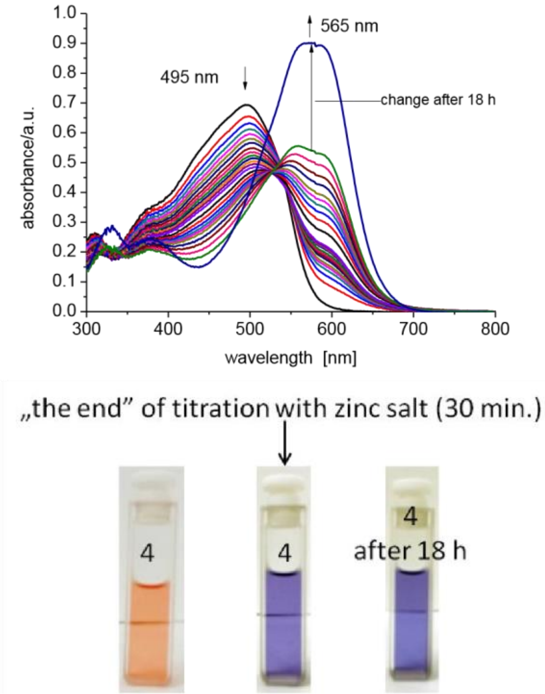

Figure S5. UV-Vis titration trace and color change for **4** ($c_4 = 3.14 \times 10^{-5}$ M) and zinc(II) perchlorate ($c_{Zn} = 01.76 \times 10^{-3}$ M) in acetonitrile. Bottom: color changes of the solutions.

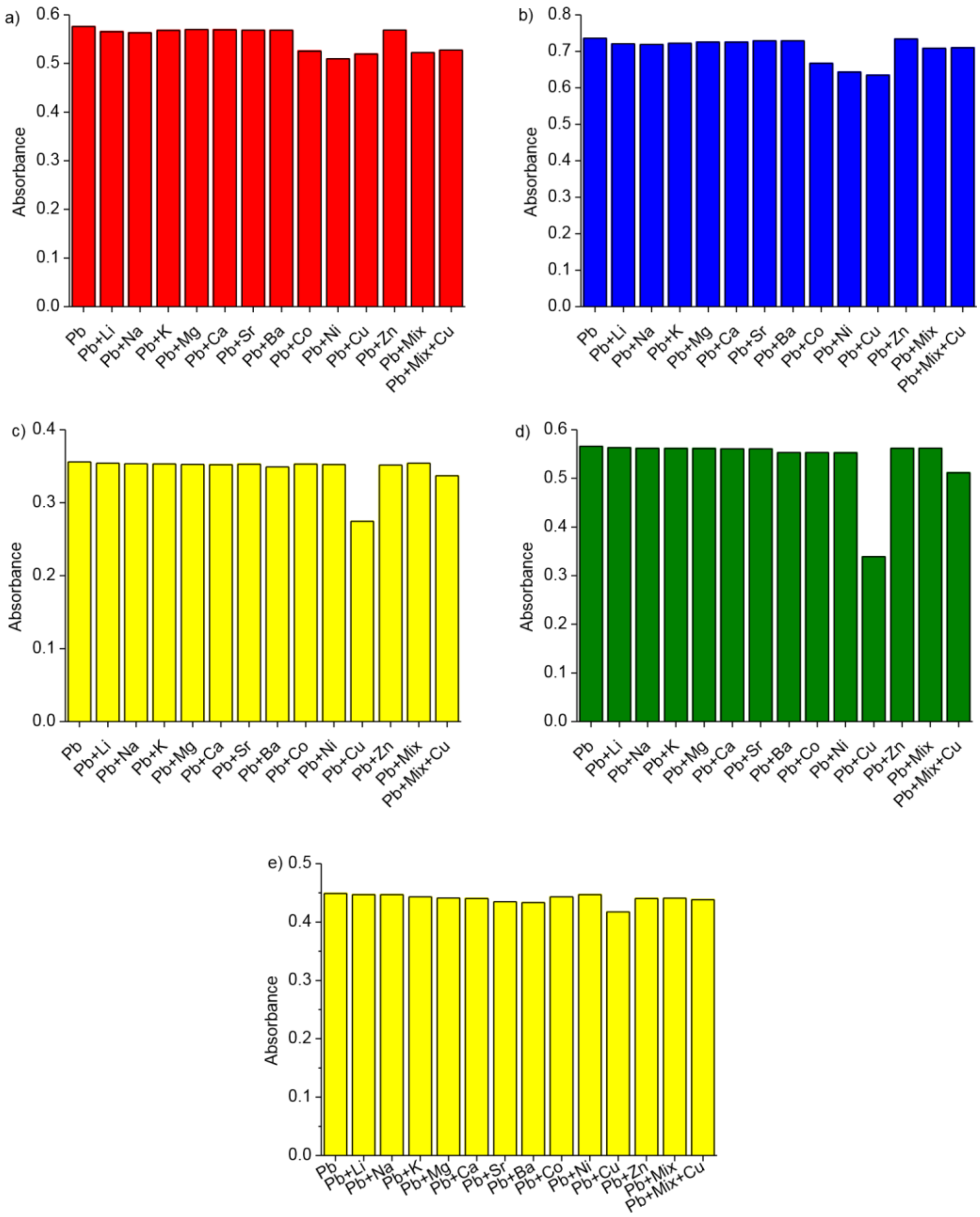

Figure S6. Interferences from several metal perchlorates, used in 10-fold excess, on spectrophotometric response towards systems containing equimolar amount of lead(II) perchlorate for (**a**) **1** (2.54 × 10^{-5} M) at 605 nm, (**b**) **2** (2.73 × 10^{-5} M) at 610 nm, (**c**) **3** (2.08 × 10^{-5} M) at 610 nm and (**d**) **4** (2.63 × 10^{-5} M) at 605 nm in acetonitrile (Mix—mixture of metal perchlorates without copper). (**e**) interferences from several metal perchlorates, used in 10-fold excess, on spectrophotometric response towards systems containing equimolar amount of lead(II) perchlorate for **3** (2.13 × 10^{-5} M) at 608 nm at equimolar (to crown) amount of lead(II) perchlorate in acetonitrile:water (9:1, v/v) solution at pH 5.

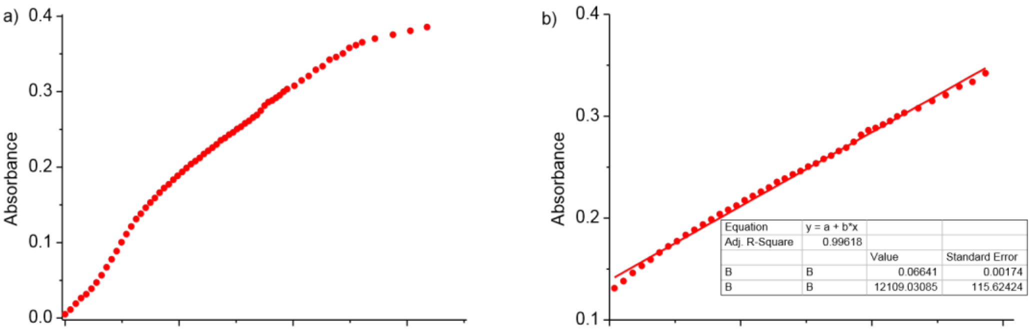

0.01.0 × 10^{-5} 2.0 × 10^{-5} 3.0 × 10^{-5} 6.0 × 10^{-6} 1.2 × 10^{-5} 1.8 × 10^{-5} 2.4 × 10^{-5}

cPb2+ cPb2+

Figure S7. (**a**) The relationship A = f([Pb(II)]) at 610 nm and (**b**) linear range of response towards lead(II) perchlorate for **3** in acetonitrile.

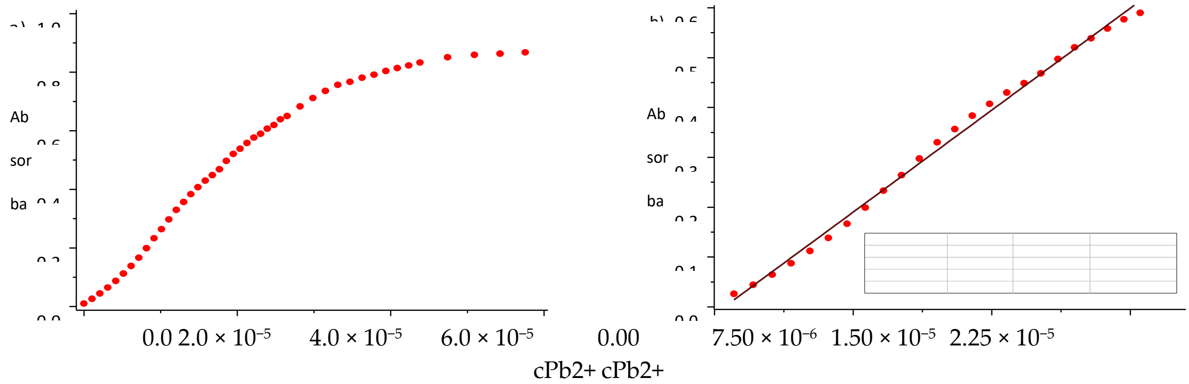

Figure S8. (**a**) The relationship A = f([Pb(II)]) at 605 nm and (**b**) linear range of response towards lead(II) perchlorate for **4** in acetonitrile.

(**a**) (**b**)

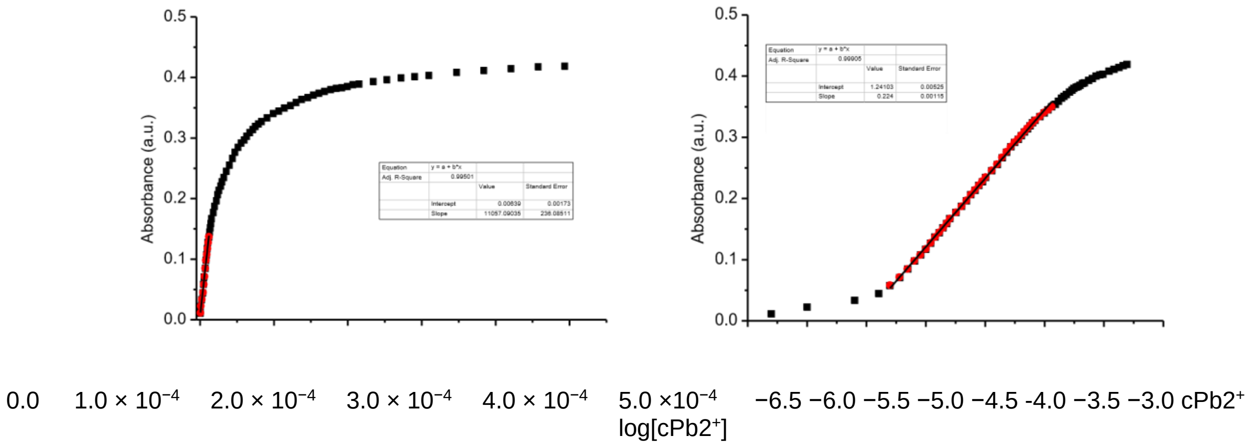

Figure S9. The relationship (**a**) A = f ([Pb(II)]) with linear range of response $5.00\times10^{-7} – 1.20\times10^{-5}$ M (**b**) A = f (log([Pb(II)]) with linear range of response $5.00 \times 10^{-6}–1.15 \times 10^{-4}$ M for **3** ($c_3 = 2.13 \times 10^{-5}$ M) in acetonitrile:water (9:1, v/v) solution at pH 5 at 608 nm.

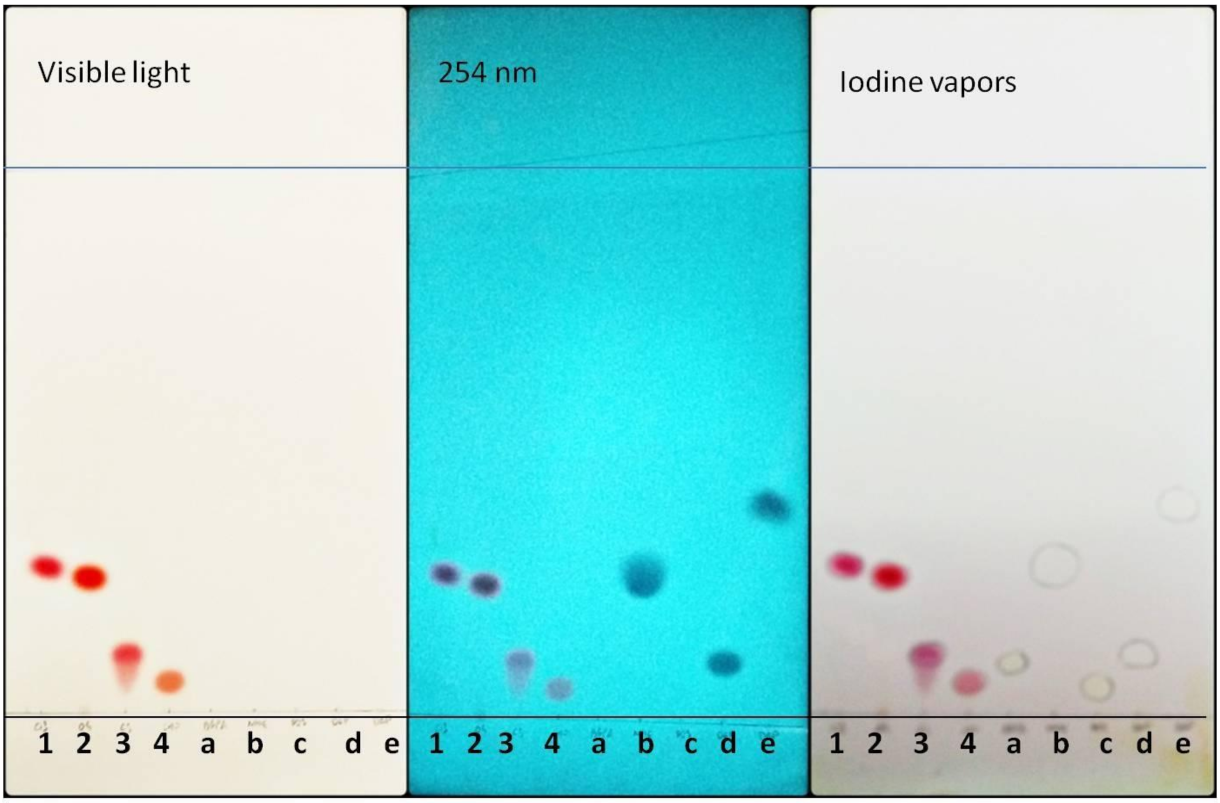

Figure S10. RP18-TLC chromatograms of crowns **1**–**4** and standard substances a–e.

On the basis of comparison of R_f values for standards and crowns **1-4** $logP_{TLC}$ values for macrocyles **1–4** were determined as follows: **1**: 5.54 ± 0.05, **2**: 5.67 ± 0.01, **3**: 8.12 ± 0.05 and **4**: 9.42 ± 0.03.

1 *(a)* E. Luboch, M. Jeszke, M. Szarmach, N. Łukasik, New bis(azobenzocrown)s with dodecylmethylmalonyl linkers as ionophores for sodium selective potentiometric sensors, *J. Incl. Phenom. Macrocycl. Chem.*, 2016, **86**, 323-335; *(b)* O. Dinten, U. E. Spichiger, N. Chaniotakis, P. Gehrig, B. Rusterholz, W. E. Morf, W. Simon, Lifetime of neutral-carrier-based liquid membranes in aqueous samples and blood and the lipophilicity of membrane components, *Anal. Chem.*, 1991, **63**, 596-603.

3. Ionic species detected in ESI mass spectra of lead(II) complex of crown 1

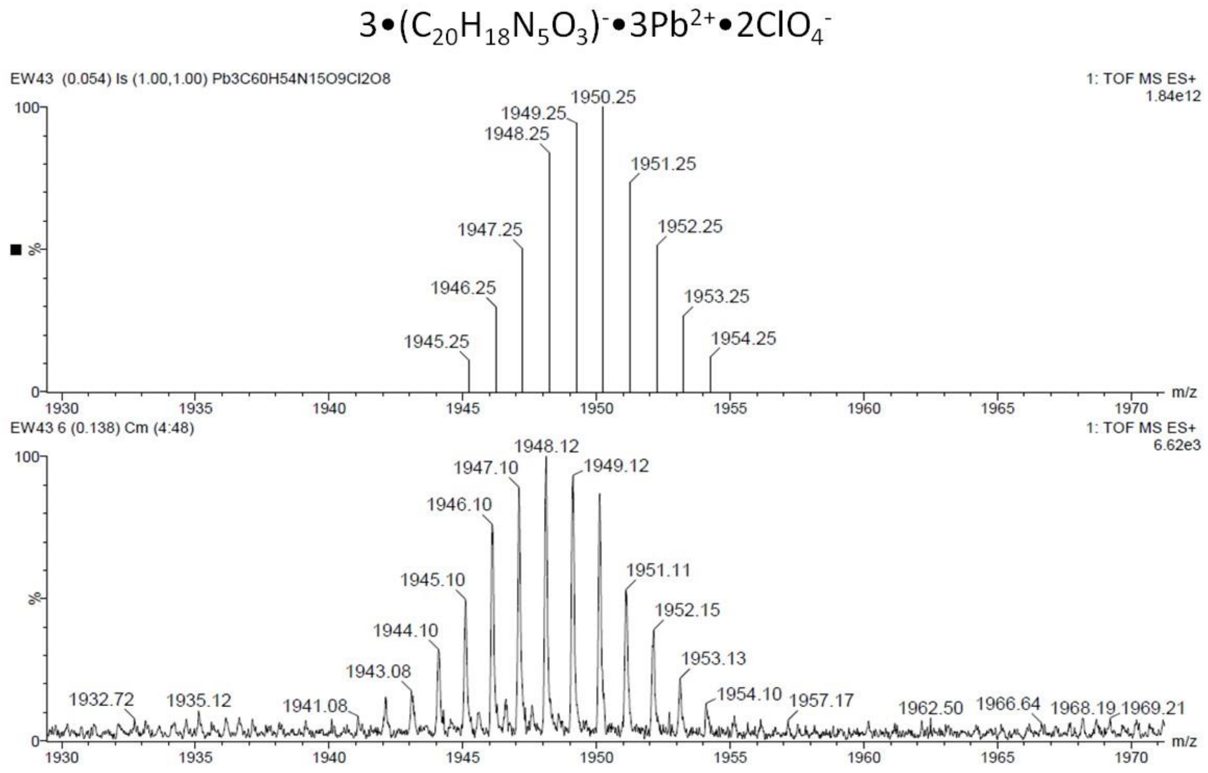

Figure S11a. Theoretically calculated and experimental isotope pattern of peak m/z 1950 in lead(II) complex of **1.**

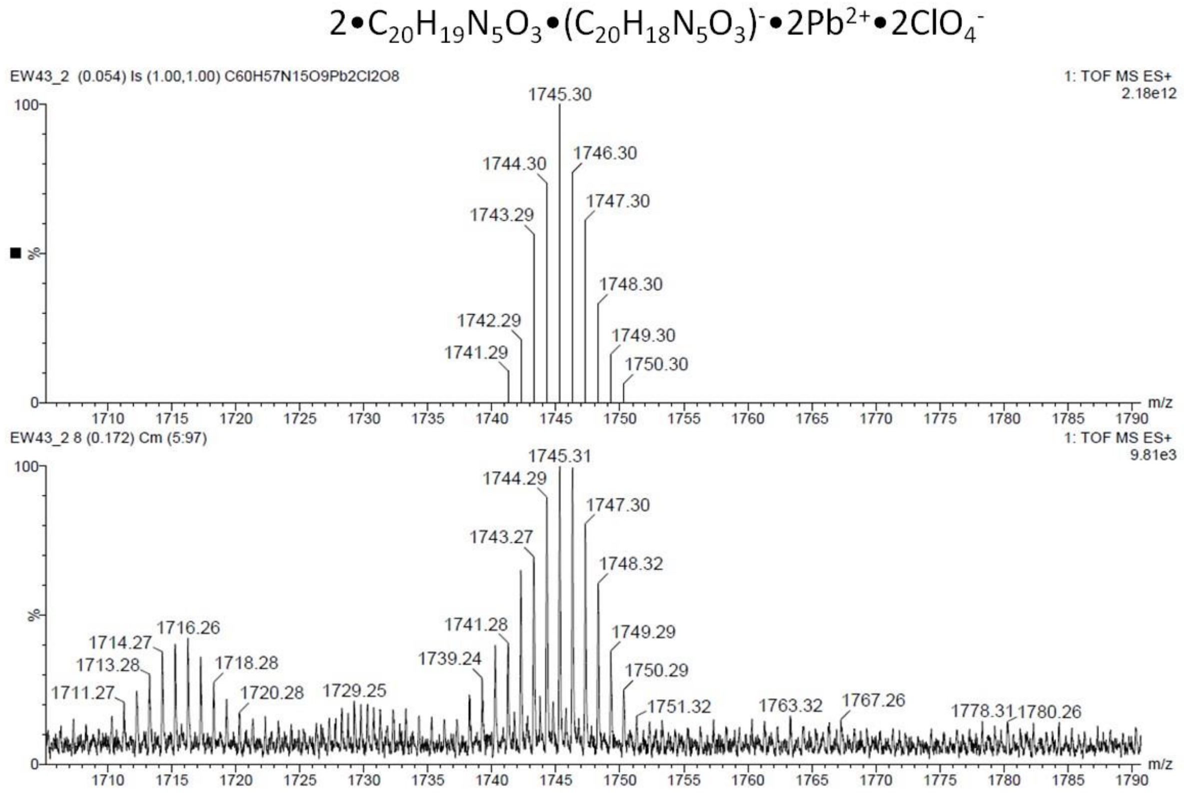

Figure S11b. Theoretically calculated and experimental isotope pattern of peak m/z 1745 in lead(II) complex of **1.**

$2\bullet(C_{20}H_{18}N_5O_3)^{-}\bullet C_{20}H_{19}N_5O_3\bullet 2Pb^{2+}\bullet ClO_4^{-}$

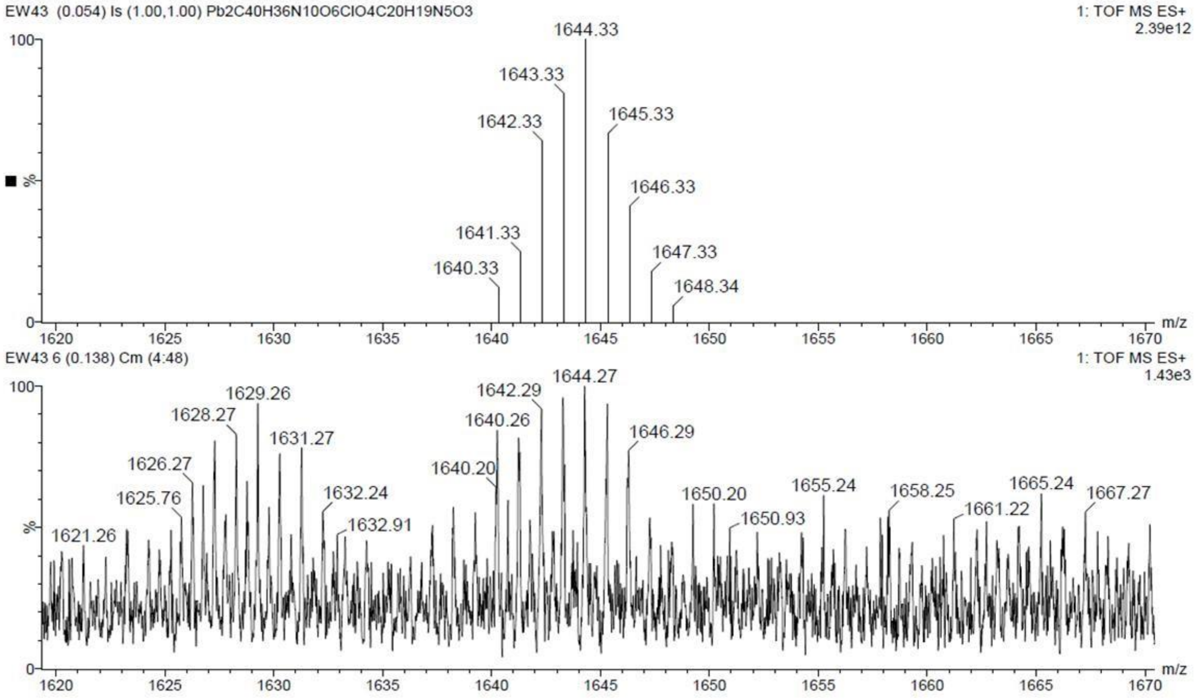

Figure S11c. Theoretically calculated and experimental isotope pattern of peak m/z 1644 in lead(II) complex of **1.**

$2\bullet(C_{20}H_{18}N_5O_3)^{-}\bullet 2Pb^{2+}\bullet ClO_4^{-}$

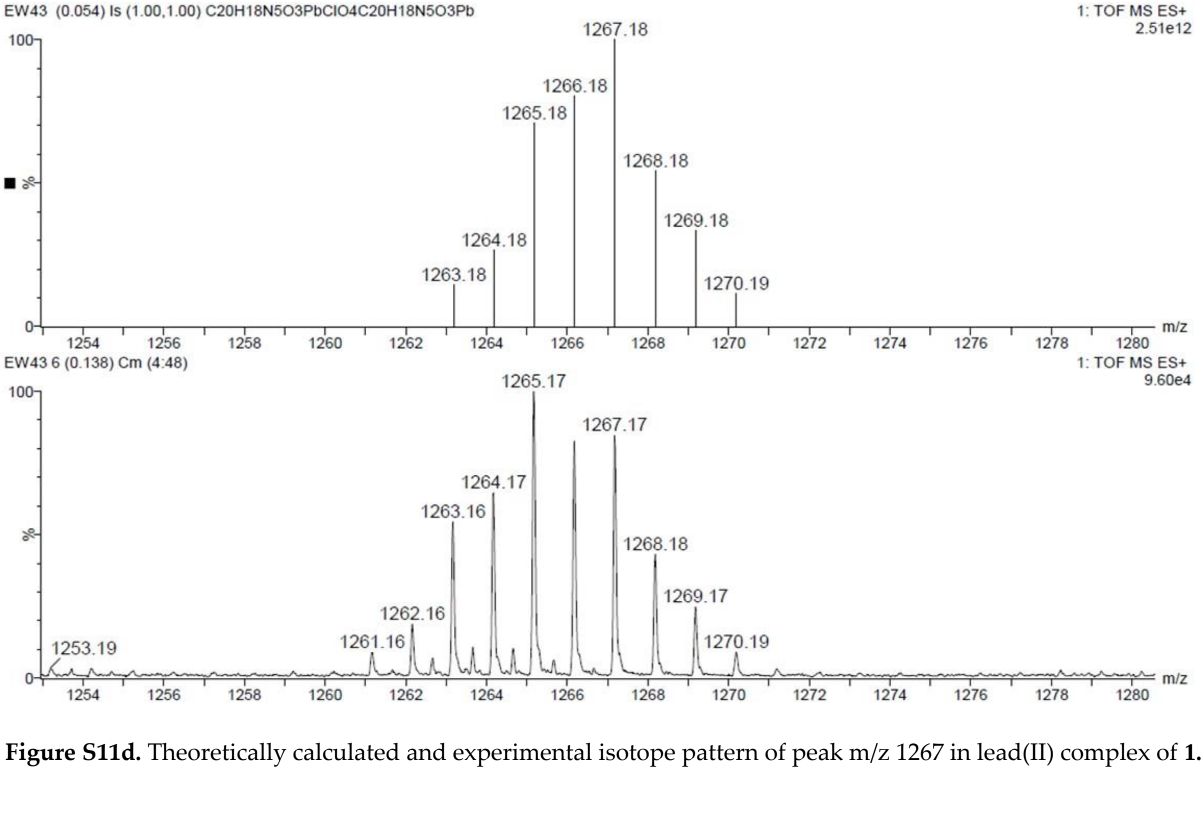

Figure S11d. Theoretically calculated and experimental isotope pattern of peak m/z 1267 in lead(II) complex of **1.**

$(C_{20}H_{18}N_5O_3)^{-}\bullet Pb^{2+}$

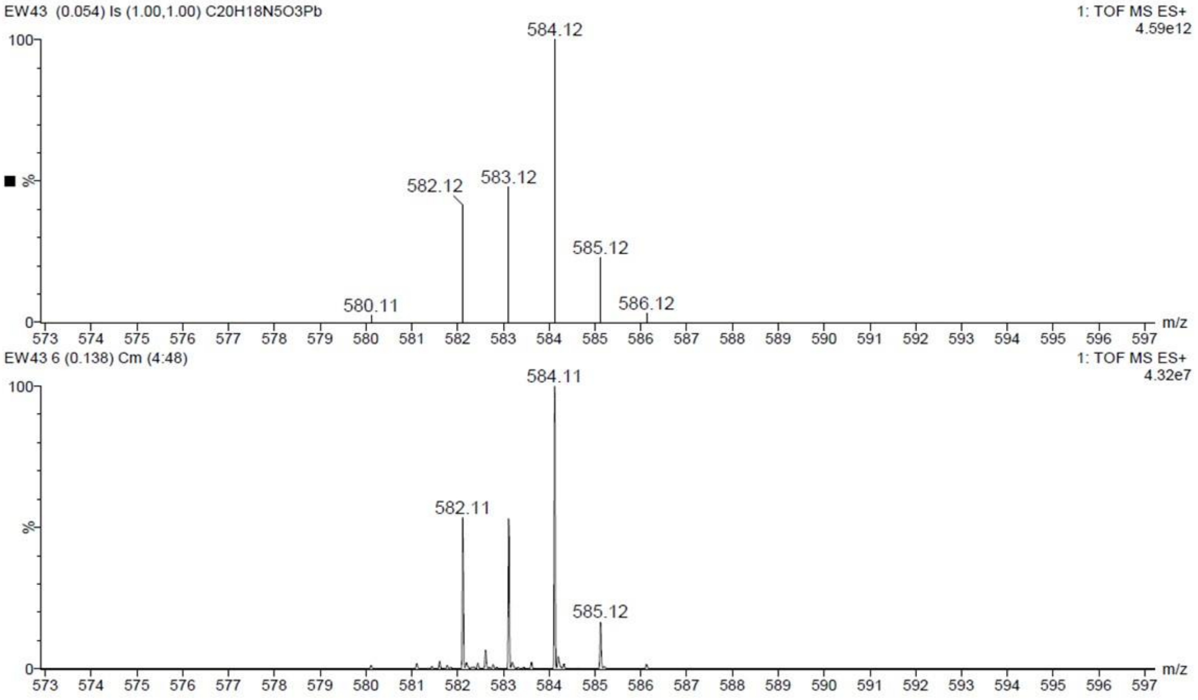

Figure S11e. Theoretically calculated and experimental isotope pattern of peak m/z 584 in lead(II) complex of **1.**

4. Ionic species detected in ESI mass spectra of lead(II) complex of crown 2

$3\bullet(C_{22}H_{22}N_5O_4)^{-}\bullet\ 3Pb^{2+}\bullet 2ClO_4^{-}$

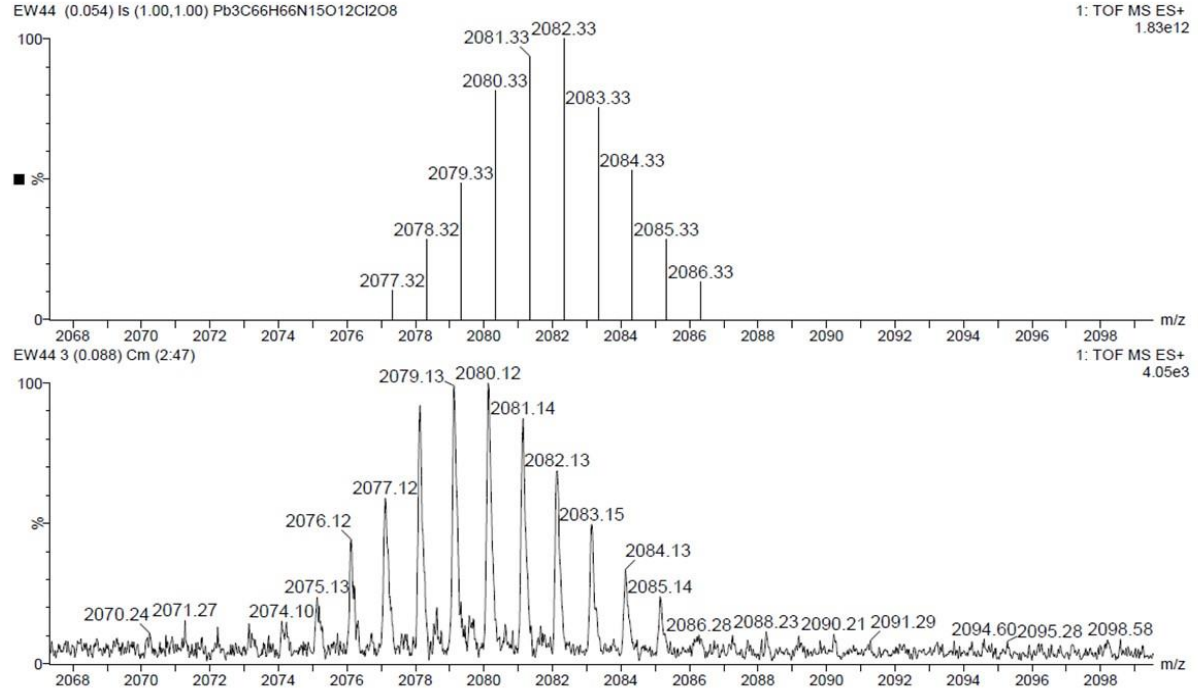

Figure S12a. Theoretically calculated and experimental isotope pattern of peak m/z 2082 in lead(II) complex of **2.**

$$2\bullet C_{22}H_{23}N_5O_4\bullet(C_{22}H_{22}N_5O_4)^{-}\bullet 2Pb^{2+}\bullet 2ClO_4^{-}$$

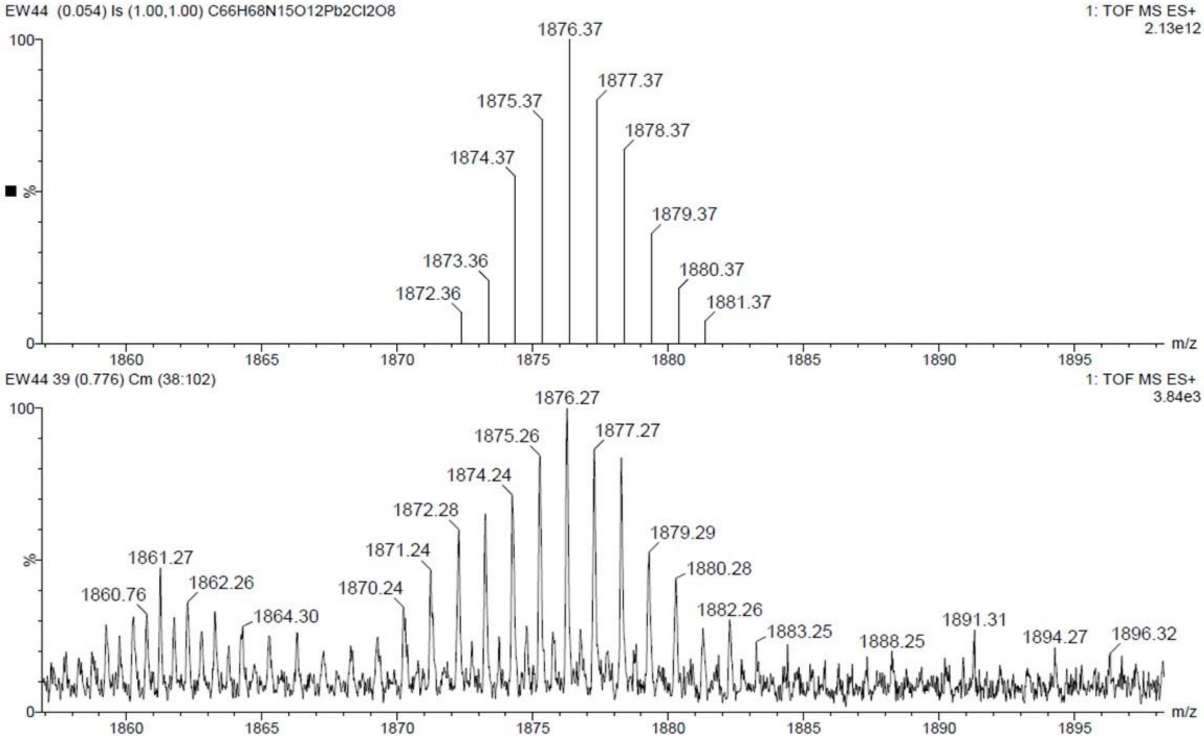

Figure S12b. Theoretically calculated and experimental isotope pattern of peak m/z 1876 in lead(II) complex of **2.**

$$2\bullet(C_{22}H_{22}N_5O_4)^{-}\bullet C_{22}H_{23}N_5O_4\bullet 2Pb^{2+}\bullet ClO_4^{-}$$

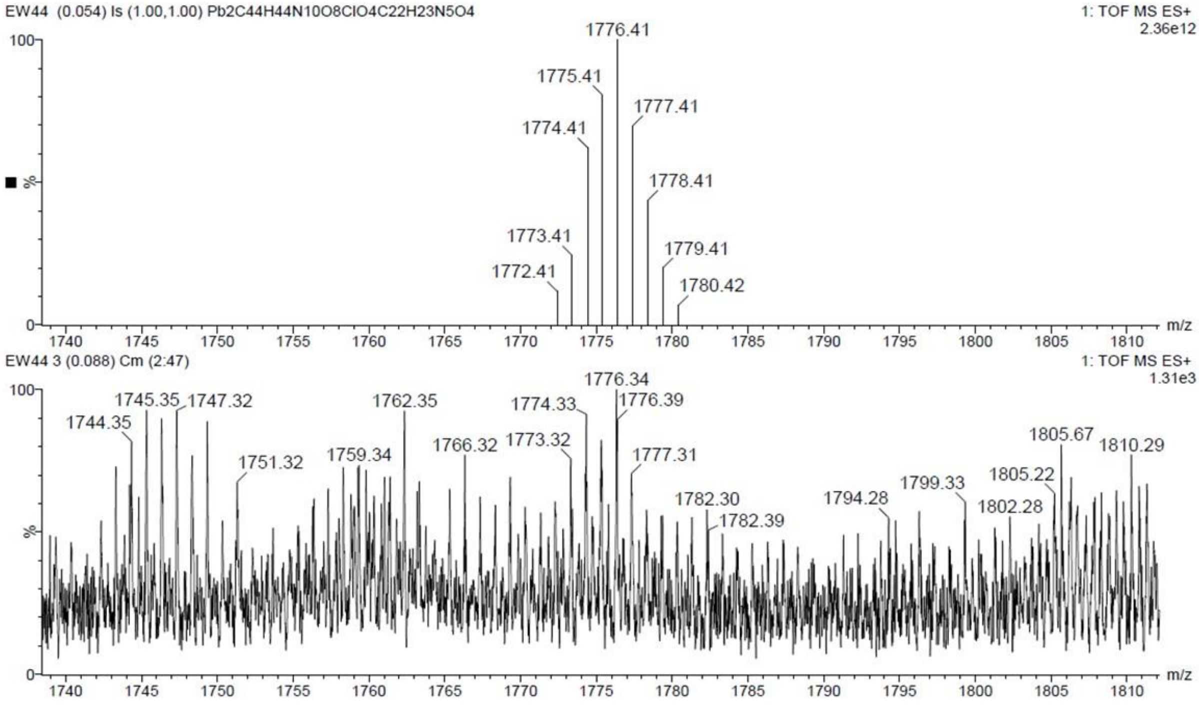

Figure S12c. Theoretically calculated and experimental isotope pattern of peak m/z 1776 in lead(II) complex of **2.**

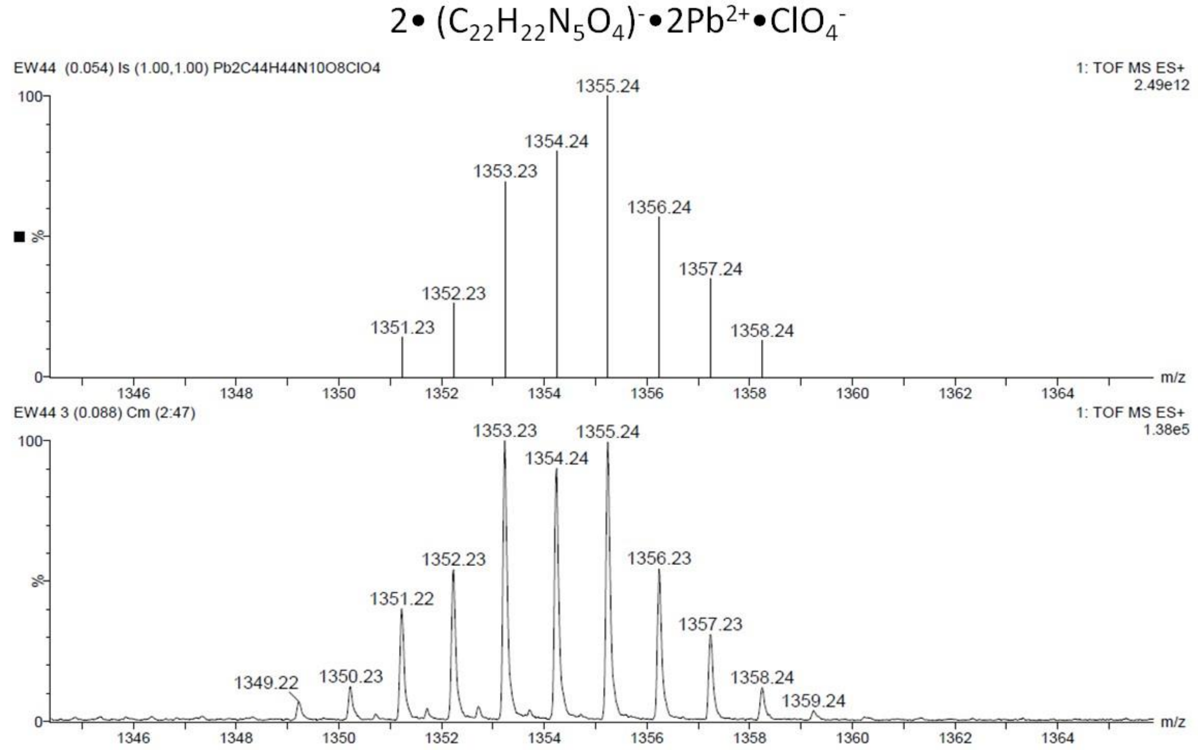

Figure S12d. Theoretically calculated and experimental isotope pattern of peak m/z 1335 in lead(II) complex of **2.**

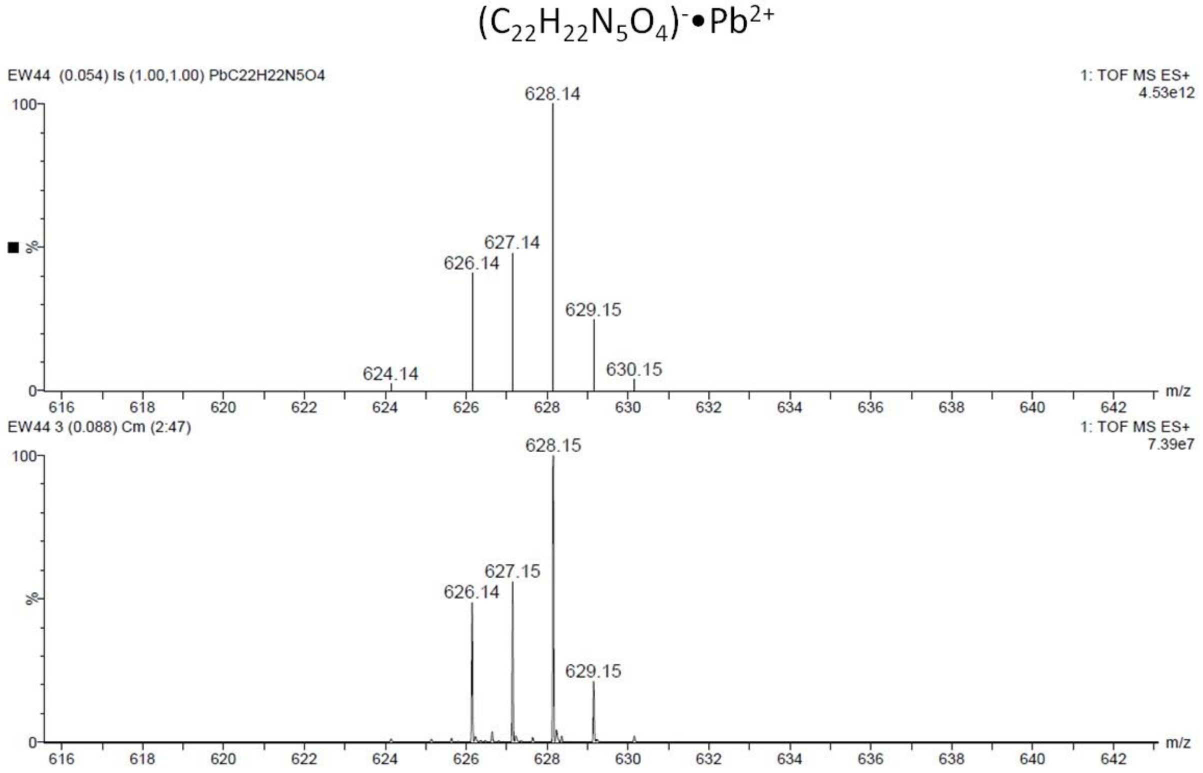

Figure S12e. Theoretically calculated and experimental isotope pattern of peak m/z 628 in lead(II) complex of **2.**

5. Ionic species detected in ESI mass spectra of lead (II) complex of crown 3

$3\bullet(C_{21}H_{20}N_5O_2)^{-}\bullet 3Pb^{2+}\bullet 2ClO_4^{-}$

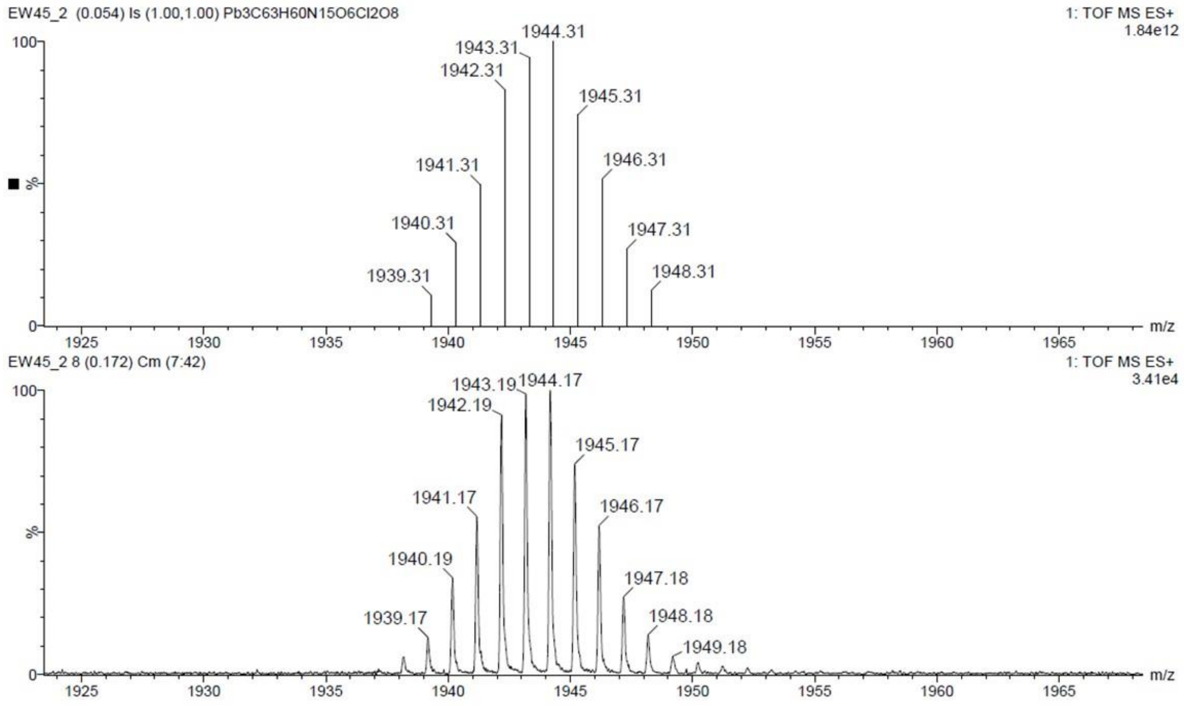

Figure S13a. Theoretically calculated and experimental isotope pattern of peak m/z 1944 in lead(II) complex of **3.**

$2\bullet(C_{21}H_{20}N_5O_2)^{-}\bullet C_{21}H_{21}N_5O_2\bullet 2Pb^{2+}\bullet ClO_4^{-}$

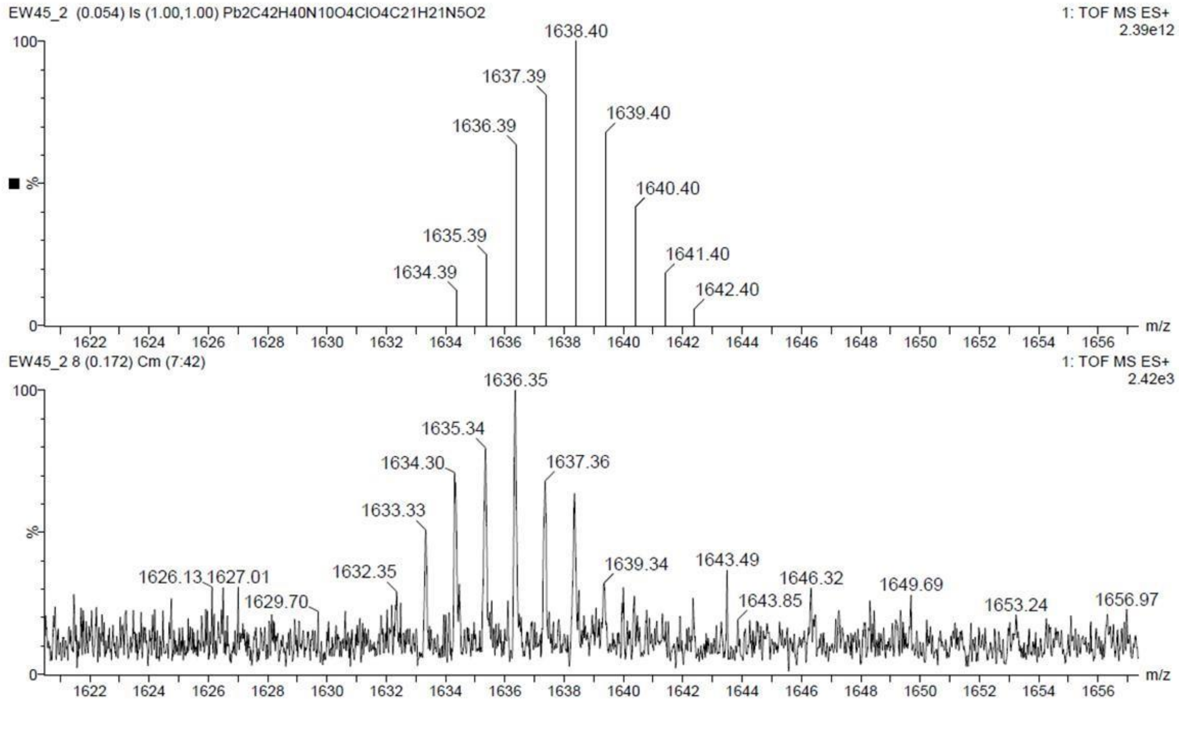

Figure S13b. Theoretically calculated and experimental isotope pattern of peak m/z 1636 in lead(II) complex of **3.**

$2\bullet(C_{21}H_{20}N_5O_2)^{-}\bullet 2Pb^{2+}\bullet ClO_4^{-}$

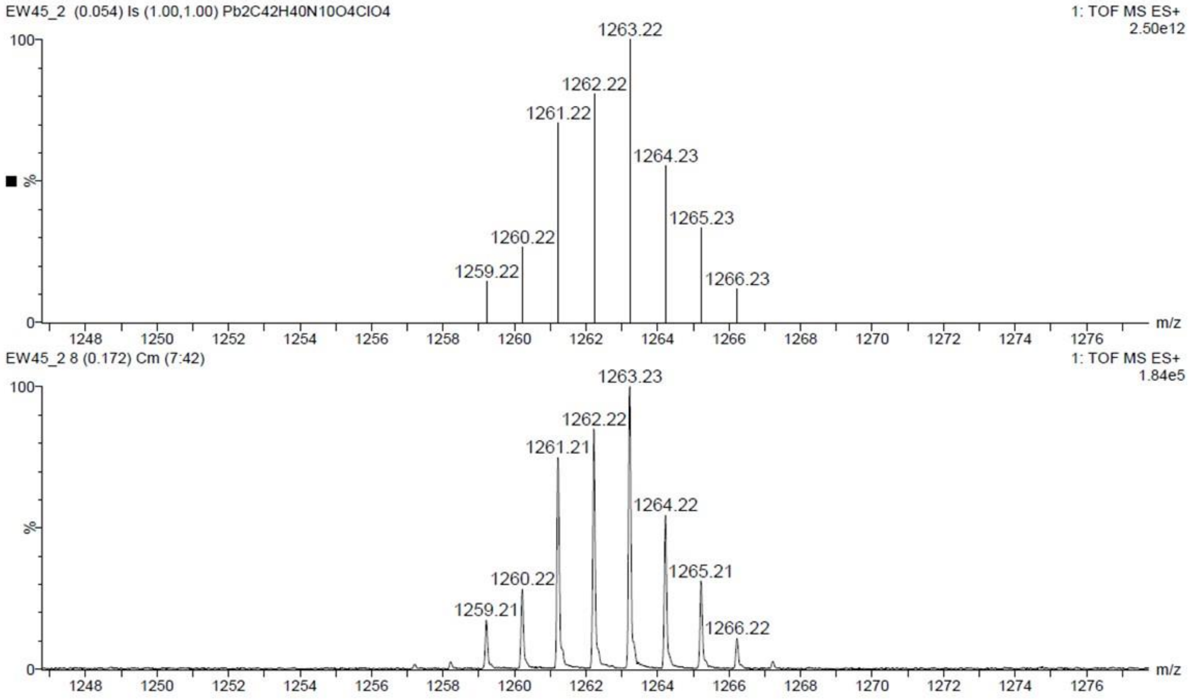

Figure S13c. Theoretically calculated and experimental isotope pattern of peak m/z 1263 in lead(II) complex of **3.**

$(C_{21}H_{20}N_5O_2)^{-}\bullet C_{21}H_{21}N_5O_2\bullet Pb^{2+}$

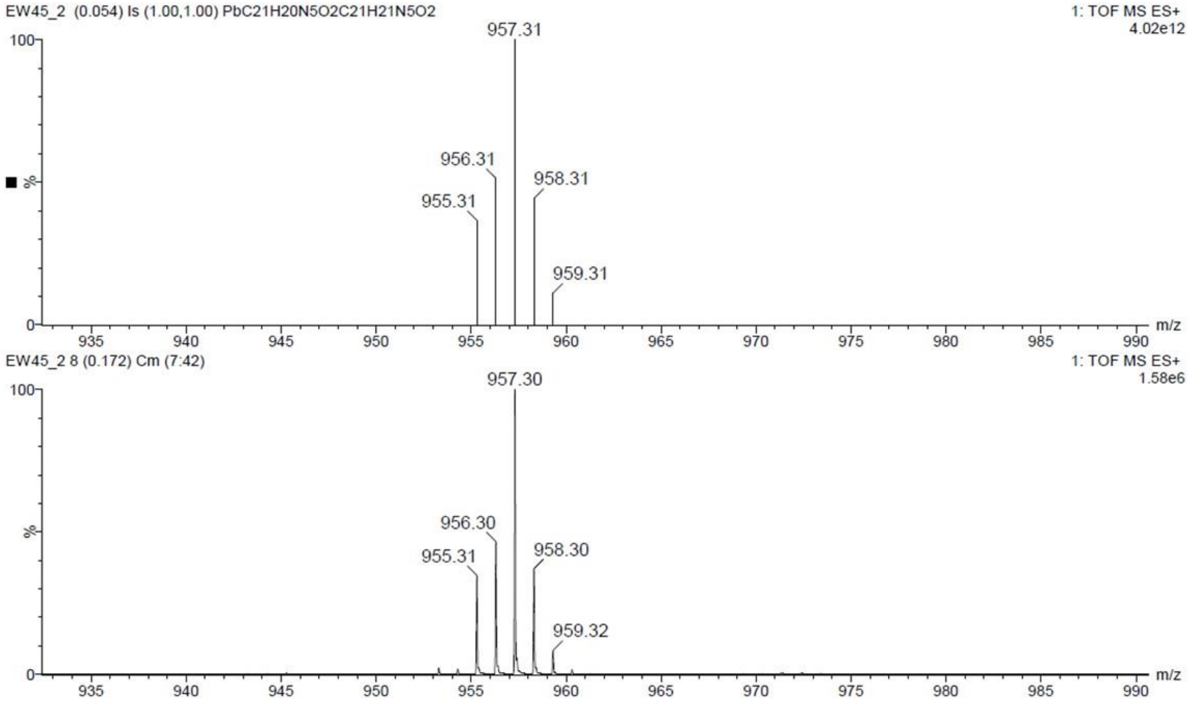

Figure S13d. Theoretically calculated and experimental isotope pattern of peak m/z 957 in lead(II) complex of **3.**

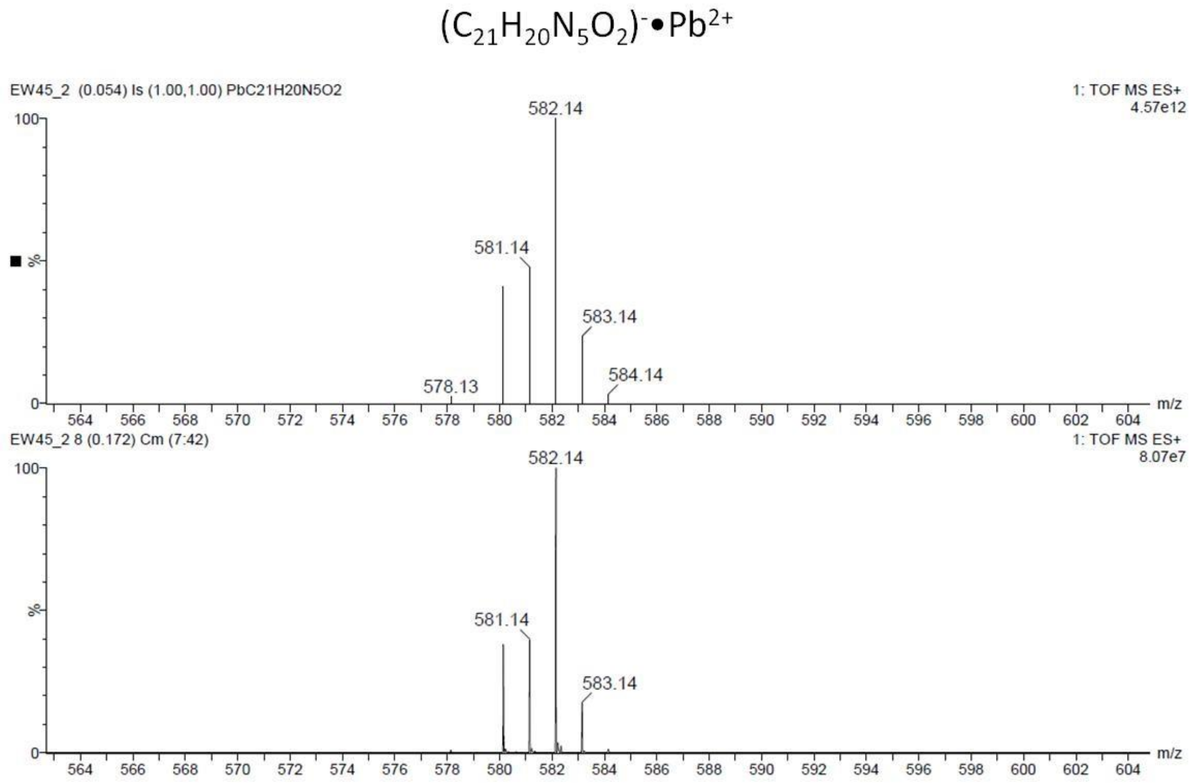

Figure S13e. Theoretically calculated and experimental isotope pattern of peak m/z 582 in lead(II) complex of **3.**

6. Ionic species detected in ESI mass spectra of lead (II) complex of crown 4

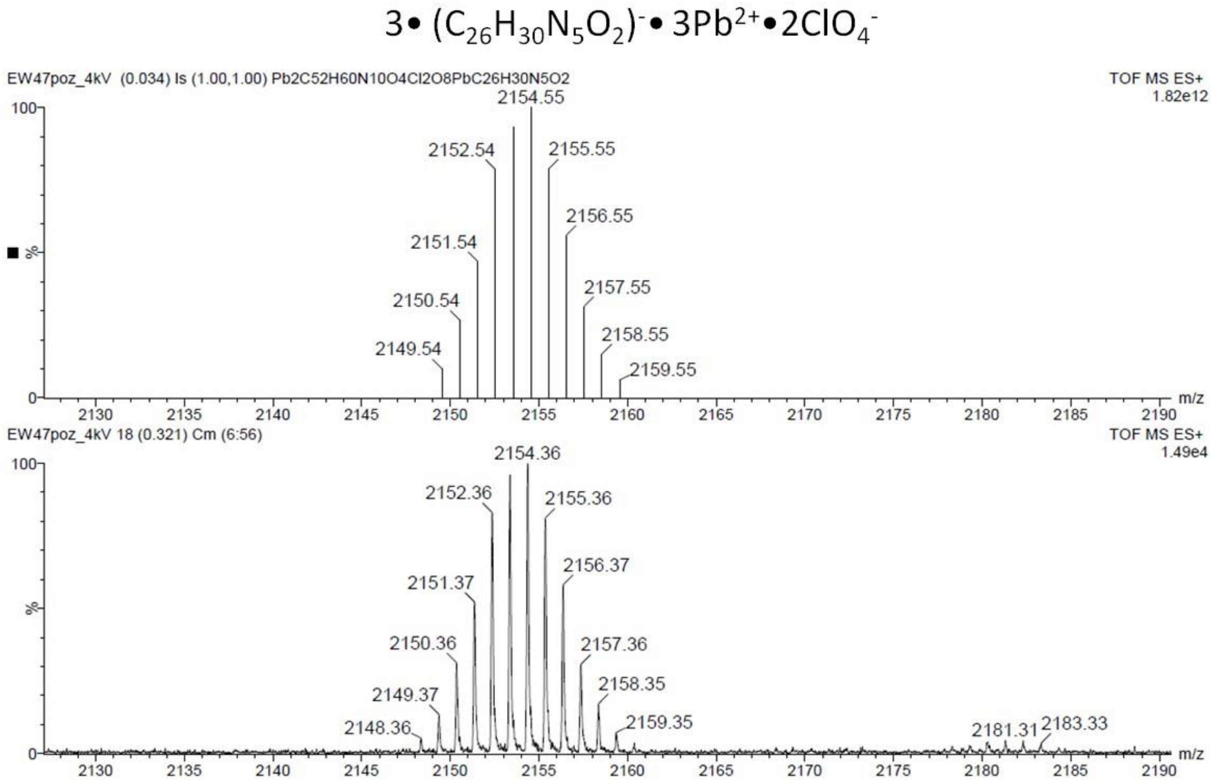

Figure S14a. Theoretically calculated and experimental isotope pattern of peak m/z 2154 in lead(II) complex of **4.**

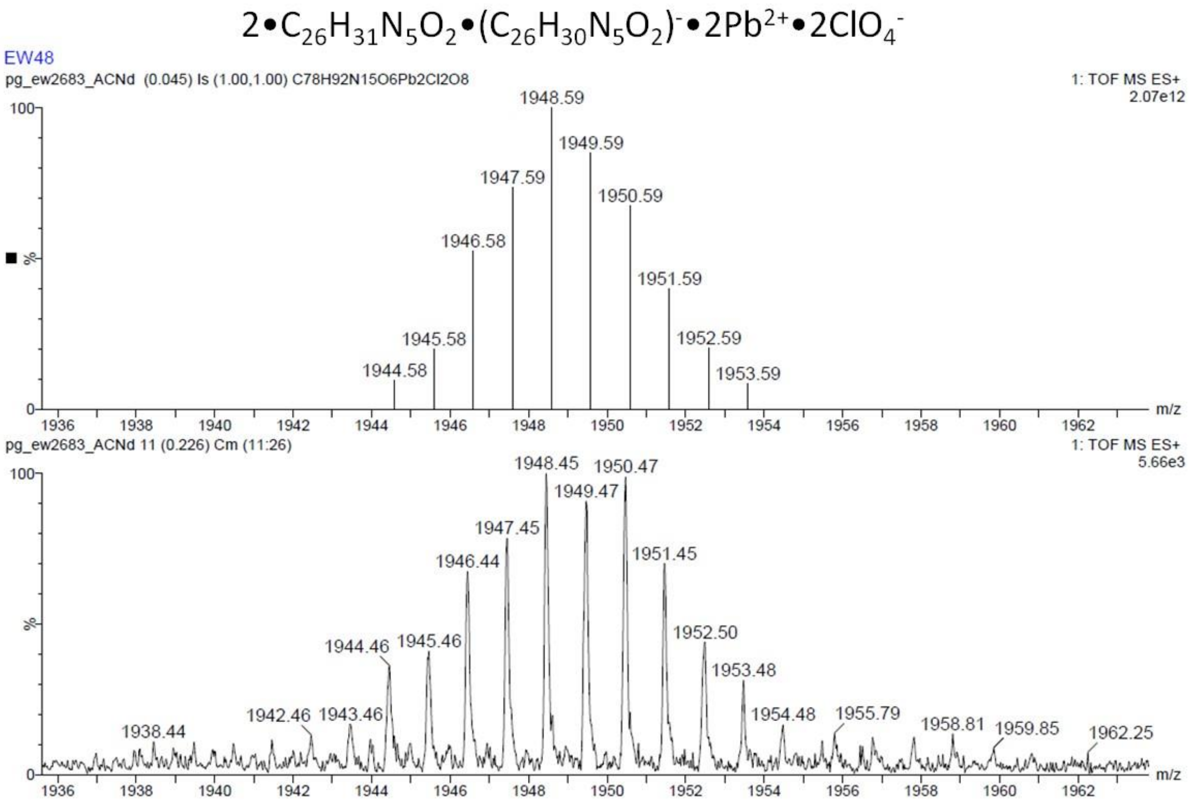

Figure S14b. Theoretically calculated and experimental isotope pattern of peak m/z 1948 in lead(II) complex of **4.**

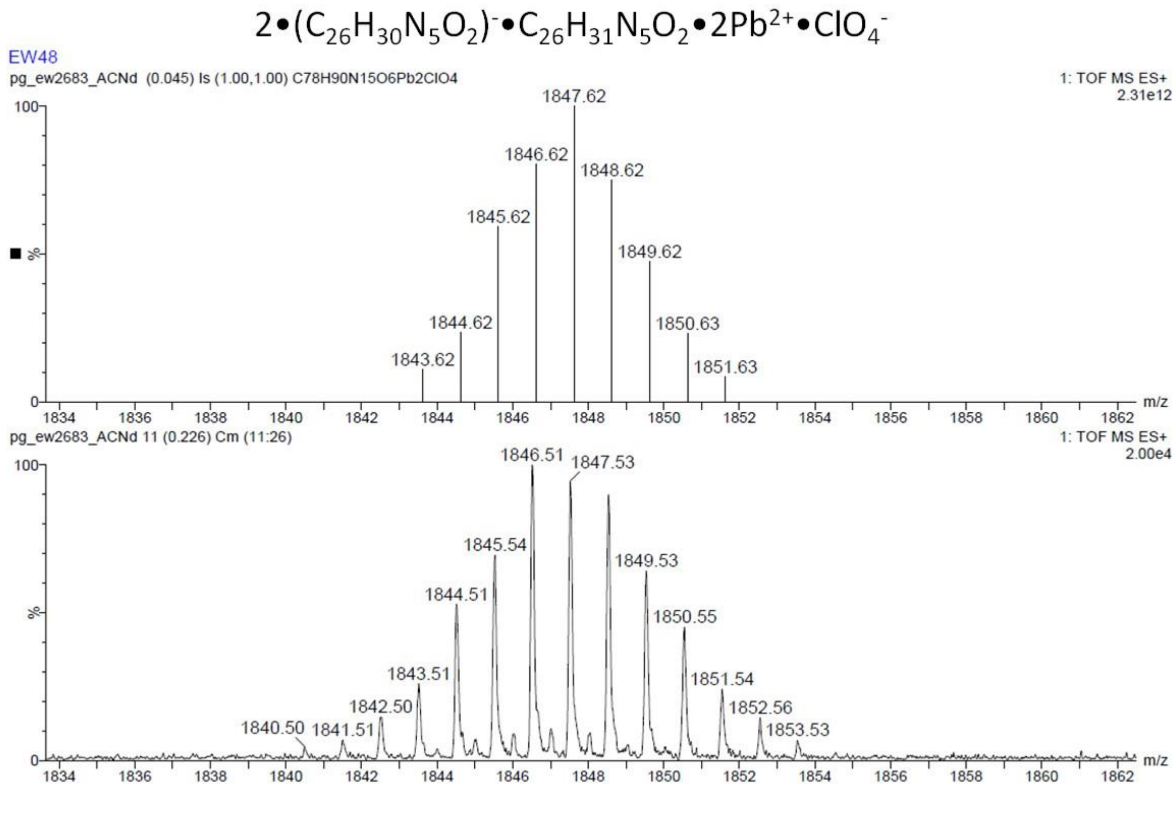

Figure S14c. Theoretically calculated and experimental isotope pattern of peak m/z 1848 in lead(II) complex of **4.**

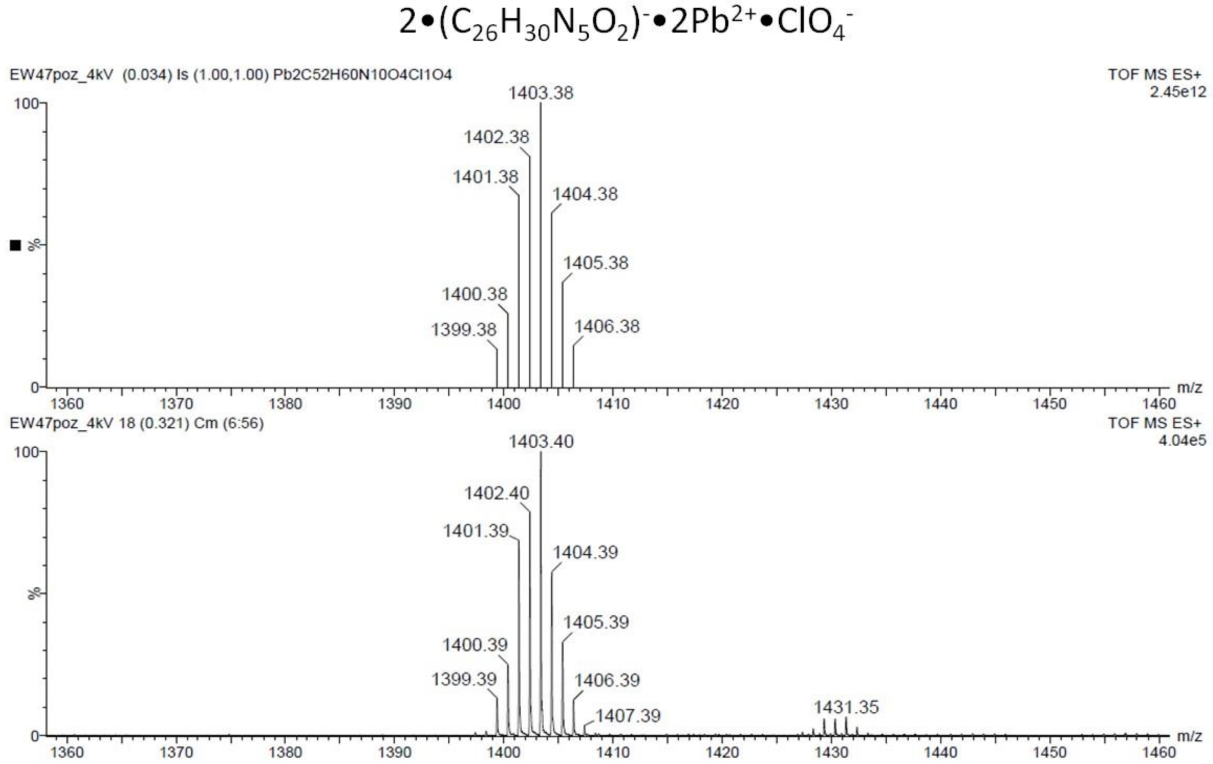

Figure S14d. Theoretically calculated and experimental isotope pattern of peak m/z 1403 in lead(II) complex of **4.**

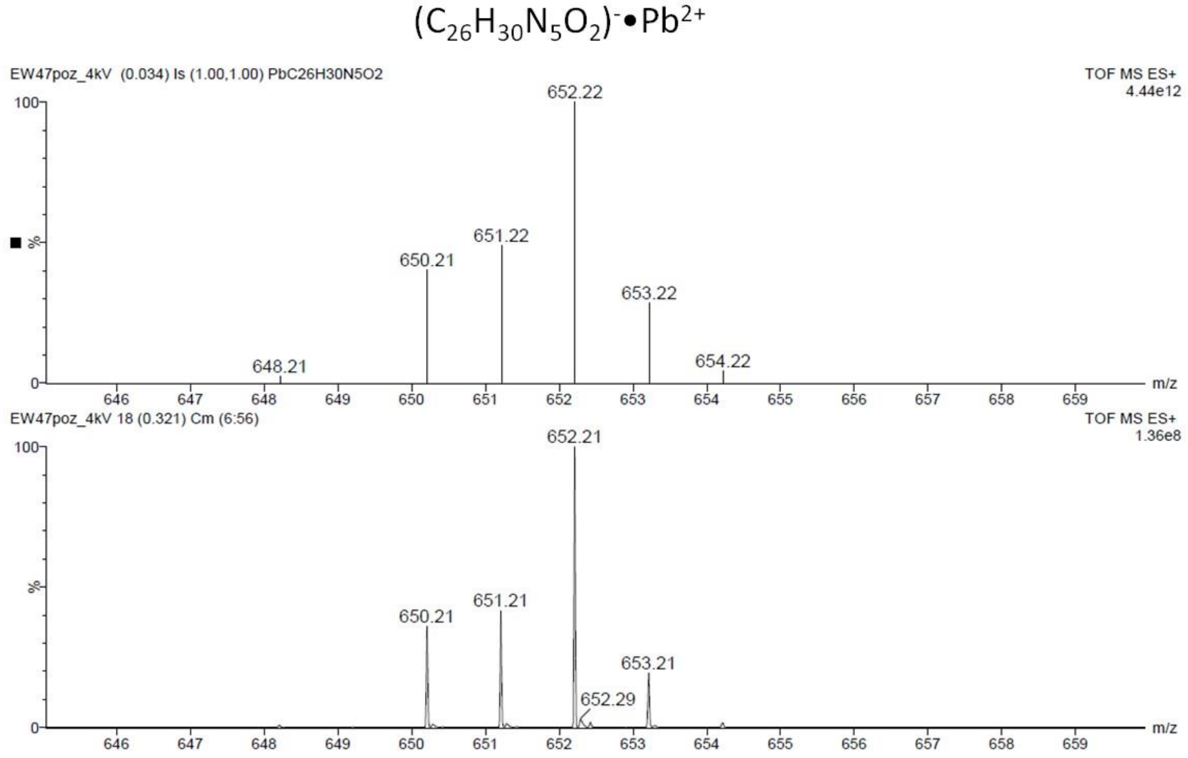

Figure S14e. Theoretically calculated and experimental isotope pattern of peak m/z 652 in lead(II) complex of **4.**

7. X-ray structure of 3

7.1. Crystallographic Details

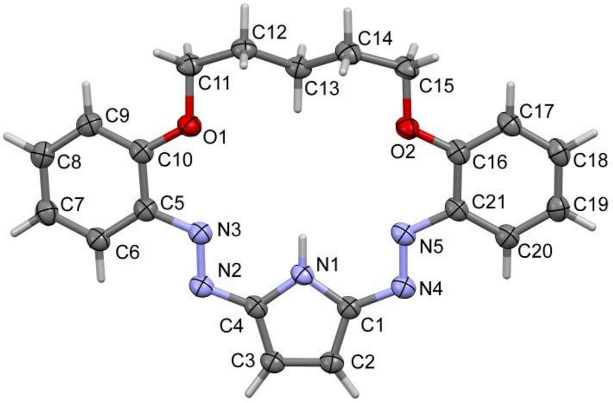

Figure S15. Molecular view of **3** showing atom labeling scheme. Displacement ellipsoids drawn at 50% probability level. Selected bond lengths (Å) and angles (°): N2-N3 1.278(2), N4-N5 1.281(2), C1-C2 1.391(2), C2-C3 1.398 (3), C3-C4 1.389 (3), O1-C11 1.436 (2), O2-C15 1.438 (2), C12-C13 1.532 (3), C13-C14 1.529 (3); valence angles: C1-N1-C4 108.74 (15), N1-C1-N4 123.38 (16), C1N4-N5 110.68 (15), N4-N5-C21 114.78 (15), C10-O1-C11 118.38 (14), C15-O2-C16 118.10 (15), C12-C13-C15 109.22 (15); torsions C1-N4N5-C21 -178.64 (14), C4-N2-N5-C5 175.42 (15).

Table S2. Crystal data and structure refinement details for **3**.

	**3**
CCDC no.	2081633
Empirical formula	C21H21N5O2
M_r/g mol^{-1}	375.43
Temperature/K	120 K
Crystal system	Orthorhombic
Space group (IT No.)	*Pbca* (61)
a/Å	9.4220 (4)
b/Å	16.9763 (6)
c/Å	22.8215 (7)
α/°	90
β/°	90
γ/°	90
Volume/Å^3	3650.3 (2)
Z	8
ϱcalc g/cm3	1.366
Crystal size/mm^3	0.42 × 0.11 × 0.02
Radiation	Mo Kα (λ = 0.71073)
2Θ range for data collection/°	4.8–58.6
Reflections collected/unique	19075/4927
Completeness to θ_{max} (%)	99.1
Data/restraints/parameters	3615/0/253
Goodness-of-fit on F^2	1.016
Final R indexes [I>=2σ (I)]	R_1 = 0.0595, wR_2 = 0.1451
Final R indexes [all data]	R_1 = 0.0846, wR_2 = 0.1654
Largest diff. peak/hole / e Å^{-3}	0.268/ -0.268

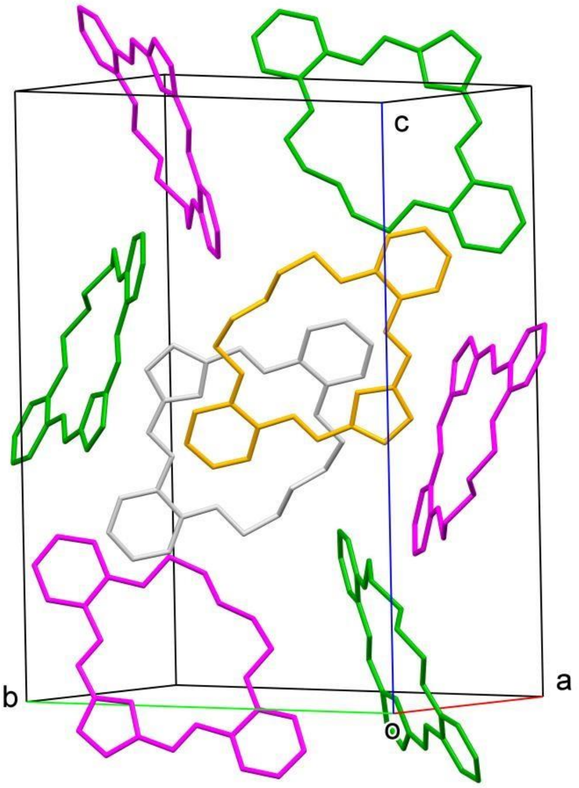

Figure S16. Crystal packing in **3**. Some molecules form stacking layers in parallel, but some pack almost perpendicular to them making complex space-filling pattern. Molecules are coloured by symmetry operation type, hydrogen atoms omitted.

Experimental details

**Crystal data**	
Chemical formula	C21H21N5O2
M_r	375.43
Crystal system, space group	Orthorhombic, *Pbca*
Temperature (K)	120
a, *b*, *c* (Å)	9.4220 (4), 16.9763 (6), 22.8215 (7)
V (Å^3)	3650.3 (2)
Z	8
Radiation type	Mo *K*
(mm^{-1})	0.09
Crystal size (mm)	0.42 × 0.11 × 0.02
Data collection	
Diffractometer	STOE *IPDS* 2T
Absorption correction	–
No. of measured, independent and observed [*I* > 2 (*I*)] reflections	19075, 4927, 3615
R_{int}	0.046
(sin /)$_{max}$ (Å^{-1})	0.688
Refinement	
$R[F^2 > 2 (F^2)]$, $wR(F^2)$, S	0.060, 0.164, 1.02
No. of reflections	4927
No. of parameters	253
H-atom treatment	H-atom parameters constrained
max, min (e Å^{-3})	0.27, -0.27

Computer programs: *X-AREA* WinXpose 2.0.22.0 (STOE, 2016), *X-AREA* Recipe 1.33.0.0 (STOE, 2015), *X-AREA* Integrate 1.72.0.0 (STOE, 2018) *X-AREA X-RED32* 1.63.4.0 (STOE, 2017), ShelXT (Sheldrick, 2015), *SHELXL* (Sheldrick, 2015), Olex2.

References

1. O. V. Dolomanov, L. J. Bourhis, R. J. Gildea, J. A. K. Howard, H. Puschmann, *OLEX2*: a Complete Structure Solution, Refinement And Analysis Program, *J. Appl. Crystallogr.*, **2009**, *42*, 339–341.
2. G. M. Sheldrick, *SHELXT* – Integrated Space-Group And Crystal-Structure Determination, *Acta Cryst. A*, **2015**, *71*, 3–8.

Computing details

Data collection: *X-AREA* WinXpose 2.0.22.0 (STOE, 2016); cell refinement: *X-AREA* Recipe 1.33.0.0 (STOE, 2015); data reduction: *X-AREA* Integrate 1.72.0.0 (STOE, 2018) *X-AREA X-RED32* 1.63.4.0 (STOE, 2017); program(s) used to solve structure: ShelXT (Sheldrick, 2015); molecular graphics: Olex2 (Dolomanov *et al.*, 2009); software used to prepare material for publication: Olex2 (Dolomanov *et al.*, 2009).

Crystal data

**C21H21N5O2**	**D_x = 1.366 Mg m^{-3}**
M_r = 375.43	Mo *K* radiation, = 0.71073 Å
Orthorhombic, *Pbca*	Cell parameters from 18278 reflections
a = 9.4220 (4) Å	= 2.4–29.6°
b = 16.9763 (6) Å	= 0.09 mm^{-1}
c = 22.8215 (7) Å	*T* = 120 K
V = 3650.3 (2) Å^3	Plate, red
Z = 8	0.42 × 0.11 × 0.02 mm
F(000) = 1584	

Data collection

**STOE IPDS 2T diffractometer**	**3615 reflections with *I* > 2 (*I*)**
Radiation source: GeniX Mo, 0.05 x 0.05 mm2 microfocus	R_{int} = 0.046
Detector resolution: 6.67 pixels mm^{-1}	max = 29.3°, min = 2.4°
rotation method, scans	*h* = -12 12
19075 measured reflections	*k* = -23 23
4927 independent reflections	*l* = -31 27

Refinement

**Refinement on F^2**	**0 restraints**
Least-squares matrix: full	Hydrogen site location: inferred from neighbouring sites
$R[F^2 > 2\ (F^2)]$ = 0.060	H-atom parameters constrained
$wR(F^2)$ = 0.164	$w = 1/[\ ^2(F_o^2) + (0.0808P)^2 + 2.2036P]$ where $P = (F_o^2 + 2F_c^2)/3$
S = 1.02	(/)$_{max}$ = 0.001
4927 reflections	max = 0.27 e Å^{-3}
253 parameters	$_{min}$ = -0.27 e Å^{-3}

Special details

Geometry. All esds (except the esd in the dihedral angle between two l.s. planes) are estimated using the full covariance matrix. The cell esds are taken into account individually in the estimation of esds in distances, angles and torsion angles; correlations between esds in cell parameters are only used when they are defined by crystal symmetry. An approximate (isotropic) treatment of cell esds is used for estimating esds involving l.s. planes.

Fractional atomic coordinates and isotropic or equivalent isotropic displacement parameters (Å²) for 3

	x	*Y*	*z*	*U*iso*/*U*eq
O1	0.62322 (14)	0.72560 (8)	0.30775 (6)	0.0342 (3)
O2	0.77335 (15)	0.51174 (8)	0.45843 (6)	0.0344 (3)
N1	0.47862 (15)	0.70579 (9)	0.49440 (7)	0.0287 (3)
H1	0.532558	0.683175	0.467790	0.034*
N2	0.39166 (16)	0.81287 (9)	0.43412 (7)	0.0296 (3)
N3	0.46471 (16)	0.78006 (9)	0.39372 (7)	0.0298 (3)
N4	0.51900 (16)	0.61091 (9)	0.57186 (7)	0.0307 (3)
N5	0.59465 (16)	0.57725 (9)	0.53248 (7)	0.0296 (3)
C1	0.45849 (18)	0.67951 (11)	0.55021 (8)	0.0286 (4)
C2	0.36870 (19)	0.73181 (11)	0.57895 (8)	0.0305 (4)
H2	0.337357	0.728078	0.618418	0.037*
C3	0.33319 (19)	0.79092 (11)	0.53887 (8)	0.0305 (4)
H3	0.273675	0.835054	0.546028	0.037*
C4	0.40139 (18)	0.77290 (10)	0.48660 (8)	0.0287 (4)
C5	0.44893 (19)	0.81576 (11)	0.33819 (8)	0.0294 (4)
C6	0.3538 (2)	0.87684 (11)	0.32641 (9)	0.0323 (4)
H6	0.298631	0.898530	0.357286	0.039*
C7	0.3394 (2)	0.90600 (12)	0.27012 (9)	0.0354 (4)
H7	0.274722	0.947658	0.262373	0.042*
C8	0.4201 (2)	0.87402 (12)	0.22485 (9)	0.0341 (4)
H8	0.410254	0.894134	0.186214	0.041*
C9	0.5144 (2)	0.81323 (12)	0.23560 (8)	0.0326 (4)
H9	0.567906	0.791416	0.204263	0.039*
C10	0.53128 (18)	0.78383 (11)	0.29228 (8)	0.0301 (4)
C11	0.69492 (19)	0.68315 (12)	0.26206 (8)	0.0331 (4)
H11A	0.739543	0.720344	0.234190	0.040*
H11B	0.626737	0.649808	0.240283	0.040*
C12	0.80697 (19)	0.63245 (11)	0.29093 (8)	0.0319 (4)
H12A	0.877641	0.667703	0.309448	0.038*
H12B	0.856716	0.602473	0.259899	0.038*
C13	0.75518 (19)	0.57391 (11)	0.33730 (8)	0.0312 (4)
H13A	0.699196	0.601925	0.367522	0.037*
H13B	0.693376	0.533812	0.318834	0.037*
C14	0.8833 (2)	0.53390 (12)	0.36567 (9)	0.0356 (4)
H14A	0.938629	0.507700	0.334345	0.043*
H14B	0.944512	0.575425	0.382682	0.043*
C15	0.8536 (2)	0.47390 (12)	0.41279 (9)	0.0366 (4)
H15A	0.798947	0.429280	0.396337	0.044*
H15B	0.943889	0.453233	0.428795	0.044*
C16	0.75189 (19)	0.47145 (11)	0.50935 (8)	0.0307 (4)
C17	0.8169 (2)	0.39993 (11)	0.52282 (9)	0.0339 (4)
H17	0.880147	0.376149	0.495644	0.041*
C18	0.7891 (2)	0.36368 (11)	0.57596 (9)	0.0354 (4)
H18	0.835126	0.315480	0.585218	0.042*
C19	0.6952 (2)	0.39649 (12)	0.61592 (9)	0.0354 (4)
H19	0.675663	0.370426	0.651864	0.042*
C20	0.6301 (2)	0.46745 (11)	0.60301 (8)	0.0328 (4)
H20	0.566068	0.490189	0.630339	0.039*
C21	0.65790 (18)	0.50607 (11)	0.55005 (8)	0.0291 (4)

Atomic displacement parameters (Å²) for 3

	*U*11	*U*22	*U*33	*U*12	*U*13	*U*23

O1	0.0283 (7)	0.0401 (7)	0.0342 (7)	0.0085 (6)	0.0023 (5)	-0.0009 (6)
O2	0.0307 (7)	0.0331 (7)	0.0395 (7)	0.0051 (5)	0.0070 (6)	0.0020 (6)
N1	0.0208 (7)	0.0314 (7)	0.0339 (7)	0.0031 (6)	0.0026 (6)	-0.0009 (6)
N2	0.0208 (7)	0.0327 (7)	0.0354 (8)	-0.0005 (6)	0.0017 (6)	-0.0012 (6)
N3	0.0231 (7)	0.0319 (7)	0.0342 (8)	-0.0002 (6)	0.0008 (6)	-0.0002 (6)
N4	0.0236 (7)	0.0330 (8)	0.0354 (8)	-0.0001 (6)	-0.0001 (6)	-0.0017 (6)
N5	0.0201 (7)	0.0306 (7)	0.0382 (8)	0.0000 (6)	-0.0010 (6)	-0.0025 (6)
C1	0.0208 (8)	0.0308 (8)	0.0342 (9)	-0.0015 (6)	0.0007 (6)	-0.0001 (7)
C2	0.0245 (8)	0.0334 (9)	0.0337 (9)	0.0001 (7)	0.0022 (7)	-0.0020 (7)
C3	0.0223 (8)	0.0325 (9)	0.0368 (9)	0.0009 (7)	0.0017 (7)	-0.0022 (7)
C4	0.0196 (8)	0.0289 (8)	0.0377 (9)	0.0003 (6)	0.0002 (7)	-0.0006 (7)
C5	0.0217 (8)	0.0317 (8)	0.0350 (9)	-0.0022 (7)	-0.0004 (7)	-0.0001 (7)
C6	0.0257 (8)	0.0328 (9)	0.0384 (9)	0.0011 (7)	0.0001 (7)	-0.0004 (8)
C7	0.0294 (9)	0.0354 (9)	0.0414 (10)	0.0020 (8)	-0.0020 (8)	0.0037 (8)
C8	0.0297 (9)	0.0369 (9)	0.0356 (9)	-0.0042 (8)	-0.0032 (7)	0.0037 (8)
C9	0.0257 (8)	0.0378 (9)	0.0343 (9)	-0.0043 (7)	0.0006 (7)	-0.0015 (8)
C10	0.0205 (8)	0.0325 (8)	0.0373 (9)	-0.0013 (7)	-0.0009 (7)	-0.0003 (7)
C11	0.0238 (8)	0.0411 (10)	0.0343 (9)	0.0008 (7)	0.0034 (7)	-0.0034 (8)
C12	0.0218 (8)	0.0376 (9)	0.0364 (9)	0.0009 (7)	0.0032 (7)	-0.0017 (8)
C13	0.0218 (8)	0.0336 (9)	0.0382 (9)	-0.0020 (7)	0.0037 (7)	-0.0044 (8)
C14	0.0240 (8)	0.0390 (10)	0.0438 (10)	0.0039 (8)	0.0059 (7)	-0.0003 (9)
C15	0.0298 (9)	0.0351 (9)	0.0451 (11)	0.0066 (8)	0.0093 (8)	-0.0009 (8)
C16	0.0207 (8)	0.0313 (8)	0.0400 (9)	-0.0018 (7)	-0.0010 (7)	0.0012 (7)
C17	0.0239 (8)	0.0328 (9)	0.0451 (10)	0.0028 (7)	-0.0001 (7)	-0.0004 (8)
C18	0.0255 (9)	0.0323 (9)	0.0483 (11)	0.0011 (7)	-0.0072 (8)	0.0032 (8)
C19	0.0332 (10)	0.0348 (9)	0.0382 (10)	-0.0013 (8)	-0.0046 (8)	0.0024 (8)
C20	0.0278 (9)	0.0342 (9)	0.0364 (9)	-0.0003 (7)	-0.0013 (7)	-0.0008 (8)
C21	0.0200 (8)	0.0292 (8)	0.0381 (9)	-0.0002 (6)	-0.0025 (7)	-0.0013 (7)

Geometric parameters (Å, º) for 3

**O1—C10**	**1.361 (2)**	**C9—H9**	**0.9500**
O1—C11	1.436 (2)	C9—C10	1.396 (3)
O2—C15	1.438 (2)	C11—H11A	0.9900
O2—C16	1.363 (2)	C11—H11B	0.9900
N1—H1	0.8800	C11—C12	1.513 (3)
N1—C1	1.363 (2)	C12—H12A	0.9900
N1—C4	1.364 (2)	C12—H12B	0.9900
N2—N3	1.278 (2)	C12—C13	1.532 (3)
N2—C4	1.380 (2)	C13—H13A	0.9900
N3—C5	1.412 (2)	C13—H13B	0.9900
N4—N5	1.281 (2)	C13—C14	1.529 (3)
N4—C1	1.388 (2)	C14—H14A	0.9900
N5—C21	1.406 (2)	C14—H14B	0.9900
C1—C2	1.391 (2)	C14—C15	1.507 (3)
C2—H2	0.9500	C15—H15A	0.9900
C2—C3	1.398 (3)	C15—H15B	0.9900
C3—H3	0.9500	C16—C17	1.394 (3)
C3—C4	1.389 (3)	C16—C21	1.411 (3)
C5—C6	1.397 (3)	C17—H17	0.9500
C5—C10	1.412 (3)	C17—C18	1.385 (3)
C6—H6	0.9500	C18—H18	0.9500
C6—C7	1.384 (3)	C18—C19	1.387 (3)
C7—H7	0.9500	C19—H19	0.9500
C7—C8	1.393 (3)	C19—C20	1.383 (3)
C8—H8	0.9500	C20—H20	0.9500
C8—C9	1.384 (3)	C20—C21	1.400 (3)

C10—O1—C11	118.38 (14)	C12—C11—H11B	110.3
C16—O2—C15	118.10 (15)	C11—C12—H12A	108.1
C1—N1—H1	125.6	C11—C12—H12B	108.1
C1—N1—C4	108.74 (15)	C11—C12—C13	116.59 (15)
C4—N1—H1	125.6	H12A—C12—H12B	107.3
N3—N2—C4	112.09 (15)	C13—C12—H12A	108.1
N2—N3—C5	113.80 (15)	C13—C12—H12B	108.1
N5—N4—C1	110.68 (15)	C12—C13—H13A	109.8
N4—N5—C21	114.78 (15)	C12—C13—H13B	109.8
N1—C1—N4	123.38 (16)	H13A—C13—H13B	108.3
N1—C1—C2	108.45 (16)	C14—C13—C12	109.22 (15)
N4—C1—C2	128.16 (17)	C14—C13—H13A	109.8

C1—C2—H2	126.4	C14—C13—H13B	109.8
C1—C2—C3	107.16 (16)	C13—C14—H14A	108.0
C3—C2—H2	126.4	C13—C14—H14B	108.0
C2—C3—H3	126.5	H14A—C14—H14B	107.3
C4—C3—C2	107.04 (16)	C15—C14—C13	117.09 (16)
C4—C3—H3	126.5	C15—C14—H14A	108.0
N1—C4—N2	124.04 (16)	C15—C14—H14B	108.0
N1—C4—C3	108.59 (16)	O2—C15—C14	108.21 (15)
N2—C4—C3	127.33 (16)	O2—C15—H15A	110.1
C6—C5—N3	123.97 (17)	O2—C15—H15B	110.1
C6—C5—C10	119.65 (17)	C14—C15—H15A	110.1
C10—C5—N3	116.31 (16)	C14—C15—H15B	110.1
C5—C6—H6	119.7	H15A—C15—H15B	108.4
C7—C6—C5	120.50 (18)	O2—C16—C17	124.03 (17)
C7—C6—H6	119.7	O2—C16—C21	116.42 (16)
C6—C7—H7	120.1	C17—C16—C21	119.55 (17)
C6—C7—C8	119.71 (18)	C16—C17—H17	120.1
C8—C7—H7	120.1	C18—C17—C16	119.78 (18)
C7—C8—H8	119.7	C18—C17—H17	120.1
C9—C8—C7	120.65 (18)	C17—C18—H18	119.4
C9—C8—H8	119.7	C17—C18—C19	121.20 (18)
C8—C9—H9	119.9	C19—C18—H18	119.4
C8—C9—C10	120.27 (18)	C18—C19—H19	120.3
C10—C9—H9	119.9	C20—C19—C18	119.48 (19)
O1—C10—C5	115.84 (16)	C20—C19—H19	120.3
O1—C10—C9	124.94 (17)	C19—C20—H20	119.7
C9—C10—C5	119.21 (17)	C19—C20—C21	120.60 (18)
O1—C11—H11A	110.3	C21—C20—H20	119.7
O1—C11—H11B	110.3	N5—C21—C16	115.87 (16)
O1—C11—C12	107.31 (15)	C20—C21—N5	124.72 (17)
H11A—C11—H11B	108.5	C20—C21—C16	119.37 (17)
C12—C11—H11A	110.3		

O1—C11—C12—C13	57.5 (2)	C5—C6—C7—C8	0.2 (3)
O2—C16—C17—C18	179.56 (17)	C6—C5—C10—O1	178.75 (16)
O2—C16—C21—N5	1.6 (2)	C6—C5—C10—C9	-1.0 (3)
O2—C16—C21—C20	179.44 (16)	C6—C7—C8—C9	0.1 (3)
N1—C1—C2—C3	0.5 (2)	C7—C8—C9—C10	-0.9 (3)
N2—N3—C5—C6	-5.3 (3)	C8—C9—C10—O1	-178.42 (17)
N2—N3—C5—C10	177.70 (15)	C8—C9—C10—C5	1.3 (3)
N3—N2—C4—N1	-0.6 (2)	C10—O1—C11—C12	170.67 (15)
N3—N2—C4—C3	-178.20 (17)	C10—C5—C6—C7	0.3 (3)

N3—C5—C6—C7	-176.65 (17)	C11—O1—C10—C5	171.24 (15)
N3—C5—C10—O1	-4.1 (2)	C11—O1—C10—C9	-9.0 (3)
N3—C5—C10—C9	176.17 (16)	C11—C12—C13—C14	-174.57 (16)
N4—N5—C21—C16	-174.52 (16)	C12—C13—C14—C15	179.94 (16)
N4—N5—C21—C20	7.7 (3)	C13—C14—C15—O2	-57.9 (2)
N4—C1—C2—C3	-178.70 (17)	C15—O2—C16—C17	8.1 (3)
N5—N4—C1—N1	0.1 (2)	C15—O2—C16—C21	-172.16 (17)
N5—N4—C1—C2	179.18 (18)	C16—O2—C15—C14	-170.87 (16)
C1—N1—C4—N2	-176.54 (16)	C16—C17—C18—C19	1.2 (3)
C1—N1—C4—C3	1.4 (2)	C17—C16—C21—N5	-178.69 (16)
C1—N4—N5—C21	-178.64 (14)	C17—C16—C21—C20	-0.8 (3)
C1—C2—C3—C4	0.4 (2)	C17—C18—C19—C20	-1.3 (3)
C2—C3—C4—N1	-1.1 (2)	C18—C19—C20—C21	0.2 (3)
C2—C3—C4—N2	176.75 (17)	C19—C20—C21—N5	178.45 (17)
C4—N1—C1—N4	178.05 (16)	C19—C20—C21—C16	0.8 (3)
C4—N1—C1—C2	-1.2 (2)	C21—C16—C17—C18	-0.2 (3)
C4—N2—N3—C5	175.42 (15)		
